# ﻿Benthic megafauna of the western Clarion-Clipperton Zone, Pacific Ocean

**DOI:** 10.3897/zookeys.1113.82172

**Published:** 2022-07-18

**Authors:** Guadalupe Bribiesca-Contreras, Thomas G. Dahlgren, Diva J. Amon, Stephen Cairns, Regan Drennan, Jennifer M. Durden, Marc P. Eléaume, Andrew M. Hosie, Antonina Kremenetskaia, Kirsty McQuaid, Timothy D. O’Hara, Muriel Rabone, Erik Simon-Lledó, Craig R. Smith, Les Watling, Helena Wiklund, Adrian G. Glover

**Affiliations:** 1 Life Sciences Department, Natural History Museum, London, UK Life Sciences Department, Natural History Museum London United Kingdom; 2 Department of Marine Sciences, University of Gothenburg, Gothenburg, Sweden University of Gothenburg Gothenburg Sweden; 3 Norwegian Research Centre, NORCE, Bergen, Norway Norwegian Research Centre, NORCE Bergen Norway; 4 SpeSeas, D’Abadie, Trinidad and Tobago SpeSeas D’Abadie Trinidad and Tobago; 5 Department of Invertebrate Zoology, National Museum of Natural History, Smithsonian Institution, Washington, D.C., USA National Museum of Natural History, Smithsonian Institution Washington United States of America; 6 National Oceanography Centre, Southampton, UK Life Sciences Department, Natural History Museum London United Kingdom; 7 UMR ISYEB, Départment Origines et Évolution, Muséum national d’Histoire Naturelle, Paris, France National Oceanography Centre Southampton United Kingdom; 8 Collections & Research, Western Australia Museum, Perth, Australia Départment Origines et Évolution, Muséum national d’Histoire Naturelle Paris France; 9 Shirshov Institute of Oceanology, Russian Academy of Sciences, Moscow, Russia Collections & Research, Western Australia Museum Perth Australia; 10 School of Biological and Marine Sciences, University of Plymouth, Plymouth, UK Shirshov Institute of Oceanology, Russian Academy of Sciences Moscow Russia; 11 Museums Victoria, Melbourne, Australia University of Plymouth Plymouth United Kingdom; 12 Department of Oceanography, University of Hawai’i at Mānoa, Honolulu, USA Museums Victoria Melbourne Australia; 13 School of Life Sciences, University of Hawai’i at Mānoa, Honolulu, USA University of Hawai’i at Mānoa Honolulu United States of America

**Keywords:** Biogeography, deep-sea mining, DNA barcoding, DNA taxonomy, megafauna, polymetallic nodules

## Abstract

There is a growing interest in the exploitation of deep-sea mineral deposits, particularly on the abyssal seafloor of the central Pacific Clarion-Clipperton Zone (CCZ), which is rich in polymetallic nodules. In order to effectively manage potential exploitation activities, a thorough understanding of the biodiversity, community structure, species ranges, connectivity, and ecosystem functions across a range of scales is needed. The benthic megafauna plays an important role in the functioning of deep-sea ecosystems and represents an important component of the biodiversity. While megafaunal surveys using video and still images have provided insight into CCZ biodiversity, the collection of faunal samples is needed to confirm species identifications to accurately estimate species richness and species ranges, but faunal collections are very rarely carried out. Using a Remotely Operated Vehicle, 55 specimens of benthic megafauna were collected from seamounts and abyssal plains in three Areas of Particular Environmental Interest (APEI 1, APEI 4, and APEI 7) at 3100–5100 m depth in the western CCZ. Using both morphological and molecular evidence, 48 different morphotypes belonging to five phyla were found, only nine referrable to known species, and 39 species potentially new to science. This work highlights the need for detailed taxonomic studies incorporating genetic data, not only within the CCZ, but in other bathyal, abyssal, and hadal regions, as representative genetic reference libraries that could facilitate the generation of species inventories.

## ﻿Introduction

The Clarion-Clipperton Zone (CCZ) in the central abyssal Pacific has become of great interest for deep-sea mineral extraction. This large area of abyssal seafloor, approximately 6 million km^2^ (Wedding et al. 2013), has the largest concentrations of high-grade polymetallic nodules, representing a vast source of commercially valuable metals such as nickel, copper, and cobalt, many of which are currently used in high-tech and green industries (Hein et al. 2020). Although new technological advances are taking deep-sea mining closer to reality, the impacts of mining activities on deep-sea ecosystems remain of concern and are still poorly understood (Jones et al. 2017). To date, the International Seabed Authority (ISA), which governs seabed mining in this area, has granted 17 exploration contracts to permit baseline surveys and resource assessment (but not commercial mining) in the CCZ, and has adopted an environmental management plan establishing 13 areas where exploitation is currently prohibited (called Areas of Particular Environmental Interest, or APEIs) (Smith et al. 2021). Four of these were recently implemented, but the representativity of the APEI network still needs to be assessed.

During the last few decades, there has been a dramatic increase in the scientific exploration of the CCZ, but our knowledge of the faunal communities associated with nodule fields is still limited, and taxonomic records for the area are scarce (Glover et al. 2018). Although the CCZ was first explored in 1875 by the H.M.S. Challenger (Thomson and Murray 1885), relatively little taxonomic work has been carried out in this vast area and hence very little biogeographic information is available (Simon-Lledó et al. 2020). This is particularly problematic as such information is critical to characterise the biodiversity, biogeographic ranges, and connectivity patterns across the entire CCZ in order to make better predictions about the potential impacts of deep-sea mining. In addition, the APEIs designated to preserve regional biodiversity are severely understudied (Glover et al. 2016a; International Seabed Authority 2020; Jones et al. 2021).

The CCZ abyssal seafloor is rich in topographic features such as hills, troughs, fracture zones, and seamounts (Kaiser et al. 2017). It encompasses many habitats with a range of different environmental conditions such as depth, nodule coverage, sediment composition, bathymetric relief, flow intensification on seamounts, and particulate organic carbon (POC) flux (Wedding et al. 2013; International Seabed Authority 2020; McQuaid et al. 2020; Washburn et al. 2021b). Benthic assemblages have been found to change across the CCZ (Wilson 2017; Bonifácio et al. 2020; Simon-Lledó et al. 2020), with POC flux influencing regional megafaunal community patterns, and local environmental factors (i.e., nodule coverage) and bathymetric features having an effect at local scales (Amon et al. 2016; Simon-Lledó et al. 2019c). Seamounts are abundant in the CCZ, most commonly in the eastern and western ends of the area, with elevations of > 1000 m above the plain, and are a major source of hard-substrate habitat (Wedding et al. 2013). Even though seamounts were hypothesised to provide a potential refugia and to be larval sources of nodule-associated fauna that could aid in recolonising nodule fields, of similar depths, disturbed by mining activities, a recent study suggested that the seamounts sampled in the CCZ appear inadequate as refuge areas (Cuvelier et al. 2020). Nonetheless, the biodiversity of the CCZ seamounts remains largely unknown, with only few having been explored on the eastern (Cuvelier et al. 2020; Jones et al. 2021) and western (Durden et al. 2021) margins.

Large benthic organisms (benthic megafauna) have been prioritised for monitoring deep-sea ecosystems because they can be studied from seabed imagery (Danovaro et al. 2020), provide inferences on trophic interactions, ecosystem functioning (Rex and Etter 2010), and processes of disturbances (Jones et al. 2017) and recovery (Simon-Lledó et al. 2019a). In the CCZ, megafaunal benthic assemblages have been studied almost exclusively from video and still images (e.g., Amon et al. 2016; Simon-Lledó et al. 2019c, 2020; Cuvelier et al. 2020; Durden et al. 2021). While these studies have vastly increased our understanding of biodiversity and community structure, uncertainty remains as to the identity of operational taxonomic units (unique identifiers for different morphospecies) recognised in imaged-based survey, and whether they are conspecific with other known species from elsewhere in the deep sea. It is thus critical to complement spatial/temporal analyses with detailed morphological and DNA-sequence analyses of collected specimens (Amon et al. 2016, 2017b).

The DeepCCZ project was conceived to increase our understanding of faunal assemblages and biodiversity in the western CCZ, targeting both unexplored seamounts and APEIs. Here, we provide the first taxonomic synthesis of western CCZ megafauna, which is also the largest megafaunal faunistic study from anywhere in the CCZ based on collected specimens. We provide morphological descriptions, genetic data, and high-resolution imagery for all megafauna specimens collected, including specimens from both the abyssal plains and seamount habitats. It complements similar studies of the high diversity of megafaunal xenophyophores (Gooday et al. 2020a, b), and imagery-based community analysis of the megafauna in this area (Durden et al. 2021).

## ﻿Materials and methods

**Figure 1.**
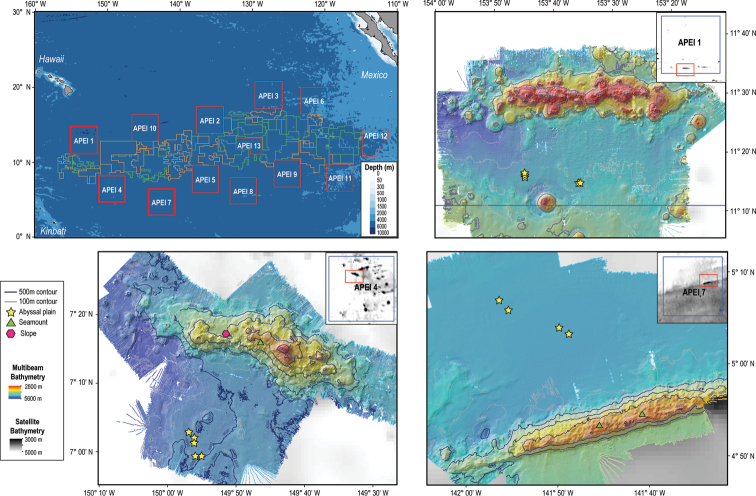
Map of the Clarion-Clipperton Zone (top left) indicating the nine Areas of Particular Environmental Interest (APEIs) in red, exploration areas in green, and reserved areas in orange. Shapefiles were sourced from https://www.naturalearthdata.com/downloads/10m-physical-vectors/10m-bathymetry/, and https://www.isa.org.jm/minerals/maps. Detailed maps of the study areas: APEIs 1 (top right), 4 (bottom left), and 7 (bottom right) show bathymetry from satellite values for the entire APEI, and multibeam values obtained during the DeepCCZ expedition. Sites, and specific geoform, where megafauna samples were collected are indicated as yellow stars in abyssal plains, green triangles in seamounts, and pink hexagons in seamount slopes.

The DeepCCZ expedition, aboard the RV *Kilo Moana*, from 14 May to 16 June 2018, surveyed seamounts and abyssal plains in three Areas of Particular Environmental Interest (**APEIs** 1, 4, and 7) located in the western Clarion-Clipperton Zone (**CCZ**; Fig. 1). All material presented here was collected during this expedition using the Remotely Operated Vehicle (**ROV**) Lu’ukai, and specimens were processed following the DNA taxonomy pipeline described in Glover et al. (2015).

### ﻿Data resources

Sequences generated for this study have been deposited on GenBank: ON400681–ON400730 (COI), ON406602–ON406622 (16S), ON406623–ON406643 (18S), ON406596–ON406601 (28S), and ON411254–ON411256 (ALG11).

### ﻿Sampling

Specimens were selected from across as many taxonomic groups as possible, with duplicates of similar morphotypes avoided; thus, the aim was to increase our understanding of megafaunal diversity. A total of 55 specimens were collected during different dives in three APEIs and three different geoforms (abyssal plain, seamount, and seamount slope), 14 specimens were collected from abyssal seafloor in APEI 1; 13 from abyssal seafloor, two from the seamount slope and six from a seamount in APEI 4; and nine from abyssal seafloor and 11 from a seamount in APEI 7 (Fig. 1). In situ photographs and/or video frame grabs of each specimen were captured, with parallel lasers for scale at 15 cm spacing. Megafaunal specimens were collected with the manipulator (‘Orion’), suction sampler, or push cores, depending on the characteristics of each specimen in order to best preserve morphological characters. Specimens collected with the manipulator were placed into the biobox receptacle, while those collected with the suction sampler were stored in a suction box. Collection data were recorded at the time of capture (e.g., date and time, ROV latitude/longitude, seabed water depth, ROV waypoint name).

After ROV recovery, specimens were transferred and maintained in cold (2–4 °C), filtered seawater until processed. Following Glover et al. (2015), all specimens were photographed, given a preliminary identification and assigned a unique voucher code (e.g., CCZ_020). A tissue sample was taken from each specimen for downstream molecular analyses, and stored in 95% non-denatured ethanol at -20 °C. Specimens were fixed in 10% borax-buffered formalin and transferred to 70% non-denatured ethanol after 48 h, except for sponges, which were kept frozen at -80 °C. After the expedition, specimens and tissue sub-samples were sent to the University of Hawai’i Manoa. Specimens were archived at the Natural History Museum, London (**NHMUK**), and the Western Australian Museum (**WAM**), following taxonomic inspection.

### ﻿DNA extraction, amplification, and sequencing

DNA extraction was performed using the DNeasy Blood and Tissue Kit (Qiagen). The barcode gene cytochrome oxidase I (COI) was the main target because this gene has been used in previous studies on megafauna in the CCZ (e.g., Dahlgren et al. 2016; Glover et al. 2016b). Additional markers for specific taxa (16S, 18S, 28S, and ALG11) were amplified when either COI amplification was unsuccessful or to improve identification or phylogenetic inference. The PCR mix for each reaction contained 10.5 µl of Red Taq DNA Polymerase 1.1X MasterMix (VWR), 0.5 µl of each primer (10 µM), and 1 µl of DNA template. PCR protocols and primers used for COI, 16S, and 18S were following Glover et al. (2016b) and Dahlgren et al. (2016), and Hestetun et al. (2016) for ALG11 and 28S. The PCR products were purified and sequenced at the NHM Sequencing Facilities using a Millipore Multiscreen 96-well PCR Purification System and ABI 3730XL DNA Analyser (Applied Biosystems), respectively. Sequencing primers used were the same as those for the PCR reactions, with the addition of internal primers for 18S and only using primers from the second PCR for the ALG11 gene. DNA sequences were analysed using Geneious 7.0.6 (https://www.geneious.com), with contigs assembled from both forward and reverse sequences and ambiguous base calls corrected manually. DNA sequences generated in this study were submitted to GenBank, with accession numbers ON400681–ON400730 for COI, ON406602–ON406622 for 16S, ON406623–ON406643 for 18S, ON406596–ON406601 for 28S, and ON411254–ON411256 for ALG11.

### ﻿Phylogenetic assignments

Phylogenetic relationships of the western CCZ megafauna were explored by estimating phylogenetic trees for all taxa at different taxonomic levels: phylum Annelida: family Aphroditidae; phylum Arthropoda: order Scalpellomorpha; phylum Cnidaria: order Actiniaria, subclass Ceriantharia, subclass Octocorallia, and class Scyphozoa; phylum Echinodermata: class Asteroidea, class Crinoidea, class Echinoidea, class Holothuroidea, and class Ophiuroidea; and phylum Porifera. Different sets of genes were used depending on published phylogenies and publicly available sequences for each taxon, considering both nuclear and mitochondrial genes if available. For each taxon, sequences were obtained from GenBank (Suppl. material 1: Table S1), except for Porifera as a published alignment was used (Dohrmann 2018). Protein-coding genes (COI, cytochrome oxidase III: COX3, mtMutS homolog: msh1, NADH-dehydrogenase subunit 2 gene: ND2) were aligned using MUSCLE in MEGA-X. Non-protein-coding genes (12S, 16S, 18S, 28S) were aligned using MAFFT v. 7 (Katoh et al. 2019) using the auto strategy, and unalignable regions filtered in GBLOCKS (Castresana 2000), allowing gap positions within final blocks and less strict flanking positions. Individual gene-alignments were concatenated in Geneious, and the best substitution model for each partition was determined using PartitionFinder 2 (Lanfear et al. 2017). For Porifera, we manually aligned our sequences for the 18S, 28S, 16S, and COI genes with the alignment provided in Dohrmann (2018).

Phylogenetic trees were estimated using partitioned maximum-likelihood (RAxML v8.2.10; Stamatakis 2006) and Bayesian inference (BEAST v. 2.4.7; Bouckaert et al. 2014), with the best inferred substitution model for each partition. In RAxML, the most common substitution model for each taxon was selected. RNA secondary structure, as in Dohrmann (2018), was also considered for Porifera, using the S16+G substitution model to paired sites of 18S and 28S. BEAST analyses were performed with trees and clock models linked, a Yule tree model, and relaxed clock log normal. Two independent runs of a maximum 100 M steps were combined after discarding 20% as burn-in. Runs were checked for convergence and a median consensus tree was estimated from the combined post-burn-in samples.

### ﻿Taxonomic assignments

Taxonomic assignments considered information drawn from both molecular and morphological analyses. For the latter, the collected specimens were sent to expert taxonomists for morphological assignments. We assigned every specimen to the lowest Operational Taxonomic Unit (**OTU**), each representing a species. However, we took a precautionary approach when assigning species names (Dahlgren et al. 2016; Glover et al. 2016b; Horton et al. 2021), therefore recording species as ‘cf.’ when uncertain about their identity based on (i) differences in morphological characters, (ii) missing type locality DNA data, or (iii) when type localities are at significantly different depths or vast distances from the western CCZ. Also, those species that could not be identified as a described species were given a unique identifier using the lowest taxonomic level confidently identified and the voucher code (assigned at sea; i.e., CCZ_060). In cases where more than one specimen represented a species with a unique identifier, this only included the voucher code of the best-preserved voucher specimen of that species. Additionally, open taxonomic nomenclature signs were used to indicate that the specimen was not identified any further (‘stet’; e.g., ‘*Laetmonice* stet. CCZ_060’), or when the identification is still uncertain (‘inc’; e.g., Bathymetrinae inc. CCZ_176), and ‘sp.’ was only used for potentially new species (e.g., *Psychronaetes* sp. CCZ_101) after Glover et al. (2016b).

Current records available on OBIS, at a minimum depth of 3000 m, were recovered for each taxon on January 12, 2022 (robis::occurrence; Provoost and Bosch 2020). Records within a box defined by 13°N, 158°W; 18°N, 118°W; 10°N, 112°W; 2°N, 155°W were considered as occurring within the CCZ (Glover et al. 2015).

### ﻿Comparison with seabed-imagery database

To gain preliminary insight into connectivity and distributions, morphology of specimens was compared to and, where possible, aligned with a standardised megafauna morphotype catalogue developed from in situ seabed imagery from across the north Pacific abyss, mostly eastern CCZ (Simon-Lledó et al., pers. obs.). The catalogue aligns invertebrate morphotypes, only for specimens larger than 1 cm, encountered in quantitative megafaunal assessments. At the time of writing, the survey areas so far encompassed in the standardised megafauna catalogue are, from east to west: UK-1 (Amon et al. 2016); BGR, GSR, and APEI 3 (Cuvelier et al. 2020); APEI 6 (Simon-Lledó et al. 2019b); TOML areas B, C, and D (Simon-Lledó et al. 2020); APEIs 1, 4, and 7 (this study; Durden et al. 2021); and the EEZ of Kiribati (Simon-Lledó et al. 2019d). The catalogue assigns each documented taxon a 7-character, unique morphotype code (e.g., POR_001) that differs from unique identifiers used for the species in this study. The level of taxonomic precision achieved in each catalogued taxon is indicated using the open taxonomic nomenclature signs recommended for image-based identifications by Horton et al. (2021). A suffix is added to each morphotype identification specifying the taxonomic rank (e.g., "fam.", "family"; "gen.", "genus" or "sp.", "species") and the signs "indet."or "inc.". The "indet." (*indeterminabilis*) indicates that further identification was not possible as diagnostic features are not typically visible in images, while the "inc." (*incerta*) indicates that despite diagnostic features being visible in images, the identification still has some uncertainty, needing further comparable material for validation.

## ﻿Results

A total of 55 specimens was collected in the western CCZ (Table 1). Based on molecular data these represent 48 species of invertebrate megafauna (43 singletons, four doubletons, and a single species with four representatives) belonging to ten classes in five phyla. However, for three of the doubletons, each specimen was collected in a different APEI, but all were consistently found in the same geoform (i.e., abyssal plain, seamount, or seamount slope). Most of the taxa were collected on the abyssal seafloor (36 specimens from 33 species) > 4800 m deep, followed by seamounts (17 specimens from 13 species) between 3095–3562 m deep, and only two specimens from two species collected on a seamount slope at 4125 m deep. Out of the 48 taxa, only nine were assigned to previously described species, all from adjacent regions such as the Kuril-Kamchatka, Mariana, and Izu-Bonin Trenches, the South China Sea, and other areas of the Northwest and Southwest Pacific.

**Table 1.** Megafauna specimens collected during the DeepCCZ expedition, including details of their collection such as collection site and geoform (S, seamount; AP, abyssal plain; Sl, seamount slope), substrate or attachment (S, on sediment; E, epibiont, N, nodule; C, crust, Sa, anchored to sediment; B, attached to bone), depth, decimal latitude and longitude, scientific collection and accession number, voucher number, and GenBank accession number.

Classification	Species	Site	Substrate / Attachment	Depth (m)	Coordinates (Latitude, Longitude)	Collection	Accession no.	Voucher	GenBank accession no.
**Annelida Polychaeta Phyllodocida** Aphroditidae	*Laetmonice* stet. CCZ_060	APEI 7 (S)	S	3096	4.8897, -141.7500	NHMUK	2022.760	CCZ_060	ON400687 (COI)
**Arthropoda Thecostraca Scalpellomorpha** Scalpellidae	*Trianguloscalpellumgigas*	APEI 7 (AP)	E	4875	5.0442, -141.8165	WAM	C74110	CCZ_074	ON400698 (COI), ON406624 (18S)
	Catherinumcf.albatrossianum	APEI 7 (AP)	E	4875	5.0442, -141.8165	WAM	C74109	CCZ_073	ON400697 (COI), ON406623 (18S)
	Catherinumcf.novaezelandiae	APEI 1 (AP)	E	5241	11.2751, -153.7444	WAM	C74111	CCZ_185	ON400722 (COI), ON406625 (18S)
**Cnidaria Anthozoa** Actiniaria	Metridioidea stet. CCZ_072	APEI 1 (AP)	E	4875	5.0442, -141.8165	NHMUK	2021.19	CCZ_072	ON400696 (COI)
	Metridioidea stet. CCZ_154	APEI 4 (AP)	N	5009	6.9702, -149.9426	NHMUK	2021.27	CCZ_154	ON400715 (COI)
	Metridioidea stet. CCZ_164	APEI 7 (AP)	E	5001	6.9880, -149.9326	NHMUK	2021.5	CCZ_164	ON400717 (COI)
Actinostolidae	Actinostolidae stet. CCZ_183	APEI 1 (AP)	N	5241	11.2751, -153.7444	NHMUK	2021.28	CCZ_183	ON406626 (18S)
	Actinostolidae stet. CCZ_202	APEI 4 (AP)	N	5206	11.2518, -153.6059	NHMUK	2021.22	CCZ_202	ON406627 (18S)
**Scleractinia** Caryophyllidae	Fungiacyathus (Fungiacyathus) cf. fragilis	APEI 4 (S)	S	3562	7.2647, -149.7740	NHMUK	2021.26	CCZ_107	NA
**Alcyonacea** Chrysogorgiidae	*Chrysogorgia* sp. CCZ_112	APEI 4 (Sl)	C	4125	7.2874, -149.8578	NHMUK*		CCZ_112	ON400711 (COI), ON406602 (16S)
Mopseidae	Mopseidae sp. CCZ_088	APEI 4 (AP)	N	5018	7.0089, -149.9109	NHMUK*		CCZ_088	ON400705 (COI), ON406603 (16S)
Primnoidae	*Calyptrophoradistolos*	APEI 4 (Sl)	C	4125	7.2874, -149.8578	USNM	1550968	CCZ_113	ON400712 (COI), ON406604 (16S)
**Pennatulacea** Protoptilidae	*Protoptilum* stet. CCZ_068	APEI 7 (S)	Sa	3096	4.8897, -141.7500	NHMUK	2021.24	CCZ_068	ON400694 (COI), ON406605 (16S)
Spirularia	Spirularia stet. CCZ_067	APEI 7 (S)	Sa	3132	4.8875, -141.7572	NHMUK	2021.23	CCZ_067	ON400693 (COI), ON406606 (16S)
**Scyphozoa Somaeostomeae** Ulmaridae	Ulmaridae stet. CCZ_069	APEI 7 (S)	S	3133	4.8876, -141.7572	NHMUK	2021.25	CCZ_069	ON400695 (COI)
**Echinodermata Asteroidea Brisingida** Freyellidae	Freyasteracf.tuberculata	APEI 4 (AP)	S	5000	6.9879, -149.9123	NHMUK	2022.79	CCZ_087	ON400704 (COI)
		APEI 4 (AP)	S	5000	6.9873, -149.9331	NHMUK	2022.80	CCZ_157	ON400716 (COI)
	*Freyastera* stet. CCZ_201	APEI 1 (AP)	S	5204	11.2518, -153.6059	NHMUK	2022.81	CCZ_201	ON400730 (COI)
**Forcipulatida** Zoroasteridae	*Zoroaster* stet. CCZ_065	APEI 7 (S)	S	3132	4.8877, -141.7569	NHMUK	2022.78	CCZ_065	ON400691 (COI), ON406607 (16S)
**Crinoidea Comatulida** Phrynocrinidae	cf. *Porphyrocrinus* sp. CCZ_165	APEI 4 (AP)	N	5002	6.9879, -149.9327	NHMUK	2022.76	CCZ_165	ON400718 (COI), ON406616 (16S)
Antedonidae	Bathymetrinae incert. CCZ_176	APEI 4 (AP)	E	5009	6.9879, -149.9326	NHMUK	2022.77	CCZ_176	ON400719 (COI), ON406617 (16S);
		APEI 1 (AP)	E	5241	11.2751, -153.7444	NHMUK	2022.60	CCZ_186	ON400723 (COI), ON406618 (16S)
**Echinoidea Aspidodiadematoida** Aspidodiadematidae	Plesiodiademacf.globulosum	APEI 1 (AP)	S	5204	11.2527, -153.5848	CASIZ	229305	CCZ_196	ON400726 (COI), ON406628 (18S)
**Echinothurioida** Kamptosomatidae	*Kamptosomaabyssale*	APEI 4 (AP)	S	5040	7.0360, -149.9395	CASIZ	229306	CCZ_082	ON400701 (COI)
**Holothuroidea Persiculida** Molpadiodemidae	*Molpadiodemas* stet. CCZ_102	APEI 4 (S)	S	3552	7.2701, -149.7827	NHMUK	2022.66	CCZ_102	ON400708 (COI)
	*Molpadiodemas* stet. CCZ_194	APEI 1 (AP)	S	5205	11.2517, -153.6055	NHMUK	2022.71	CCZ_194	ON400725 (COI)
**Synallactida** Synallactidae	*Synallactes* stet. CCZ_153	APEI 4 (AP)	S	5009	6.9704, -149.9426	NHMUK	2022.69	CCZ_153	ON400714 (COI)
	Synallactidae stet. CCZ_061	APEI 7 (S)	S	3132	4.8877, -141.7569	NHMUK	2022.75	CCZ_061	ON400688 (COI), ON406640 (18S)
	Synallactidae stet. CCZ_066	APEI 7 (S)	S	3095	4.8896, -141.7500	NHMUK	2022.63	CCZ_066	ON400692 (COI), ON406642 (18S)
Deimatidae	*Oneirophanta* stet. CCZ_100	APEI 4 (S)	S	3550	7.2647, -149.7740	NHMUK	2022.84	CCZ_100	ON400706 (COI), ON406643 (16S), ON406620 (18S)
	Oneirophantacf.mutabilis	APEI 1 (AP)	S	5203	11.2520, -153.5847	NHMUK	2021.20	CCZ_193	ON400724 (COI), ON406629 (16S), ON406619 (18S)
**Elasipodida** Psychropotidae	*Psychropotesverrucicaudatus*	APEI 4 (AP)	S	4999	6.9878, -149.9119	NHMUK	2021.19	CCZ_086	ON400703 (COI)
	*Psychropotesdyscrita*	APEI 4 (AP)	S	5040	7.0212, -149.9355	NHMUK	2022.83	CCZ_083	ON400702 (COI)
	Benthodytescf.sanguinolenta	APEI 1 (AP)	S	5245	11.2953, -153.7420	NHMUK	2022.70	CCZ_178	ON400720 (COI)
**Elasipodida** Psychropotidae	*Benthodytesmarianensis*	APEI 7 (AP)	S	4861	5.1043, -141.8865	NHMUK	2022.82	CCZ_019	ON400682 (COI)
Elpidiidae	*Peniagoneleander*	APEI 7 (AP)	S	4860	5.1042, -141.8861	NHMUK	2022.61	CCZ_018	ON400681 (COI), ON406621 (16S)
	*Peniagonevitrea*	APEI 7 (AP)	S	4875	5.0442, -141.8164	NHMUK	2022.64	CCZ_077	ON400699 (COI), ON406622 (16S)
Laetmogonidae	*Psychronaetes* sp. CCZ_101	APEI 4 (S)	S	3562	7.2647, -149.7741	NHMUK	2022.65	CCZ_101	ON400707 (COI), ON406631 (18S)
		APEI 4 (S)	S	3562	7.2647, -149.7741	NHMUK	2022.68	CCZ_104	ON400710 (COI), ON406632 (18S)
		APEI 7 (S)	S	3132	4.8877, -141.7570	NHMUK	2022.62	CCZ_063	ON400690 (COI), ON406630 (18S)
		APEI 4 (S)	S	3562	7.2647, -149.7741	NHMUK	2022.67	CCZ_103	ON400709 (COI), ON406639 (18S)
	Laetmogonecf.wyvillethomsoni	APEI 7 (S)	S	3132	4.8877, -141.7569	NHMUK	2021.18	CCZ_062	ON400689 (COI), ON406641 (18S)
**Ophiuroidea Ophioscolecida** Ophioscolecidae	*Ophiocymbiumtanyae*	APEI 1 (AP)	S	5204	11.2523, -153.5848	NHMUK	2022.74	CCZ_206	ON406633 (18S), ON406596 (28S)
	Ophiocymbiumcf.rarispinum	APEI 1 (AP)	S	5206	11.2518, -153.6059	NHMUK	2022.73	CCZ_197	ON400727 (COI)
**Ophiurida** Ophiopyrgidae	Ophiuroglyphacf.irrorata	APEI 7 (S)	S	3239	4.9081, -141.6813	NHMUK	2021.21	CCZ_058	ON400685 (COI)
		APEI 7 (S)	S	3096	4.8897, -141.7500	NHMUK	2022.72	CCZ_059	ON400686 (COI)
**Porifera Hexactinellida Amphidiscosida** Hyalonematidae	*Hyalonema* stet. CCZ_020	APEI 7 (AP)	Sa	4856	5.1149, -141.8967	NHMUK		CCZ_020	ON400683 (COI), ON406634 (18S), ON406608 (16S), ON406597 (28S), ON411254 (ALG11)
		APEI 1 (AP)	Sa	5245	11.2954, -153.7422	NHMUK	2022.8	CCZ_179	ON400721 (COI), ON406609 (16S)
	*Hyalonema* stet. CCZ_081	APEI 4 (AP)	Sa	5031	7.0360, -149.9395	NHMUK	2022.9	CCZ_081	ON406610 (16S)
**Lyssacinosida** Euplectellidae	Euplectellinae stet. CCZ_199	APEI 1 (AP)	Sa	5202	11.2518, -153.5853	NHMUK		CCZ_199	ON400729 (COI), ON406611 (16S)
	*Docosaccus* sp. CCZ_021	APEI 7 (AP)	Sa	4860	5.1043, -141.8867	NHMUK	2022.6	CCZ_021	ON400684 (COI), ON406635 (18S), ON406612 (16S), ON406598 (28S), ON411255 (ALG11)
**Lyssacinosida** Euplectellidae	*Holascus* stet. CCZ_078	APEI 7 (AP)	Sa	4874	5.0443, -141.8162	NHMUK	2022.7	CCZ_078	ON400700 (COI), ON406636 (18S), ON406613 (16S), ON406599 (28S), ON411256 (ALG11)
	Bolosominae stet. CCZ_198	APEI 1 (AP)	Sa	5205	11.2518, -153.6053	NHMUK	2022.10	CCZ_198	ON400728 (COI), ON406637 (18S), ON406614 (16S), ON406600 (28S)
**Sceptrulophora** Euretidae	*Bathyxiphus* sp. CCZ_151	APEI 4 (AP)	B	5001	6.9881, -149.9321	NHMUK		CCZ_151	ON400713 (COI), ON406638 (18S), ON406615 (16S), ON406601 (28S)

* Temporarily stored at University of Hawai’i at Mānoa, Honolulu, USA.

Only two of these nine species had been previously found in the CCZ. Juveniles of the brittle star *Ophiocymbiumtanyae* Martynov, 2010 were collected in the eastern IFREMER contract area and in APEI 3, but due to their early life stage, they lacked taxonomically informative characters and were only assigned to family level using DNA barcoding data (Christodoulou et al. 2020). In this study, genetic data confirmed the taxonomic identity of these specimens. The other species previously known from the CCZ is the sea cucumber *Peniagoneleander* Pawson & Foell, 1986, which also occurs in the Mariana Trench (Gong et al. 2020). Additionally, ten species were assigned as ‘cf.’ based on morphological differences from similar described species, or because prior collections were in other ocean basins or different bathymetric ranges. These, and the remaining 30 taxa, likely represent undescribed species. Based on morphological and genetic evidence, two of these undescribed taxa have also been previously reported for the eastern CCZ (Freyasteracf.benthophila and Crinoidea sp. NHM_055 from Glover et al. (2016b) and (Amon et al. 2017b), referred to herein as Freyasteracf.tuberculata and cf. *Porphyrocrinus* sp. CCZ_165, respectively).

The in situ images taken for 53 specimens were classified into a total of 45 morphotypes using the standardised megafauna imagery catalogue (Simon-Lledó et al., pers. obs.). From these, 11 (24%) were new additions to the existing catalogue, thus representing morphotypes exclusively (to-date) encountered in the western CCZ (i.e., APEIs 1, 4 and 7), while 27 (60%) had already been encountered in other areas. More specifically, nine (20%) of the 45 morphotypes encountered in the western CCZ have also previously been found both in abyssal areas of the Kiribati EEZ (west of the areas studied) and in the eastern CCZ. Two (4%) of the morphotypes encountered in the western CCZ have been found in Kiribati (but not in eastern CCZ locations), whereas 16 (36%) of the western CCZ morphotypes have been encountered in the eastern CCZ, but not in Kiribati.

### ﻿Descriptions

**Phylum Annelida Lamarck, 1809**

**Class Polychaeta Grube, 1850**

**Subclass Errantia Audouin & H Milne Edwards, 1832**

**Order Phyllodocida Dales, 1962**

**Suborder Aphroditiformia Levinsen, 1883**

**Family Aphroditidae Malmgren, 1867**

#### Genus *Laetmonice* Kinberg, 1856

**Figure 2.**
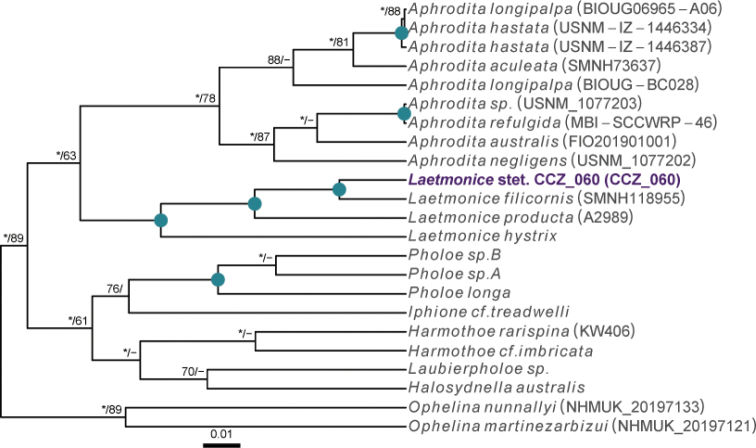
Rooted Bayesian phylogeny for the family Aphroditidae. COI-only BEAST median consensus tree with posterior probability (PP) and bootstrap (BS) values indicated for each node. Only values of PP > 0.70 and BS > 50 are shown, with values of PP > 0.95 and BS > 90 indicated with a circle. Nodes not recovered on the RAxML tree are indicated with a hyphen. Sequences generated in this study are highlighted in violet.

Currently, there are no records from ≥ 3000 m depth for the genus *Laetmonice* Kinberg, 1856, in the Clarion-Clipperton Zone (OBIS 2022). A single polychaete specimen was collected, for which the genetic sequence of the COI gene was generated and used to estimate a COI-only phylogenetic tree (Fig. 2).

##### ﻿ Laetmonice

Taxon classificationAnimaliaPhyllodocidaAphroditidae

stet. CCZ_060

48F3C09C-E045-5C58-9162-DB85853E9B6E

Fig. 3

###### Material.

Clarion-Clipperton Zone • 1 specimen; APEI 7; 4.8897°N, 141.75°W; 3096 m deep; 27 May. 2018; Smith & Durden leg.; GenBank: ON400687 (COI); NHMUK 2022.76; Voucher code: CCZ_060.

###### Description.

**Figure 3.**
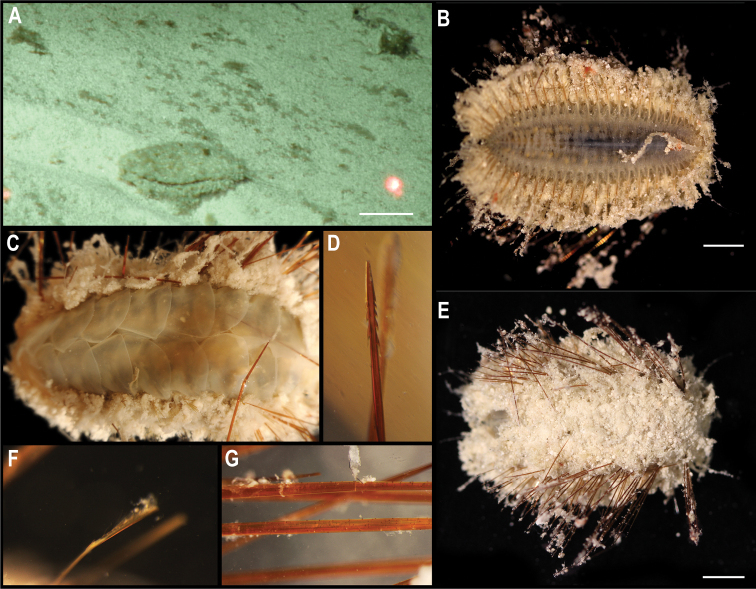
*Laetmonice* stet. CCZ_060 **A** in situ image **B** ventral surface **C** elytra on dorsal surface **D** harpoon-shaped chaeta **E** dorsal surface **F** neurochaeta with fringed tips **G** notochaetal spine shafts. Scale bars: 2cm (**A**); 0.5 cm (**B, E**). Image attribution: Durden and Smith (**A**), Wiklund, Durden, Drennan, and McQuaid (**B, E**), Drennan (**C, D, F, G**).

Single specimen (Fig. 3A). Body short, ovoid, flattened ventrally and somewhat arched dorsally. Specimen ~ 1 cm at widest point and 2 cm long, with 31 chaetigers. Dorsal felt not present. Specimen caked dorsally in dense layer of pale sediment (Fig. 3B, E), easily removed from dorsum but adhering to prostomium, parapodia, chaetae, and pygidium, obscuring respective features. Elytra 15 pairs, semi-translucent, smooth, and overlapping to cover dorsum (Fig. 3C). Dorsal cirri long, fine and tapering, extending beyond parapodia. Ventrum smooth. Ventral cirri, short, mostly broken off, not extending to base of neurochaetae. Parapodia biramous. Notochaetae include long, dark, brassy spines (Fig. 3E) with simple, tapered tips or with harpoon-shaped tips bearing four or five recurved fangs (Fig. 3D); both types of notochaetae with tuberculated shafts (Fig. 3G); neurochaetae include finer, shorter, paler chaetae with subdistal lateral spur and distal fringe of filamentous hairs (Fig. 3F), tips frequently broken off or covered in sediment.

###### Remarks.

The presence of harpoon-shaped notochaetae supports the placement of this specimen within the genus *Laetmonice* (Fauchald 1977). Forms a monophyletic clade with other species of the genus *Laetmonice* based on COI sequences. Genetically distinct from *Laetmonice* stet. CCZ_060, the closest match is with *Laetmonicefilicornis* Kinberg, 1856 (90.8% similarity). *Laetmonicefilicornis* is described from shelf depths near Sweden in the North Atlantic.

###### Ecology.

This specimen was observed crawling on the sedimented seafloor on the seamount of APEI 7 at 3096 m depth.

###### Comparison with image-based catalogue.

No exactly identical Aphroditiformia morphotypes have been so far catalogued from seabed imagery collected in the eastern CCZ or in abyssal areas of the Kiribati EEZ. Consequently, the in situ image of *Laetmonice* stet. CCZ_060 was added as a new morphotype (i.e., *Laetmonice* sp. indet., ANN_019) in the megafauna imagery catalogue. Only one other Aphroditiformia morphotype (i.e., Aphroditidae gen. indet., ANN_022; with much larger spines and no sediment coating), was catalogued from seabed imagery in the eastern CCZ, also found on a seamount. In vertically-facing seabed images, Aphroditiformia morphotypes could potentially be confused with plate-shaped Xenophyophore tests (e.g., Psamminidae), particularly a dense layer of sediment is found coating specimens, as observed in *Laetmonice* stet. CCZ_060 (Fig. 3A).

**Phylum Arthropoda von Siebold, 1848**

**Subphylum Crustacea Brünnich, 1772**

**Superclass Multicrustacea Regier, Shultz, Zwick, Hussey, Ball, Wetzer, Martin & Cunningham, 2010**

**Class Thecostraca Gruvel, 1905**

**Subclass Cirripedia Burmeister, 1834**

**Infraclass Thoracica Darwin, 1854**

**Superorder Thoracicalcarea Gale, 2015**

**Order Scalpellomorpha Buckeridge & Newman, 2006**

**Family Scalpellidae Pilsbry, 1907**

**Figure 4.**
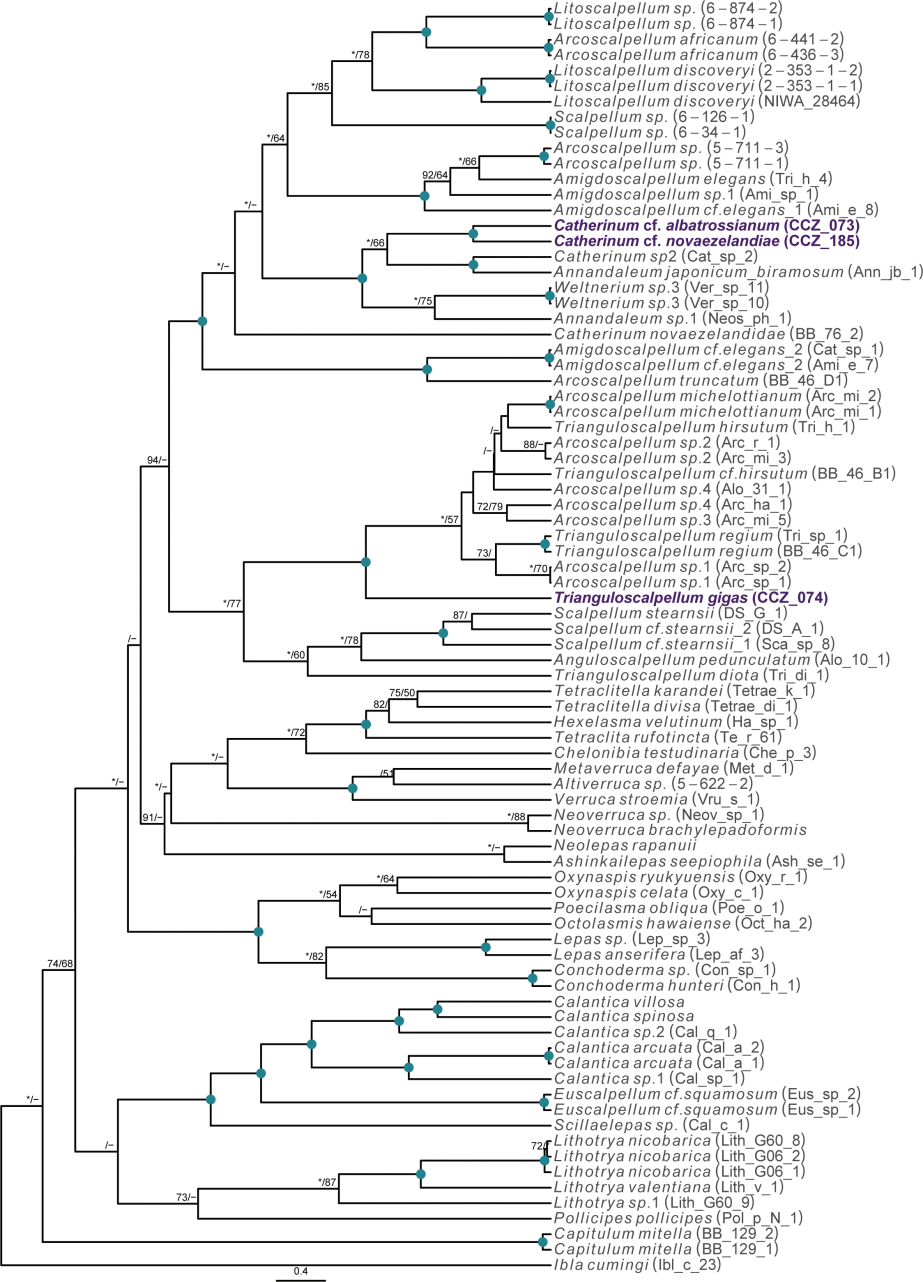
Rooted Bayesian phylogeny of Scalpellomorpha. Concatenated (18S, and COI) BEAST median consensus tree with posterior probability (PP) and bootstrap (BS) values indicated. Only values of PP > 0.70 and BS > 50 are shown, with values of PP > 0.95 and BS > 90 indicated with a circle. Nodes not recovered on the RAxML tree are indicated with a hyphen. Sequences generated in this study are highlighted in violet.

To date, there is a single record at > 3,000 m depth for the order Scalpellomorpha in the CCZ (OBIS 2022), but no collected material. Three specimens were collected during the DeepCCZ expedition; these belong to three different species from which only one was confidently assigned to a previously described species. Sequences for the COI and 18S genes were generated for the three specimens and included in a phylogenetic tree estimated from 18S and COI sequences (Fig. 4).

Scalpellomorpha have been commonly found in image-based megafauna surveys across the north Pacific abyss, usually attached to sponge stalks or nodules. However, their classification beyond family level (e.g., Scalpellidae) from seabed imagery is constrained by their generally small size; only large specimens (> 3 cm) which are rarely encountered can sometimes be classified to genus level from in situ images. Consequently, scalpellid specimens usually are collated into a single, generic morphotype (i.e., Scalpellidae gen. indet., ART_010) in image-based quantitative analyses.

#### Genus *Trianguloscalpellum* Zevina, 1978

##### ﻿ Trianguloscalpellum gigas

Taxon classificationAnimaliaScalpellomorphaScalpellidae

(Hoek, 1883)

EC49AB62-FBC3-5310-AECB-BD700C2ADBE1

Fig. 5

###### Material.

Clarion-Clipperton Zone • 1 specimen; APEI 7; 5.0442°N, 141.8165°W; 4874 m deep; 28 May. 2018; Smith & Durden leg.; GenBank: ON400698 (COI), ON406624 (18S); WAM C74110; Voucher code: CCZ_074.

###### Description.

**Figure 5.**
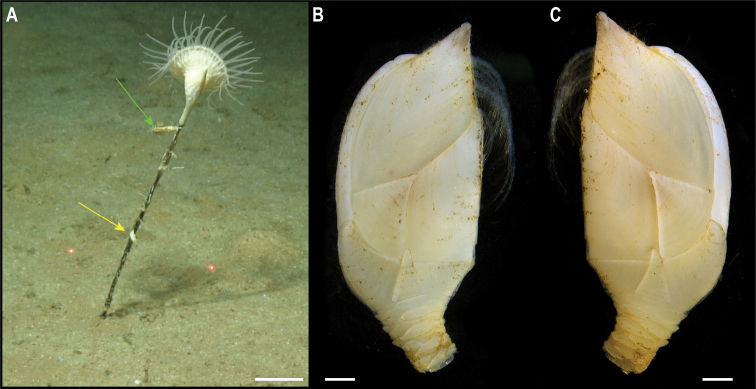
*Trianguloscalpellumgigas* (Hoek, 1883). Specimen CCZ_074: **A** in situ photograph, attached to a glass sponge stalk **B** left **C** and right lateral views. Scale bars: 5 cm (**A**); 1 mm (**B, C**). Image attribution: Durden and Smith (**A**), Hosie (**B, C**). Arrows indicate position of *T.gigas* (specimen CCZ_074; lower, yellow) and Catherinumcf.albatrossianum (specimen CCZ_073; upper, green).

Single specimen, found attached to a glass sponge stalk (Fig. 5A). Capitulum elongated, longer than wide (L = 8 mm, W = 5 mm), white, with short peduncle (2 mm) covered by large scales (Fig. 5B, C). Capitulum is formed by 14 capitular plates, and growth lines are not visible. Carina is simply bowed, narrowing distally but being approx. the same breath proximally. The tergum is somewhat oval-shaped, long, ~ 2× as long as wide, with pointed basal angle, carinal margin arched, and occludent margin straight. Scutum is somewhat quadrangular, broad, 1.5× as long as wide, with occludent margin much longer than the lateral margin. Inframedian latus is triangular, reaching upper latus. Carinolatus triangular, umbo apical, higher than rostrolatus.

###### Remarks.

The specimen appears to be a juvenile of the species *T.gigas* based on the plate arrangement, although diagnostic characters are not fully developed. There are no sequences available on public databases for *T.gigas*, but the 18S gene sequence is very similar (> 99%) to other species within the family Scalpellidae, mostly within the subfamily Arcoscalpellinae. However, the COI sequence is highly divergent (> 15% nucleotide divergence and > 3% amino-acid divergence) from published sequences of other species within the subfamily. The phylogenetic tree from concatenated data for COI and 18S recovered a well-supported clade of species of *Anguloscalpellum* and *Trianguloscalpellum*, but did not recover the genera as monophyletic. The type material for *T.gigas* was collected during the H.M.S. Challenger expedition in the middle of the North Pacific (Station 246: 36.1667°N, 178.0°E) at 3749 m depth (Hoek 1883). The species has been recorded from the Northwest and Southwest Pacific, and the Indian Ocean, from 3310 to 4820 m depth (Shalaeva and Boxshall 2014).

###### Ecology.

The specimen was collected in the sedimented abyssal plain of APEI 7, at 4874 m depth. It was attached to a glass sponge stalk, along with another barnacle (Catherinumcf.albatrossianum; specimen CCZ_073), and an anemone (Metridioidea stet. CCZ_072; specimen CCZ_072).

###### Comparison with image-based catalogue.

No exactly similar Scalpellidae morphotypes have been so far catalogued from seabed imagery collected in the eastern CCZ or in abyssal areas of the Kiribati EEZ. Consequently, the in situ image of *Trianguloscalpellumgigas* was catalogued as a new morphotype (i.e., *Trianguloscalpellumgigas* sp. inc., ART_033). However, given the small size of specimen CCZ_074, this morphotype could have easily been i.e., undetected in seabed image surveys conducted in other areas of the CCZ.

#### Genus *Catherinum* Zevina, 1978

##### ﻿ Catherinum cf. albatrossianum

Taxon classificationAnimaliaScalpellomorphaScalpellidae

(Pilsbry, 1907)

240FAAF3-7DA1-5CE0-9BE4-8C34CBA16ACF

Fig. 6

###### Material.

Clarion-Clipperton Zone • 1 specimen; APEI 7; 5.0442°N, 141.8165°W; 4875 m deep; 28 May. 2018; Smith & Durden leg.; GenBank: ON400697 (COI), ON406623 (18S); WAM C74109; Voucher code: CCZ_073.

###### Description.

**Figure 6.**
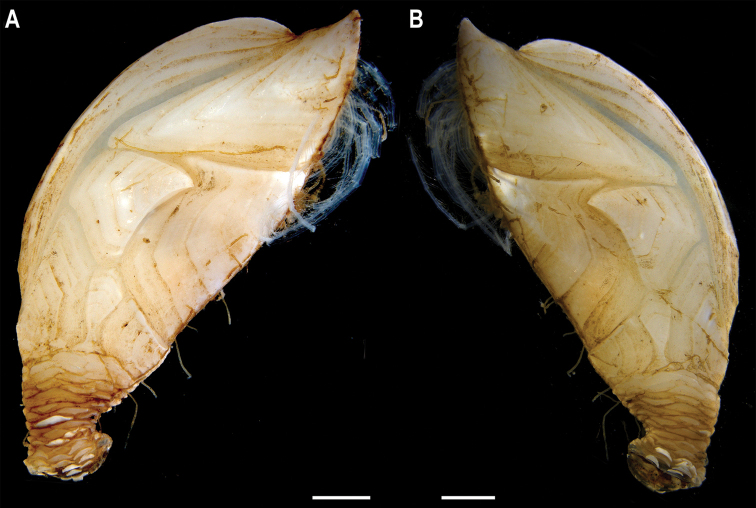
Catherinumcf.albatrossianum (Pilsbry, 1907). Specimen CCZ_073: **A** left **B** right lateral views. Scale bars: 2 mm. Image attribution: Hosie (**A, B**).

Single specimen 21 mm long, attached to glass sponge stalk (Fig. 5A; upper, green arrow). Capitulum elongated, white, ~ 2× as long as wide (L = 16 mm, W = 8 mm); widest in the middle, tapering towards summit and base; short peduncle (4 mm) with small scales (Fig. 6A, B). Fourteen capitular plates fully calcified, showing growth lines, and separated by very narrow chitinous spaces. Carina is strongly arched in the distal half, tapering proximally, with flat roof and apical umbo. Tergum is almost a right triangle, longer than wide, with slightly convex occludent margin. Scutum is more than twice as wide as long, with arcuate occludent margin, with a distal indent on the lateral margin for the reception of the apex of the upper latus; baso-lateral margin rounded and next to the infra-median latus. Upper latus is pentagonal; with apical umbo projecting into notch on the scutum; scutal margin in concave; very short basal margin and carinolateral margin longer than carinal margin. Rostrolatus has an umbo projecting from the rostral margin. Rostrum minute. Large carinolatus, ~ 2× as long as wide, umbo sub-basal, abutting base of carina, apex slightly extending approximately one fifth of the carina. Inframedian latus is > 2× as long as the widest section, widest distally and with rostral and carinal margins concave, with umbo sub-basal.

###### Remarks.

Morphological characters are in accordance with the description of *C.albatrossianum*. The 18S sequence matches three genera within the subfamily Arcoscalpellinae Zevina, 1978, while the closest match (85% similarity) for the COI sequence is to another species of *Catherinum*. Like, C.cf.novaezelandiae it differs morphologically from *C.tortilum*, reported from the CCZ by Poltarukha and Mel’Nik (2012), in the form of the inframedian latus. The type locality of *C.albatrossianum* is off Cape Hatteras, in the northwest Atlantic, at ~ 3740 m depth, but it has been reported for the North Atlantic, Gulf of Mexico, and Indian Ocean between 760 and 4180 m depth (Zevina and Poltarukha 2014). The original description states that the species lacks a rostrum, however, a minute rostrum is present in the specimen examined herein. This in addition to the documented range of this species is the reason for the use of cf. in the identification.

###### Ecology.

Specimen was collected in a muddy abyssal area of APEI 7, at 4874 m depth. It was attached to a glass sponge stalk (Fig. 5A; upper, green arrow), along with another barnacle (*Trianguloscalpellumgigas*, specimen CCZ_074; lower, yellow arrow), and an anemone (Metridioidea stet. CCZ_072; specimen CCZ_072). It had hydrozoans and two serpulid polychaetes attached to it.

###### Comparison with image-based catalogue.

A very similar morphotype (*Catherinum* sp. indet., ART_032) has been encountered (e.g., large specimens > 3 cm in length) in seabed image surveys conducted across the eastern CCZ and in abyssal areas of the Kiribati EEZ.

##### ﻿ Catherinum cf. novaezelandiae

Taxon classificationAnimaliaScalpellomorphaScalpellidae

(Hoek, 1883)

45B4C558-1B9F-5460-8D13-2E3E3017FD00

Fig. 7

###### Material.

Clarion-Clipperton Zone • 1 specimen; APEI 1; 11.2751°N, 153.7444°W; 5241 m deep; 09 Jun. 2018; Smith & Durden leg.; GenBank: ON400722 (COI), ON406625 (18S); WAM C74111; Voucher code: CCZ_185.

###### Description.

**Figure 7.**
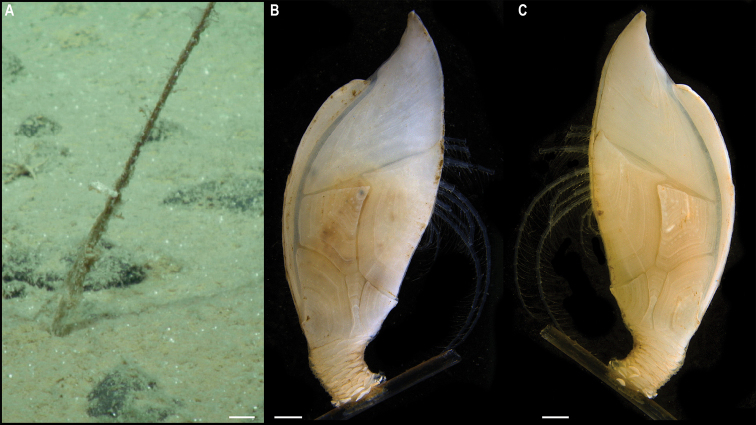
Catherinumcf.novaezelandiae (Hoek, 1883). Specimen CCZ_185: **A** in situ photograph **B** right **C** left lateral views. Scale bars: 1 cm (**A**); 1 mm (**B, C**). Image attribution: Durden and Smith (**A**), Hosie (**B, C**).

Single specimen 14 mm long; with elongated, white capitulum, > 2× as long as wide (L = 12 mm, W = 5 mm), and short peduncle (2 mm) with small scales (Fig. 7). Capitulum consists of 14 fully calcified capitular plates with growth lines, separated from each other by narrow chitinous sutures. Carina is simply bowed, with flat roof. Tergum is triangular, shorter on the occludent margin, with apical umbo; apical angle is similar to angle between the carinal and scutal margins. Upper latus somewhat pentagonal, with lower edge truncated, and apical edge reaching over the scutum; with apical umbo. Rostrolatus with umbo apical on the rostral margin, and arched lateral margin. Inframedian latus irregular in shape, narrow, almost 3× as long as the widest part, with umbo sub-medial; rostral and carinal margins concave. Carinolatus is large, ~ 2× as long as wide, with umbo sub-carinal, above basal angle.

###### Remarks.

Morphological characters of the capitulum conform to the description of the genus *Catherinum*. The sequence for the 18S gene is similar to sequences from other species within the same family. Another species within the genus, *C.tortilum* (Zevina, 1973), originally described from the Indian Ocean at 2760 m depth has also been recorded for the CCZ at similar depths (4872–4877 m depth; Poltarukha and Mel’Nik 2012). In *C.tortilum*, the inframedian latus’ umbo is conspicuously displaced laterally away from the midline. The species *C.novaezelandiae* is distributed in the Western and Eastern Indian Ocean, Western Central and Southwest Pacific, from depths 455–4800 m (Shalaeva and Boxshall 2014), but was originally described from East Cape, New Zealand (Southwest Pacific), at 1280 m.

###### Ecology.

The specimen was collected in the sedimented abyssal plain of APEI 1 at 5241 m depth. It was attached to a glass sponge stalk, along with a crinoid (Bathymetrinae inc. CCZ_176; specimen CCZ_186), a polychaete, and anemones, that was anchored in the mud.

###### Comparison with image-based catalogue.

Relatively large abundances of a very similar morphotype (*Catherinum* sp. indet., ART_031) were observed in seabed imagery collected within abyssal areas of the Kiribati EEZ, but not in eastern CCZ surveys.

**Phylum Cnidaria Hatschek, 1888**

A total of 12 cnidarians w collected, belonging to six orders in two classes (Anthozoa and Scyphozoa).

**Class Anthozoa Ehrenberg, 1834**

**Subclass Hexacorallia Haeckel, 1896**

**Order Actiniaria Hertwig, 1882**

**Figure 8.**
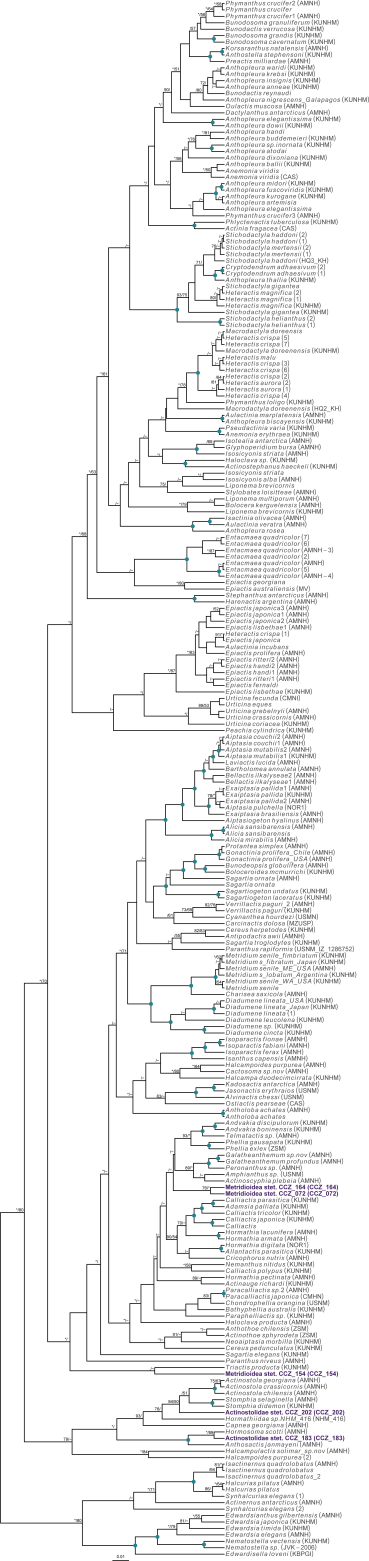
Rooted Bayesian phylogeny of Actiniaria. Concatenated (12S, 16S, 18S, 28S, COI, and COX3) BEAST median consensus tree with posterior probability (PP) and bootstrap (BS) values indicated. Only values of PP > 0.70 and BS > 50 are shown, with values of PP > 0.95 and BS > 90 indicated with a circle. Nodes not recovered on the RAxML tree are indicated with a hyphen. Sequences generated in this study are highlighted in violet.

To date, there are 33 records of Actiniaria found at > 3000 m depth in the CCZ (OBIS 2022), but only two of these represent collected specimens. We collected five specimens, all belonging to different species, and for which genetic sequences of the COI or 18S genes were generated and included in a phylogenetic tree built from a concatenated alignment of 12S, 16S, 18S, 28S, COI, and COX3 (Fig. 8).

**Suborder Enthemonae Rodríguez & Daly in Rodríguez et al. 2014**

**Superfamily Metridioidea Carlgren, 1893**

**Metridioidea stet. CCZ_072**

Fig. 9

**Material.** Clarion-Clipperton Zone • 1 specimen; APEI 1; 5.0442°N, 141.8165°W; 4875 m deep; 28 May. 2018; Smith & Durden leg.; GenBank: ON400696 (COI); NHMUK 2021.19; Voucher code: CCZ_072.

**Figure 9.**
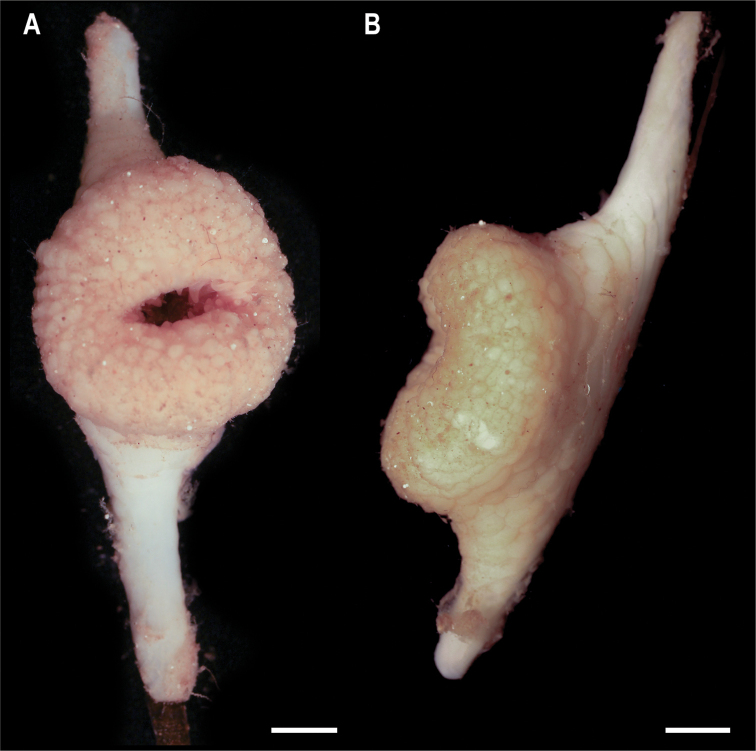
Metridioidea stet. CCZ_072 **A** oral **B** lateral views. Scale bars: 5 mm (**A, B**). Image attribution: Wiklund, Durden, Drennan, and McQuaid (**A, B**).

**Description.** Single specimen, white (Figs 5, 9). Body subcylindrical, pedal disc modified and attached to a glass sponge stalk, oral disc is > 2× column width; with at least two cycles of slender, tapered, long, white tentacles, almost as long as the oral disc diameter (Fig. 5A). Tubercles are evident on the top half of the column when preserved, but tentacles completely retracted (Fig. 9 A, B).

**Remarks.** COI sequence is similar (97.3%) to other species within the subfamily Metridioidea but based on COI we were unable to delimit species because interspecific divergence is very low. Additionally, only a few studies have included sequences for COI, therefore hindering comparisons based solely on this gene. The COI divergence between Metridiodea stet. CCZ_164 and Metridiodea stet. CCZ_072 (1.95% K2P distance) was higher than the genetic distance between other species in the family Metridiodea (Rodriguez et al. 2014), suggesting these to belong to separate species. The phylogenetic tree recovered both CCZ specimens within the subfamily Metridioidea (Fig. 8), in a clade belonging to Cuticulata. Clades within Cuticulata were not well resolved in the phylogeny, but this group includes the Graspina clade (families Amphiantidae, Galantheanthenidae, and Actinoscyphiidae) that is characterised by a modified pedal disc that enables them to attach to other substrates, such as sponge stalks (Rodriguez et al. 2014), and is advantageous in deep-sea ecosystems. Based on this modified pedal disc, the specimen very likely belongs to a family within the Graspina clade.

**Ecology.** The specimen was collected in a muddy abyssal plain in APEI 7, at 4874 m depth. It was attached to a glass sponge stalk (Fig. 5A; top of stalk), along with two barnacles (Catherinumcf.albatrossianum, specimen CCZ_073; and *Trianguloscalpellumgigas*, specimen CCZ_074).

**Comparison with image-based catalogue.** A very similar Actiniaria morphotype (Metridioidea fam. indet., ACT_042) mostly attached to sponge stalks, has been commonly encountered in seabed image surveys conducted across the eastern CCZ but not in abyssal areas of the Kiribati EEZ.

**Metridioidea stet. CCZ_154**

Fig. 10

**Material.** Clarion-Clipperton Zone • 1 specimen; APEI 4; 6.9702°N, 149.9426°W; 5009 m deep; 06 Jun. 2018; Smith & Durden leg.; GenBank: ON400715 (COI); NHMUK 2021.27; Voucher code: CCZ_154.

**Figure 10.**
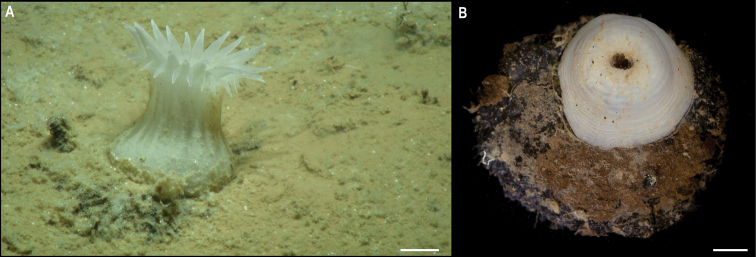
Metridioidea stet. CCZ_154 **A** in situ image **B** specimen before preservation. Scale bars: **A, B** 1 cm. Image attribution: Durden and Smith (**A**), Wiklund, Durden, Drennan, and McQuaid (**B**).

**Description.** Single specimen, completely white when alive (Fig. 10). Body of live specimen is more or less cylindrical, wider proximally and distally, 29 mm long. Pedal disc is the widest, 35 mm in diameter, attached to a manganese nodule, and oral disc 24 mm in diameter (Fig. 10B). Large and small conical, tapered tentacles alternating on the margin of the oral disc in two cycles: ~ 20 + 20, with the larger ones being approx. half the oral disc diameter and located above the smaller tentacles (Fig. 10A). Tentacles are only visible in in situ images (Fig. 10A), as they are fully retracted in the preserved specimen.

**Ecology.** This specimen was attached to a nodule in abyssal sediments in APEI 4 at 5009 m depth.

**Remarks.** The COI sequence is similar to sequences of species within different families, but in the phylogenetic tree it is recovered within the superfamily Metridioidea (Fig. 8).

**Comparison with image-based catalogue.** No similar Actiniaria morphotypes had been catalogued so far from seabed imagery in the eastern CCZ or in abyssal areas of the Kiribati EEZ. The in situ image of Metridiodea stet. CCZ_154 was hence catalogued as a new morphotype (i.e., Metridioidea fam. indet., ACT_044).

**Metridioidea stet. CCZ_164**

Fig. 11

**Material.** Clarion-Clipperton Zone • 1 specimen; APEI 7; 6.988°N, 149.9326°W; 5001 m deep; 06 Jun. 2018; Smith & Durden leg.; GenBank: ON400717 (COI); NHMUK 2021.5; Voucher code: CCZ_164

**Figure 11.**
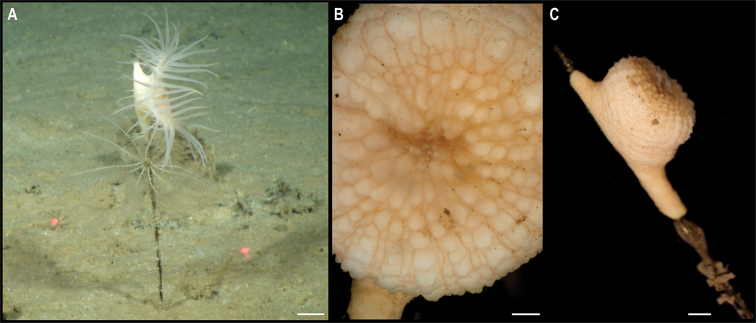
Metridioidea stet. CCZ_164 **A** in situ image of specimen CCZ_164 **B** detail of oral disc **C** lateral view of specimen. Scale bars: 2 cm (**A**); 2 mm (**B**); 5 mm (**C**). Image attribution: Durden and Smith (**A**), Wiklund, Durden, Drennan, and McQuaid (**B, C**).

**Description.** Single specimen, white (Fig. 11A). Specimen with a short, subcylindrical column, with pedal and oral discs almost the same diameter (Fig. 11A). Long, slender, tapered, white tentacles arranged in at least two cycles (Fig. 11A). When preserved, column is more cylindrical, almost as long as wide (H = 18 mm, oral disc diameter = 21 mm), and tubercles are evident on the top half of the column; tentacles completely retracted (Fig. 11B, C).

**Remarks.** COI sequence is very similar to Metridiodea sp. CCZ_072 and they are recovered as sister species, in the multi-gene phylogeny, within the Cuticulata in the superfamily Metridioidea (Fig. 8). This species very likely belongs to a family within the Graspina clade (Amphiantidae, Galantheanthenidae and Actinoscyphiidae) based on the modified pedal disc that allows them to attach to substrates other than rocks (Rodriguez et al. 2014).

**Ecology.** This specimen was collected in muddy abyssal sediments in APEI 4 at 5001 m depth, attached to a glass sponge stalk.

**Comparison with image-based catalogue.** As with specimen from Metridiodea stet. CCZ_072, a very similar morphotype has been commonly found in seabed image surveys conducted across the eastern CCZ (i.e., Metridioidea fam. indet., ACT_042), but it does not seem possible to differentiate between the species Metridiodea stet. CCZ_072 and Metridiodea stet. CCZ_164 from in situ imagery. Morphotype ACT_042 is hence likely to encompass, at least, these two species in image-based analyses conducted across the CCZ.

**Superfamily Actinostoloidea Carlgren, 1932**

**Family Actinostolidae Carlgren, 1932**

**Actinostolidae stet. CCZ_183**

Fig. 12

**Material.** Clarion-Clipperton Zone • 1 specimen; APEI 1; 11.2751°N, 153.7444°W; 5241 m deep; 09 Jun. 2018; Smith & Durden leg.; GenBank: ON406626 (18S); NHMUK 2021.28; Voucher code: CCZ_183.

**Figure 12.**
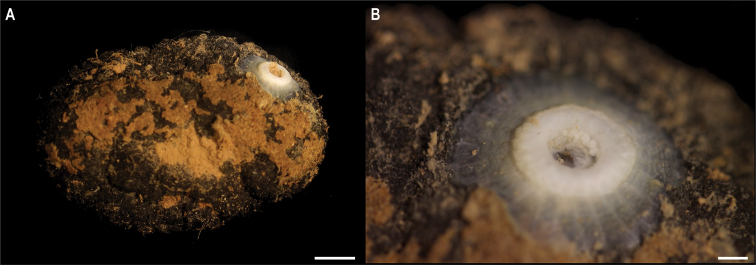
Actinostolidae stet. CCZ_183 **A** specimen attached to nodule **B** close-up of specimen. Scale bars: 1 cm (**A**), 2 mm (**B**). Image attribution: Wiklund, Durden, Drennan, and McQuaid (**A, B**).

**Description.** Single specimen, white, attached to a nodule (Fig. 12A). Column is very short (3 mm), cylindrical (6 mm diameter), pedal disc much wider and completely attached to the nodule. Small tubercles scatter on the column (Fig. 12B).

**Remarks.** Closest matches for the 18S sequence are sequences from other members of the family Actinostolidae (> 99.3%). In the phylogenetic tree, it is also recovered in a well-supported clade with species of the family Actinostolidae (Fig. 8). However, this clade also includes *Capnea*, which has been recovered within the same clade in previous studies (Rodriguez et al. 2014), and a specimen collected in the eastern CCZ identified as a member of the family Hormathiidae (Hormathiidae sp. NHM_416, Dahlgren et al, 2016). No in situ photos are available.

**Ecology.** This specimen was collected in abyssal sediment in APEI 1 at 5241 m depth, attached to a polymetallic nodule.

**Actinostolidae stet. CCZ_202**

Fig. 13

**Material.** Clarion-Clipperton Zone • 1 specimen; APEI 4; 11.2518°N, 153.6059°W; 5206 m deep; 10 Jun. 2018; Smith & Durden leg.; GenBank: ON406627 (18S); NHMUK 2021.22; Voucher code: CCZ_202.

**Figure 13.**
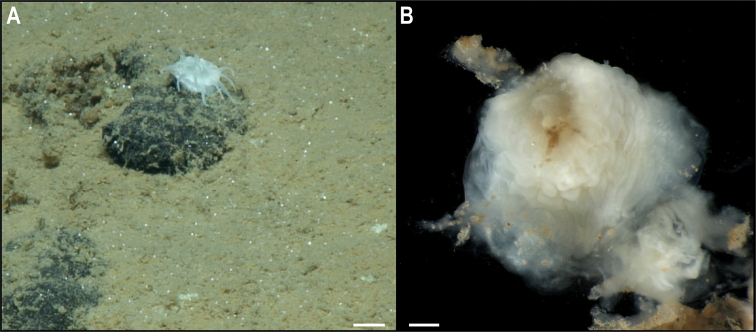
Actinostolidae stet. CCZ_202 **A** in situ image **B** detail of specimen. Scale bars: 1 cm (**A**); 1 mm (**B**). Image attributions: Durden and Smith (**A**), Wiklund, Durden, Drennan, and McQuaid (**B**).

**Description.** Single, white specimen (Fig. 13A). Specimen with short column (4 mm), pedal and oral disc approx. the same diameter (8 mm). Between 8–10 white, long tentacles, approx. as long as the oral disc diameter (Fig. 13A). Column with scale-like pattern in preserved specimen (Fig. 13B), with tentacles fully retracted.

**Remarks.** The closest matches to the 18S sequence are species in different suborders within the Actiniaria (98.3% sequence similarity), including Hormathiidae sp. NHM_416 from the CCZ (Dahlgren et al. 2016). However, in the phylogenetic tree it is confidently recovered within the Actinostolidae (Fig. 8), along with the specimen Hormathiidae sp. NHM_416.

**Ecology.** This specimen was attached to a polymetallic nodule collected in abyssal sediments of APEI 1 at 5206 m depth.

**Comparison with image-based catalogue.** No similar Actiniaria morphotypes have been so far catalogued from seabed imagery in the eastern CCZ or in abyssal areas of the Kiribati EEZ. The in situ image of Actinostolidae stet. CCZ_202 was hence catalogued as a new morphotype (i.e., Actinostolidae gen. indet., ACT_080). However, small actiniarians (e.g., oral disc < 2 cm) are usually difficult to classify from seabed imagery as basic morphological features (e.g., number of tentacles) are often not clearly visible. Consequently, ACT_080 could be potentially confused with similarly small actinian morphotypes commonly encountered in the eastern CCZ (i.e., Hormathiidae gen. inc., ACT_022, also with a short pedal approx. the same diameter as the oral disc, but with 16–18 long thin tentacles).

**Order Scleractinia Bourne, 1900**

For Scleractinia, there are only two records at > 3000 m depth in the CCZ (OBIS 2022), with no specimens collected. A single scleractinian was collected, for which DNA amplification was unsuccessful.

**Family Fungiacyathidae Chevalier & Beauvais, 1987**

#### Genus *Fungiacyathus* Sars, 1872

##### ﻿ Fungiacyathus (Fungiacyathus) cf.fragilis

Taxon classificationAnimaliaScleractiniaFungiacyathidae

Sars, 1872

F3A37DEC-F1A7-5069-9365-4288FFA36963

Fig. 14

###### Material.

Clarion-Clipperton Zone • 1 specimen; APEI 4; 7.2647°N, 149.774°W; 3562 m deep; 03 Jun. 2018; Smith & Durden leg.; NHMUK 2021.26; Voucher code: CCZ_107

###### Description.

**Figure 14.**
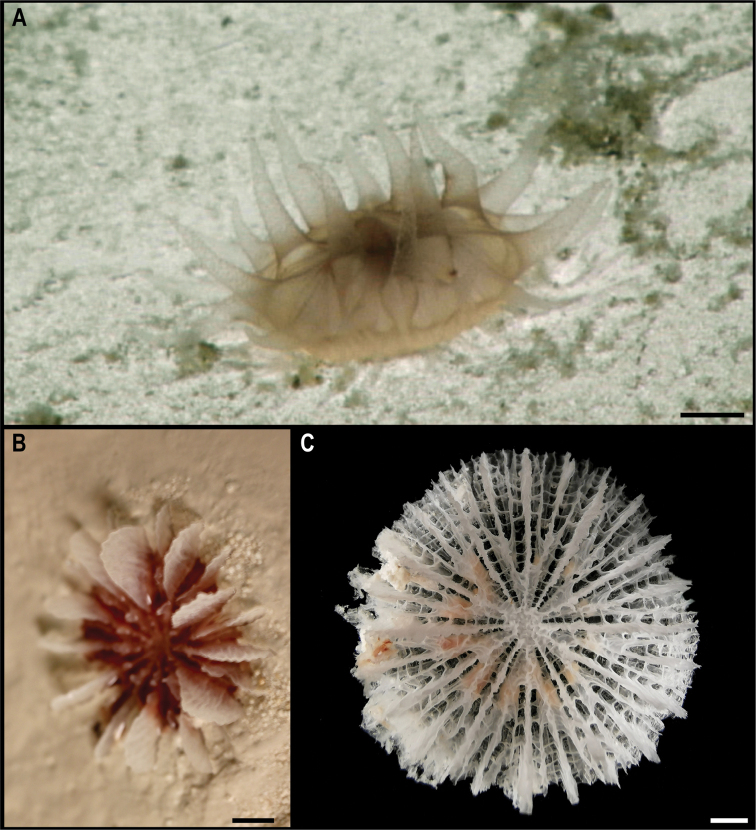
Fungiacyathus (Fungiacyathus) cf. fragilis Sars, 1872. Specimen CCZ_107: **A** in situ image **B** dorsal view of live specimen **C** bleached skeleton. Scale bars: 1 cm (**A**); 3 mm (**B, C**). Image attribution: Durden and Smith (**A**); Wiklund, Durden, Drennan, and McQuaid (**B**); Bribiesca-Contreras (**C**).

Single specimen, solitary, and unattached, ~ 27 mm in transverse diameter. Live specimen with tapered, transparent tentacles, longer than half the corallum diameter and arranged in two or three cycles (Fig. 14A). Corallum is light brown distally and darker proximally on live specimen (Fig. 14B, C). The base is flat and the lower cycle septa are strongly arched upward; septa are arranged in five cycles, those of the fifth are rudimentary.

###### Remarks.

No genetic sequences were obtained from this specimen. Morphological characters match the genus *Fungiacyathus*.

###### Ecology.

This free-living specimen was found on a sedimented area on a seamount in APEI 4, at 3561 m depth.

###### Comparison with image-based catalogue.

A very similar scleractinian morphotype (i.e., *Fungiacyathus* sp. indet., SCL_003) has been encountered in seabed image surveys conducted across the eastern CCZ but not in abyssal areas of the Kiribati EEZ, usually on sediment. As with other solitary scleractinians, this taxon could be confused with an anemone in seabed imagery (e.g., SCL_003 was originally catalogued as an Actiniaria from in situ images, which was addressed following the collection and analysis of the specimen collected in this study).

**Subclass Octocorallia Haeckel, 1866**

**Order Alcyonacea Lamouroux, 1812**

**Figure 15.**
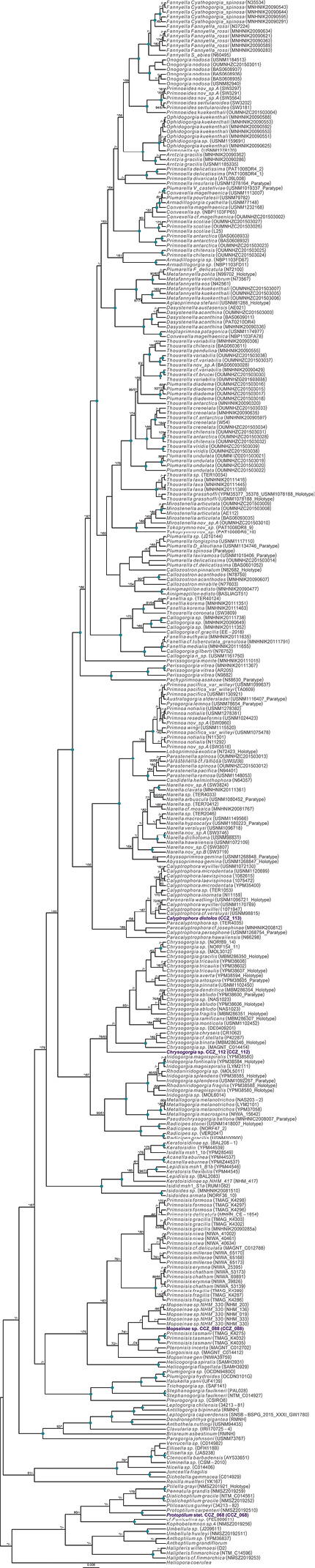
Rooted Bayesian phylogeny of Octocorallia. Concatenated (16S, COI, mtMutS, NADH2) median consensus BEAST tree with posterior probability (PP) and bootstrap (BS) values indicated. Only values of PP > 0.70 and BS > 50 are shown, with values of PP > 0.95 and BS > 90 indicated with a circle. Nodes not recovered on the RAxML tree are indicated with a hyphen. Sequences generated in this study are highlighted in violet.

There are 131 records of Alcyonacea at > 3000 m depth in the CCZ, only eight of those representing preserved specimens (OBIS 2022). We collected three specimens belonging to three different species, only one assigned to a previously described species. Genetic sequences for both 16S and COI genes were amplified for each specimen, and included in a concatenated alignment (16S, COI, mtMutS, NADH2) used to generate a phylogenetic tree of Octocorallia (Fig. 15). Classification of Alcyonacea specimens from seabed imagery is often constrained by the lack of visibility of polyp morphology, particularly when these are small (e.g., family Primnoidae). Therefore, classification from in situ images is mostly based on broader features like the branching pattern, the length of the main stem, and/or the number, size, and positioning of polyps on branch nodes.

**Suborder Calcaxonia Grasshoff, 1999**

**Family Chrysogorgiidae Verrill, 1883**

#### Genus *Chrysogorgia* Duchassaing & Michelotti, 1864

##### ﻿ Chrysogorgia

Taxon classificationAnimaliaAlcyonaceaChrysogorgiidae 

sp. CCZ_112

874A146A-D57C-5D29-9A67-724D891A5A04

Fig. 16

###### Material.

Clarion-Clipperton Zone • 1 specimen; APEI 4; 7.2874°N, 149.8578°W; 4125 m deep; 04 Jun. 2018; Smith & Durden leg.; GenBank: ON400711 (COI), ON406602 (16S); NHMUK; Voucher code: CCZ_112.

###### Description.

**Figure 16.**
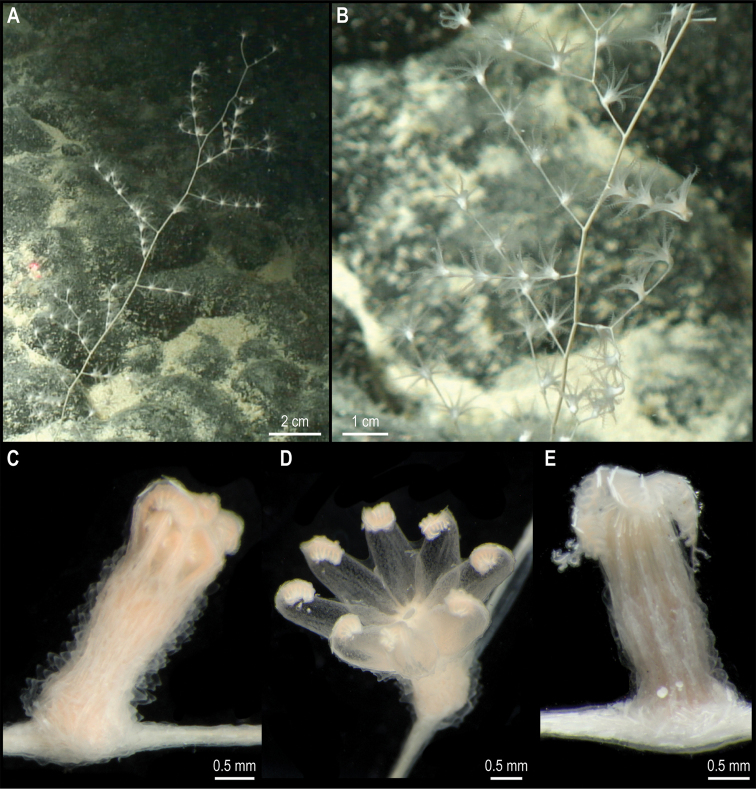
*Chrysogorgia* sp. CCZ_112 **A, B** in situ images of colony **C** closed polyp on live specimen **D** opened polyp on live specimen showing light orange colouration **E** closed polyp of preserved specimen. Scale bars: 2 cm (**A**); 0.5 mm (**C–E**). Image attribution: Durden and Smith (**A, B**); Wiklund, Durden, Drennan, and McQuaid (**C, D**); Bribiesca-Contreras (**E**).

Wide, long, sparsely branched colony, ~ 30 cm tall from the base (Fig. 16A, B). Polyps constricted basally on the neck (Fig. 16C–E), placed on internodes and absent from the main stem (Fig. 16A, B). Polyps are light orange when alive (Fig. 16C, D) and white after preservation (Fig. 16E). Sclerites near the polyp base are scale-like, but throughout the body and along the tentacle rachis are all elongate flat rods; sclerites are absent from the tentacle pinnules.

###### Remarks.

The sequence for the COI gene is 0% divergent from a sequence of a specimen of *Chrysogorgiaabludo* Pante & Watling, 2011 (specimen NAS102-3, GenBank accession number GQ180138) collected at Nashville Seamount, New England Seamounts at 2246 m depth (Station 102; 34.5828°N, 56.8433°W) included as comparative material during the species description (Pante and Watling 2011). In octocorals, it has been found that COI evolves very slowly and therefore it is not suitable for species discrimination, with different species having the same haplotype (McFadden et al. 2011). *Chrysogorgiaabludo* is distributed in the Atlantic Ocean, and morphological characters of the specimen collected in this study differ from the original description of *C.abludo*, as well as other species within the genus and hence considered a potentially new species In the phylogenetic tree (Fig. 15) the specimen was also recovered along with another specimen of *Chrysogorgia*, supporting its placement within the genus.

###### Comparison with image-based catalogue.

No similar Alcyonacea morphotypes have been catalogued so far from seabed imagery in the eastern CCZ or in abyssal areas of the Kiribati EEZ. Consequently, the in situ image of *Chrysogorgia* sp. CCZ_112 was catalogued as a new morphotype (i.e., *Chrysogorgia* sp. indet., ALC_017).

###### Ecology.

The specimen was attached to polymetallic crust on the slope of a seamount in the APEI 4, at 4124 m depth.

**Family Mopseidae Gray, 1870**

**Mopseidae sp. CCZ_088**

Fig. 17

**Material.** Clarion-Clipperton Zone • 1 specimen; APEI 4; 7.0089°N, 149.9109°W; 5018 m deep; 02 Jun. 2018; Smith & Durden leg.; GenBank: ON400705 (COI), ON406603 (16S); NHMUK XXX; Voucher code: CCZ_088.

**Figure 17.**
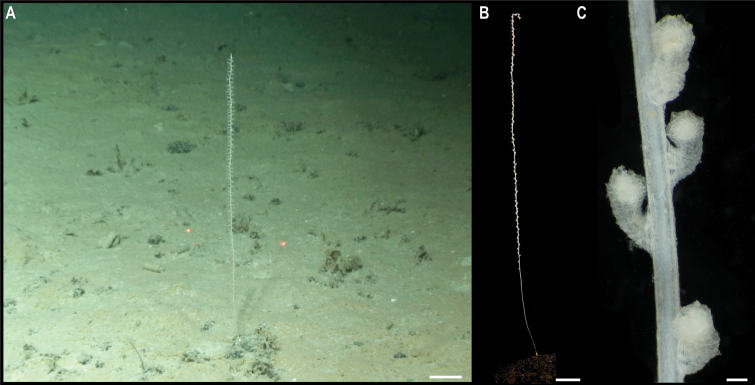
Mopseidae sp. CCZ_088 **A** in situ image **B** whole colony attached to a nodule **C** detail of polyps before preservation. Scale bars: 5 cm (**A**); 2 cm (**B**); 5 mm (**C**). Image attribution: Durden and Smith (**A**); Wiklund, Durden, Drennan, and McQuaid (**B, C**).

**Description.** Single specimen, with white axis and polyps; polyps standing perpendicular to the axis when alive (Fig. 17A). Colony is long, ~ 45 cm tall, and unbranched (Fig. 17A, B). Polyps are tall, ~ 2 mm, clavate, and standing parallel to the branch (Fig. 17C).

**Remarks.** Both 16S (0.3% K2P) and COI (0.6% K2) sequences are very similar to Mopseinae sp. NHM_330 (Dahlgren et al. 2016), which morphologically resembles the genus *Primnoisis*. The specimen from the western CCZ likely belongs to the same genus but based on genetic and morphological differences represents a different species from that of the eastern CCZ.

**Ecology.** The specimen was found attached to a nodule in abyssal sediments of APEI 4 at 5018 m depth.

**Comparison with image-based catalogue.** No similar Alcyonacea morphotypes had been catalogued so far from seabed imagery in the eastern CCZ or in abyssal areas of the Kiribati EEZ. Consequently, the in situ image of CCZ_088 was catalogued as a new morphotype (i.e., Mopseidae gen. indet., ALC_018). However, it is often not possible to determine whether such small and abundant polyps are arranged in pairs or not, or the actual orientation of these with regards to the axis from seabed images.

**Family Primnoidae Milne Edwards, 1857**

#### Genus *Calyptrophora* Gray, 1866

##### ﻿ Calyptrophora distolos

Taxon classificationAnimaliaAlcyonaceaPrimnoidae

Cairns, 2018

4F0C8664-E74B-57D0-B692-C90D9AE9FBC9

Fig. 18

###### Material.

Clarion-Clipperton Zone • 1 specimen; APEI 4; 7.2874°N, 149.8578°W; 4125 m deep; 04 Jun. 2018; Smith & Durden leg.; GenBank: ON400712 (COI), ON406604 (16S); USNM 1550968; Voucher: CCZ_131.

###### Description.

**Figure 18.**
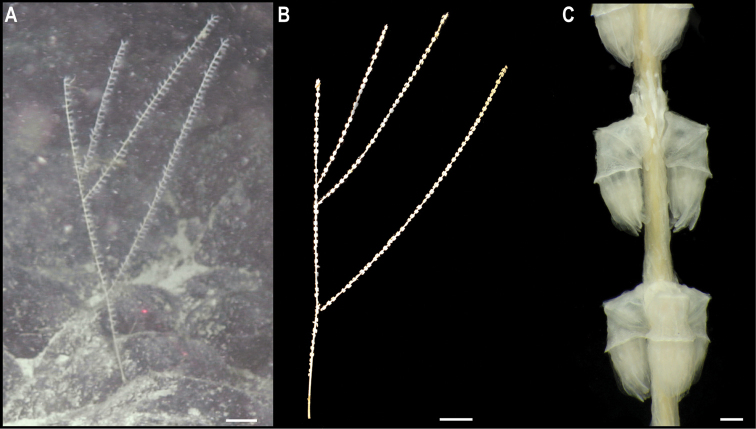
*Calyptrophoradistolos* Cairns, 2018. Specimen CCZ_132: **A** in situ image **B** whole colony **C** detail of polyps before preservation. Scale bars: 2 cm (**A, B**); 5 mm (**C**). Image attribution: Durden and Smith (**A**); Wiklund, Durden, Drennan, and McQuaid (**B, C**).

Branching uniplanar, colony ~ 20.8 cm tall, with polyps perpendicular to the stem in in situ images (Fig. 18A). Downward-oriented polyps, arranged parallel to the branch, mostly paired, but a few whorls with three to four polyps are present; polyps are ~ 2.7 mm tall and with an operculum longer than either of the body wall scales (Fig. 18B, C).

###### Remarks.

Morphological characters are concordant with the description of *Calyptrophoradistolos* (Cairns 2018). In addition to the paired polyps mentioned in the species description, this specimen also presents a few whorls with three or four polyps (Fig. 18C). Polyps are downward-oriented, therefore belonging to the *wyvillei* complex (Cairns 2018). The species is most similar to *C.persephone* Cairns, 2015, which has been described for the UK-1 and BGR areas in the CCZ (Cairns 2015). However, *C.persephone* is characterised as having polyps oriented upwards, therefore belonging to the *japonica* complex, and that are consistently arranged in whorls of three or four, with each basal scale bearing two prominent distal spines. *Calyptrophoradistolos* was described from the Enigma Seamount, south of Guam, at 3737 m depth, and has also been recorded for American Samoa at 2994 m depth (Cairns 2018). There are no genetic sequences available for other specimens of *C.distolos*, but the sequences generated here cluster with other species of the genus (Fig. 15). However, the genus was not recovered as monophyletic.

###### Ecology.

The specimen was found attached to a polymetallic crust on the slope of a seamount on APEI 4, at 4124 m depth.

###### Comparison with image-based catalogue.

A similar primnoid morphotype (i.e., *Calyptrophoradistolos* sp. inc., ALC_016) was catalogued from seabed imagery (also collected on a seamount) in the eastern CCZ (e.g., Cuvelier et al. 2020), but not in abyssal areas of the Kiribati EEZ.

**Order Pennatulacea Verrill, 1865**

A total of 79 records of Pennatulacea occurring at > 3000 m depth in the CCZ have been recorded in OBIS, but none represent preserved specimens (OBIS 2022). We recovered a single specimen, for which sequences of both 16S and COI genes were obtained, and which were included in the phylogenetic analysis of the Octocorallia (Fig. 15).

**Suborder Sessiliflorae Kükenthal, 1915**

**Family Protoptilidae Kölliker, 1872**

#### Genus *Protoptilum* Kölliker, 1872

##### ﻿ Protoptilum

Taxon classificationAnimaliaPennatulaceaProtoptilidae

stet. CCZ_068

615CA680-9C26-5169-BA0C-5CD7E7BE977E

Fig. 19

###### Material.

Clarion-Clipperton Zone • 1 specimen; APEI 7; 4.8897°N, 141.75°W; 3096 m deep; 27 May. 2018; Smith & Durden leg.; GenBank: ON400694 (COI), ON406605 (16S); NHMUK 2021.24; Voucher: CCZ_068

###### Description.

**Figure 19.**
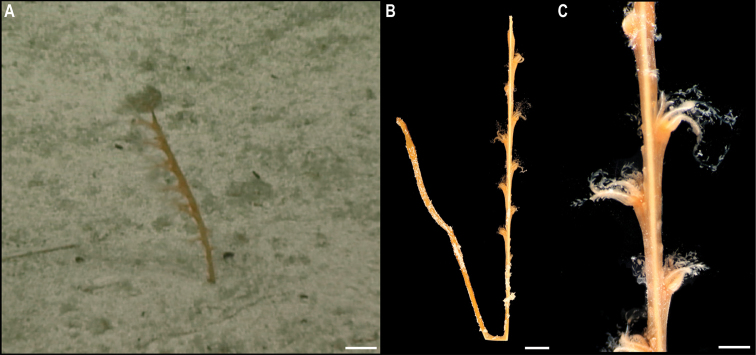
*Protoptilum* stet. CCZ_068 **A** in situ image **B** whole colony **C** detail of polyps before preservation. Scale bars: 1 cm (**A**); 5 mm (**B**); 2 mm (**C**). Image attribution: Durden and Smith (**A**); Wiklund, Durden, Drennan, and McQuaid (**B, C**).

Single specimen, ~ 12 cm tall, narrow sea pen; in situ colouration orange with whitish polyps (Fig. 19A). Two rows, opposite to each other, of elongated polyp calyces along the rachis (Fig. 19B, C).

###### Remarks.

The COI sequence forms a clade with sequences from *Protoptilum* (< 1% genetic divergence), a genus within the family Protoptilidae, while the 16S sequence is very similar to sequences of *Protoptilum* and *Distichoptilum*, both genera within the same family. In the phylogenetic tree, the family Protoptilidae was not recovered as monophyletic, but the CCZ specimen was recovered (with 1.00 posterior probability) as sister to *Protoptilumcarpenterii* Kölliker, 1872.

###### Ecology.

The specimen was found anchored to soft sediment on a seamount of APEI 7, at 3096 m depth.

###### Comparison with image-based catalogue.

No similar Pennatulacea morphotypes have been catalogued so far from seabed imagery in the eastern CCZ or in abyssal areas of the Kiribati EEZ. Consequently, the in situ image of *Protoptilum* stet. CCZ_068 was catalogued as a new morphotype (i.e., *Protoptilum* sp. indet., PEN_024). In seabed images, PEN_024 can resemble other single-branched sea pens or even soft corals.

**Subclass Ceriantharia Perrier, 1893**

**Order Spirularia den Hartog, 1977**

**Figure 20.**
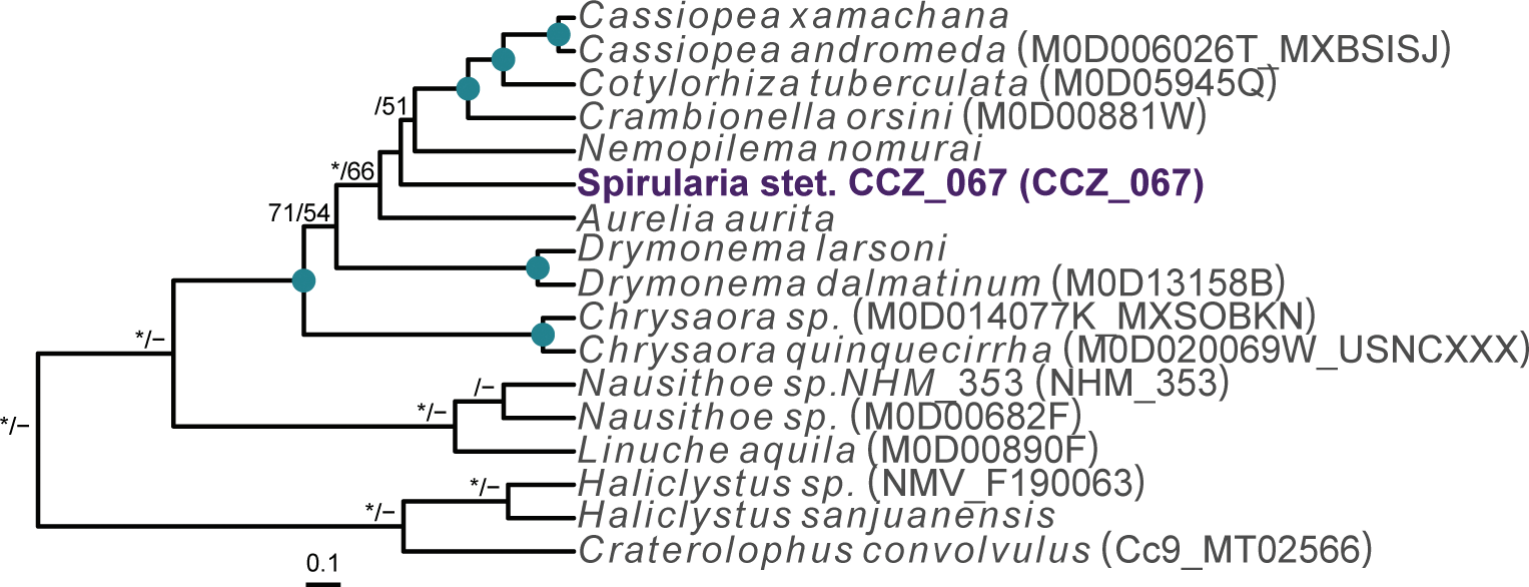
Rooted Bayesian phylogeny of Ceriantharia. Concatenated (12S, 16S, 18S, 28S, and COI) median consensus BEAST tree with posterior probability (PP) and bootstrap (BS) values indicated. Only values of PP > 0.70 and BS > 50 are shown, with values of PP > 0.95 and BS > 90 indicated with a circle. Nodes not recovered on the RAxML tree are indicated with a hyphen. Sequences generated in this study are highlighted in violet.

To date, there are no records from a minimum of 3000 m depth in the CCZ for the order Spirularia (OBIS 2022). We recovered a single specimen, for which the COI and 16S genes were successfully amplified and included in a concatenated matrix (12S, 16S, 18S, 28S, and COI) to estimate a phylogenetic tree of the Ceriantharia (Fig. 20).

**Spirularia stet. CCZ_067**

Fig. 21

**Material.** Clarion-Clipperton Zone • 1 specimen; APEI 7; 4.8875°N, 141.7572°W; 3132 m deep; 27 May. 2018; Smith & Durden leg.; GenBank: ON400693 (COI), ON406606 (16S); NHMUK 2021.23; Voucher: CCZ_067.

**Figure 21.**
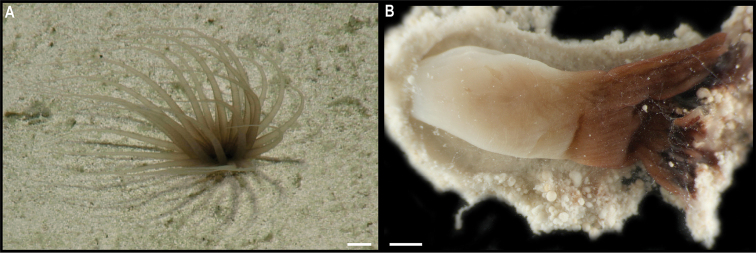
Spirularia stet. CCZ_067 **A** in situ image **B** specimen before preservation. Scale bars: 1 cm (**A**); 2 mm (**B**). Image attribution: Durden and Smith (**A**); Wiklund, Durden, Drennan, and McQuaid (**B**).

**Description.** Single specimen, unattached, tube-dweller with tentacles extended above the sediment in situ (Fig. 21A). Very long, conical, tapering, reddish brown tentacles; capitulum whitish when alive (Fig. 21A). Column is 12 mm in height and 5 mm in width excluding tentacles (Fig. 21B). Tube consisting of soft sediment.

**Remarks.** The closest matches to the COI and 16S sequences were sequences from other members of the family Cerianthidae: *Pachycenrianthus*, *Cerianthus*, *Ceriantheromorphe*. However, in the concatenated phylogeny, it forms a clade with *Boctrunidifer* sp. 1 and *Ceriantheopsisamericanus*, belonging to the families Botrucnidiferidae and Cerianthidae, respectively (Fig. 20). As Forero-Mejia et al. (2019) recovered both families as non-monophyletic and a revision of these is suggested, we were unable to assign it to a family.

**Ecology.** The specimen was found buried in the sediment on a seamount in APEI 7, at 3132 m depth.

**Comparison with image-based catalogue.** A very similar Ceriantharia morphotype (i.e., *Spirularia* sp. indet., CER_001) has been commonly encountered in seabed image surveys conducted across the eastern CCZ, always found semi-buried with the tentacles extending above the sediment surface.

**Class Scyphozoa Goette, 1887**

**Figure 22.**
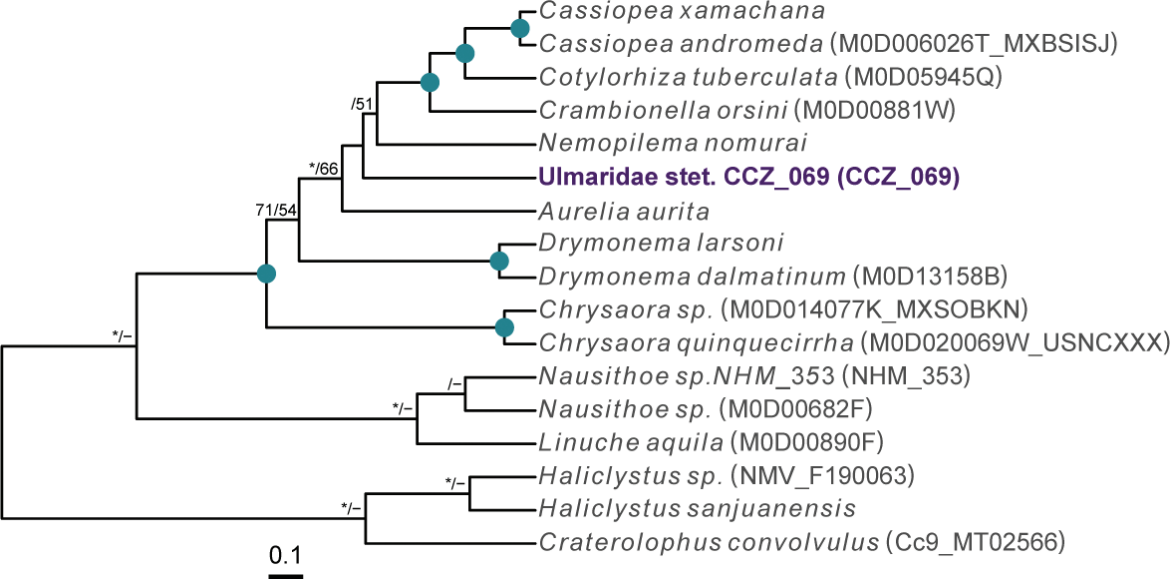
Rooted Bayesian phylogeny of Scyphozoa. Concatenated (16S, 18S, 28S, and COI) median consensus BEAST tree with posterior probability (PP) and bootstrap (BS) values indicated. Only values of PP > 0.70 and BS > 50 are shown, with values of PP > 0.95 and BS > 90 indicated with a circle. Nodes not recovered on the RAxML tree are indicated with a hyphen. Sequences generated in this study are highlighted in violet.

For the class Scyphozoa, there are currently 128 records from > 3000 m depth in the CCZ, but none represent preserved specimens (OBIS 2022). We collected a single specimen, for which the sequence for the COI gene was successfully amplified and included in a multi-gene phylogeny (16S, 18S, 28S, and COI) of the Scyphozoa (Fig. 22).

**Subclass Discomedusae Haeckel, 1880**

**Order Somaeostomeae Agassiz, 1862**

**Family Ulmaridae Haeckel, 1880**

**Ulmaridae stet. CCZ_069**

Fig. 23

**Material.** Clarion-Clipperton Zone • 1 specimen; APEI 7; 4.8876°N, 141.7572°W; 3133 m deep; 27 May. 2018; Smith & Durden leg.; GenBank: ON400695 (COI); NHMUK 2021.25; Voucher: CCZ_069.

**Figure 23.**
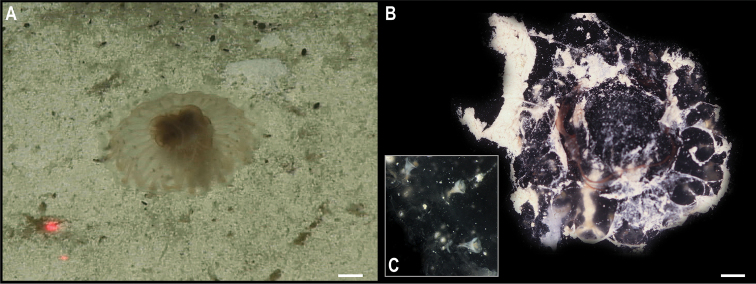
Ulmaridae stet. CCZ_069 **A** in situ image **B** specimen before preservation **C** rhopalia. Scale bars: 1 cm (**A**); 5 mm (**B**). Image attribution: Durden and Smith (**A**); Wiklund, Durden, Drennan, and McQuaid (**B, C**).

**Description.** Single specimen, ~ 4.5 cm in diameter; with transparent bell and light brown tentacles in situ (Fig. 23A). Rhopalia are evident around the bell (Fig. 23B, C).

**Remarks.** Only the sequence for the COI gene was successfully amplified, but none of the matches on public databases were informative. In the phylogenetic tree (Fig. 22), the CCZ specimen was recovered in a clade with other members of the class Discomedusae. As both the Rhizostomeae and Semaeostomeae were not well supported, the specimen was not confidently assigned to any of both orders based on COI only. However, the specimen morphologically resembles an undescribed ulmariid scyphozoan ( Somaeostomeae) that was observed in the New Britain Trench (Gallo et al. 2015).

**Ecology.** The specimen was found on the sediment of a seamount in APEI 7 at 3095–3132 m depth. A similar species of ulmariid from the New Britain Trench was found to skim the seafloor to feed on particulates on the sediment (Gallo et al. 2015).

**Comparison with image-based catalogue.** No similar Ulmaridae morphotypes have been catalogued so far from seabed imagery in the eastern CCZ or in abyssal areas of the Kiribati EEZ. Consequently, the in situ image of Ulmaridae stet. CCZ_069 was catalogued as a new morphotype (i.e., Ulmaridae gen. indet., SCY_010). A similarly shaped Ulmaridae morphotype (e.g., Ulmaridae gen. indet., SCY_009; opaque reddish bell, dark brown tentacles encircled with a white ring, and dark rhopalia around the bell), also eventually found crawling on the seabed surface, was previously catalogued from seabed imagery in nodule field areas of the eastern CCZ. When photographed lying on the seabed (as opposed to swimming in the water column), SCY_019 and SCY_010 may resemble an anemone, particularly in images collected at high altitude above the seabed (e.g., > 5 m).

**Phylum Echinodermata**

**Class Asteroidea de Blainville, 1830**

**Figure 24.**
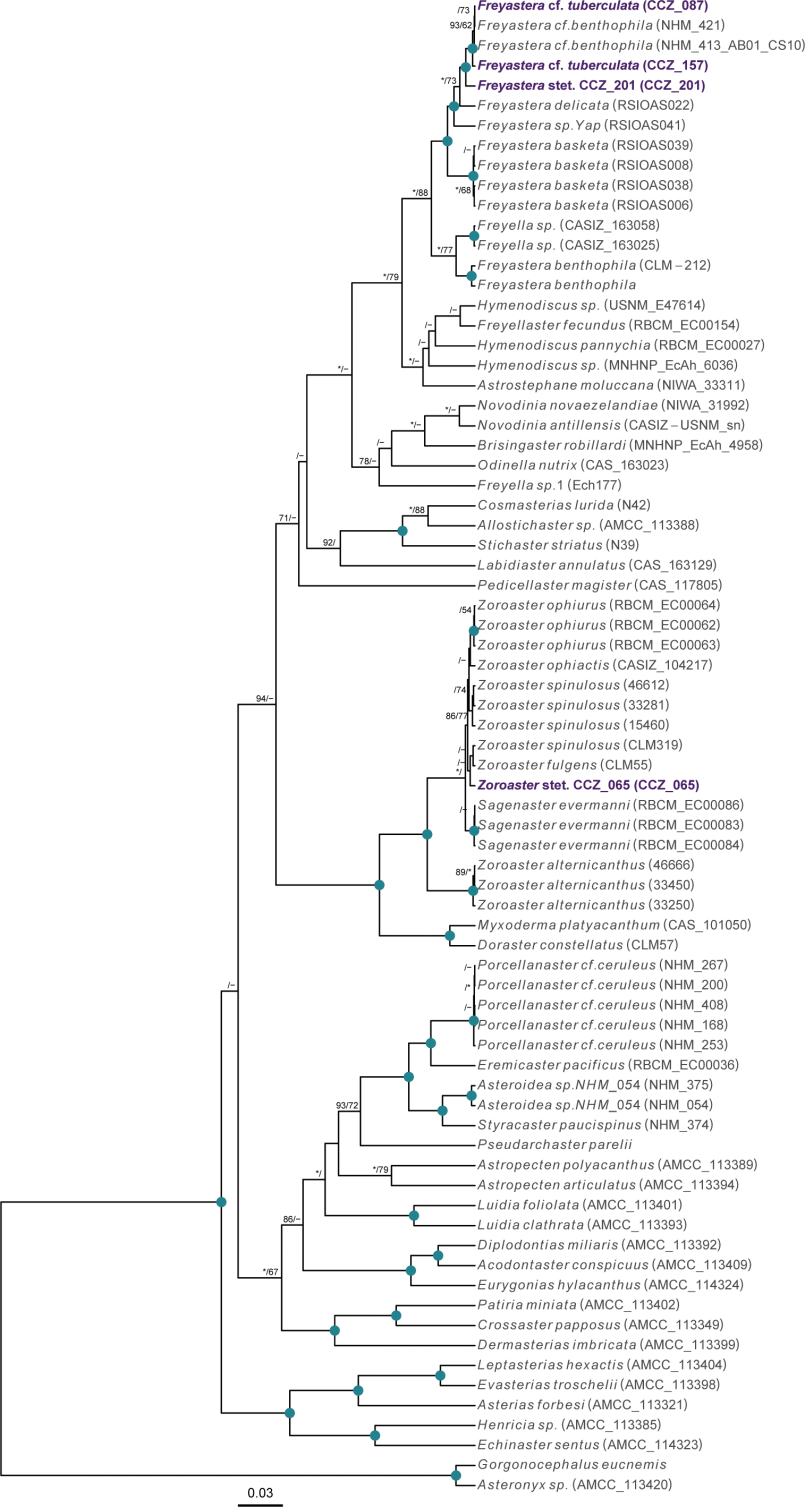
Rooted Bayesian phylogeny of Asteroidea. Concatenated (12S, 16S, 18S, COI, and H3) median consensus BEAST tree with posterior probability (PP) and bootstrap (BS) values indicated. Only values of PP > 0.70 and BS > 50 are shown, with values of PP > 0.95 and BS > 90 indicated with a circle. Nodes not recovered on the RAxML tree are indicated with a hyphen. Sequences generated in this study are highlighted in violet.

There are currently 245 records of sea stars occurring at a minimum of 3000 m depth in the CCZ, with only five of those representing preserved specimens (OBIS 2022). Four specimens were collected on the western CCZ, and sequences of the barcoding gene COI were generated for all of them, and 16S for a single specimen. These were included in a concatenated alignment of 12S, 16S, 18S, COI, and H3 used to estimate a phylogenetic tree (Fig. 24).

**Superorder Forcipulatacea Blake, 1987**

**Order Brisingida Fisher, 1928**

**Family Freyellidae Downey, 1986**

#### Genus *Freyastera* Downey, 1986

##### ﻿ Freyastera cf. tuberculata

Taxon classificationAnimaliaBrisingidaFreyellidae

(Sladen, 1889)

A7C19C55-8CF0-5ABD-B055-98C74F785B48

Fig. 25

###### Material.

Clarion-Clipperton Zone • 1 specimen; APEI 4; 6.9879°N, 149.9123°W; 5000 m deep; 02 Jun. 2018; Smith & Durden leg.; GenBank: ON400716 (COI); NHMUK 2022.80; Voucher code: CCZ_157 • 1 specimen; APEI 4; 6.9873°N, 149.9331°W; 5000 m deep; 06 Jun. 2018; Smith & Durden leg.; GenBank: ON400704 (COI); NHMUK 2022.79; Voucher code: CCZ_087.

###### Comparative material.

Pacific Ocean • 1 specimen, holotype of *Freyellabenthophila* Sladen, 1889; mid-South Pacific; 39.6833°S, 131.3833°W; 4663 m deep; Challenger Expedition, Stn. 289; NHMUK 1890.5.7.1078. Atlantic Ocean • 1 specimen, syntype of *Freyellatuberculata* Sladen, 1889; between west coast of Africa and Ascencion Islands; 22.3°N, 22.0333°W; 4389 m deep; Challenger Expedition, Stn. 346; NHMUK 1890.5.7.1077. • 1 specimen, syntype of *Freyellatuberculata*; between Canary Islands and Cape Verde Islands; 2.7° S, 14.6833°W; 4298 m deep; Challenger Expedition, Stn. 89; NHMUK 1890.5.7.1076.

###### Description.

**Figure 25.**
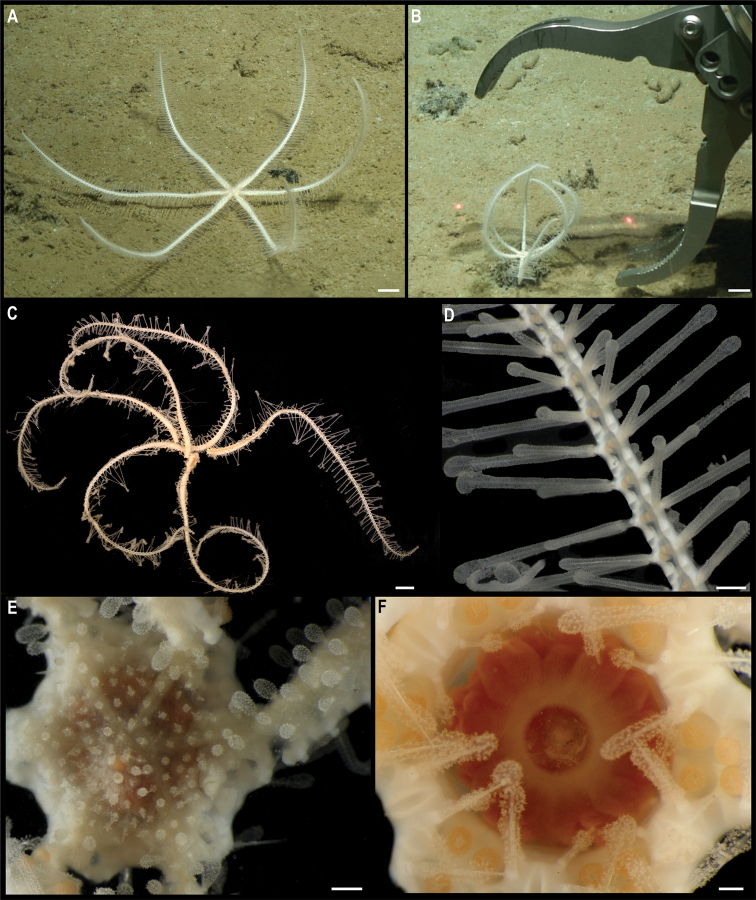
Freyasteracf.tuberculata (Sladen, 1889). Specimen CCZ_175: **A** in situ image **C** whole specimen **D** ventral surface of the arms before preservation. Specimen CCZ_087: **B** in situ image **E** details of dorsal disc **F** ventral disc surface before preservation. Scale bars: 2 cm (**A, B**); 1 cm (**C**); 2 mm (**D**); 1 mm (**E**); 0.5 mm (**F**). Image attribution: Durden and Smith (**A, B**); Wiklund, Durden, Drennan, and McQuaid (**C–F**).

Two specimens (R = 106 mm, r = 3 mm; R = 164 mm, r = 6 mm); live specimens whitish on both actinal and abactinal surfaces, tube feet transparent with bright orange flattened discs (Fig. 25A, B). Disc is small, somewhat rounded, slightly orange on actinal and abactinal surfaces (Fig. 25E, F); with six long, slender arms (Fig. 25A–C); lacking furrow spines (Fig. 25D). Each abactinal plate on the genital area bears a single spinelet (Fig. 25E), covered with a membrane with pedicellariae. Each mouth plate has two oral spines covered by a clear membrane bearing pedicellariae (Fig. 25F); one located on the adoral margin of the mouth plate and the suboral spine located above the centre of the mouth plate.

###### Remarks.

The COI sequences were very similar to sequences of Freyasteracf.benthophila (Sladen, 1889) collected in the UK-1 contract area from the CCZ (Glover et al. 2016a), and which were recovered in a single clade (Fig. 24). Only arm segments were recovered from the UK-1 specimens, and although they were found to resemble *F.benthophila*, the whole specimens collected in the western CCZ differ from the original description for the species. Only five species are known for having six rays: *F.sexradiata* (Perrier, 1885), *F.benthophila*, *F.tuberculata* (Sladen, 1889), *F.basketa*Zhang et al., 2019, and *F.delicata*Zhang et al., 2019. However, *F.benthophila* is easily distinguished from the other two species by its abactinal armament; each abactinal plate bearing two or three spinelets covered with a simple membrane with no pedicellariae (Sladen 1889). The specimens from the CCZ have abactinal spinelets covered by a membrane that bears pedicellariae (Fig. 25E). They also differ from *F.benthophila* in having the spines on the adoral margin of the mouth-plates covered by a clear membrane bearing pedicellariae instead of an opaque membrane with no pedicellariae (Downey 1986). In addition, the suboral spines are located above the centre of the mouth plate (Fig. 25F), as in *F.tuberculata*, and not below the centre of the mouth plate as described for *F.benthophila* (Downey 1986). Syntypes from *F.tuberculata* are from the Atlantic Ocean (Sladen 1889), but it has been reported for the Eastern Tropical Pacific (0.05°N, 117.25°W) at 4243 m depth (Downey 1986). Unfortunately, there are no genetic sequences available for this species, but COI sequences from CCZ specimens are highly divergent from the sequence of *F.benthophila* collected in the Mariana Trench (K2P distance: 13–14%). In the phylogenetic tree they are also recovered in different clades, very close to another species reported herein, and to *F.delicata* and *Freyastera* sp. Yap (in Zhang et al. 2019) (Fig. 24). The specimen collected here represents the same species as found in the eastern CCZ, Freyasteracf.benthophila (Glover et al. 2016b).

###### Ecology.

One specimen was observed on the sedimented seafloor (CCZ_157), while another was sitting on a nodule with the actinal surface against the muddy seafloor and lifting the tip of the arms like a basket (CCZ_087). Both seastars were collected on abyssal sediments of APEI 4 at 5000 m depth. During morphological examination of these samples, the exoskeleton of a large (> 6 mm long), digested copepod was found in the stomach of specimen CCZ_157.

##### ﻿ Freyastera

Taxon classificationAnimaliaBrisingidaFreyellidae

stet. CCZ_201

7C871719-3230-51E2-B0B9-D8B017C0B3E7

Fig. 26

###### Material.

Clarion-Clipperton Zone • 1 specimen; APEI 1; 11.2518°N, 153.6059°W; 5204 m deep; 10 Jun. 2018; Smith & Durden leg.; GenBank: ON400730 (COI); NHMUK 2022.81; Voucher code: CCZ_201.

###### Comparative material.

Pacific Ocean • 1 specimen, holotype of *Freyellabenthophila* Sladen, 1889; mid-South Pacific; 39.6833°S, 131.3833°W; 4663 m deep; Challenger Expedition, Stn. 289; NHMUK 1890.5.7.1078. Atlantic Ocean • 1 specimen, syntype of *Freyellatuberculata* Sladen, 1889; between west coast of Africa and Ascencion Islands; 22.3°N, 22.0333°W; 4389 m deep; Challenger Expedition, Stn. 346; NHMUK 1890.5.7.1077. • 1 specimen, syntype of *Freyellatuberculata*; between Canary Islands and Cape Verde Islands; 2.7°S, 14.6833°W; 4298 m deep; Challenger Expedition, Stn. 89; NHMUK 1890.5.7.1076.

###### Description.

**Figure 26.**
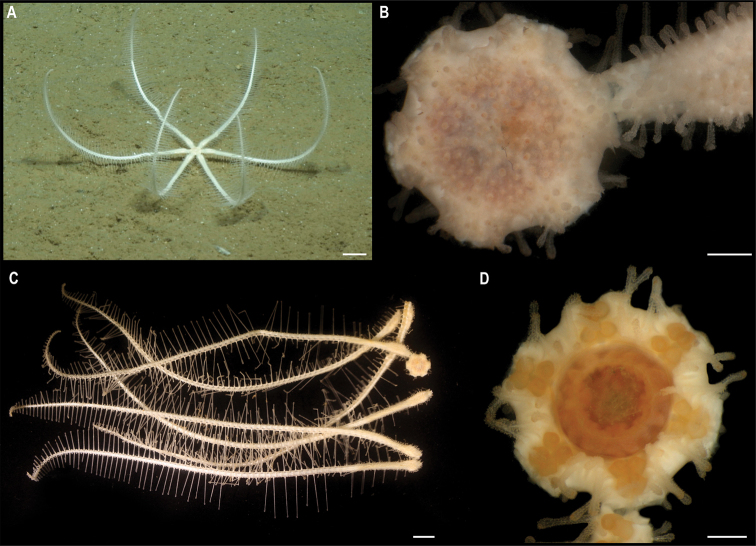
*Freyastera* stet. CCZ_201 **A** in situ image **B** whole specimen **C** dorsal disc surface **D** ventral disc surface. Scale bars: 2 cm (**A**); 2 mm (**B**); 1 cm (**C**); 2 mm (**D**). Image attribution: Durden and Smith (**A**); Wiklund, Durden, Drennan, and McQuaid (**B–D**).

Single specimen, with very small disc and six long, slender, tapered arms (R = 190 mm, r = 5 mm; Fig. 26A). Specimen before preservation has a slightly orange adoral disc surface, white arms, and bright orange tube feet discs (Fig. 26A–D). Disc is somewhat rounded, covered with short, scattered spines covered by a membrane bearing pedicellariae (Fig. 26B). Arms with long, slender lateral spines, also covered with a membrane bearing pedicellariae (Fig. 26C). Each abactinal plate on the genital area bears a one to few spinelets, completely covered with a membrane with pedicellariae. Each mouth plate has two oral spines covered by a clear membrane bearing pedicellariae; one located on the adoral margin of the mouth plate and the suboral spine located above the centre of the mouth plate.

###### Remarks.

The COI sequence is 4% divergent from the two specimens of Freyasteracf.tuberculata reported herein, and hence considered a separate species. It is also divergent (> 4% K2P distance) to sequences of other species of *Freyastera*, but forms a monophyletic clade with those, confirming its placement within the genus (Fig. 24). Morphologically it resembles Freyasteracf.tuberculata, but differ in having slightly shorter and more scattered spinelets on the abactinal surface of the disc. Also, the spinelets on the abactinal plates on the genital area are more numerous, and completely covered by a membrane bearing pedicellariae, instead of having a membrane that does not cover the spine all the way down to the base as in F.cf.tuberculata. In addition, the genetic distance with specimens of that species corresponds to the genetic distance between morphologically distinct species and hence considered a separate species.

###### Ecology.

The specimen was collected on the sedimented abyssal plain of APEI 1 at 5204 m depth, with arms curled up like a basket (Fig. 26A).

###### Comparison with image-based catalogue.

*Freyastera* spp. are commonly found in image-based megafauna assessments across the CCZ (e.g., Amon et al. 2016; Amon et al. 2017b), abyssal areas of the Kiribati EEZ, and other areas of the Pacific abyss (e.g., Peru Basin: Simon-Lledó et al. 2019a), both in nodule fields and in seamount areas. The relatively large size of adult specimens facilitates the detection of these brisingids even upon imagery collected at high altitudes (> 5 m) above the seabed. However, only one *Freyastera* sp. morphotype (e.g., *Freyastera* sp. indet., AST_002) has been catalogued so far, as differences in structure of the abactinal armament and/or the suboral spines are not visible from seabed images.

**Order Forcipulatida Perrier, 1884**

**Family Zoroasteridae Sladen, 1889**

#### Genus *Zoroaster* Wyville Thomson, 1873

##### ﻿ Zoroaster

Taxon classificationAnimaliaForcipulatidaZoroasteridae

stet. CCZ_065

B306AD78-526D-5D6F-912E-2D5CAD3DDF6A

Fig. 27

###### Material.

Clarion-Clipperton Zone • 1 specimen; APEI 7; 4.8877°N, 141.7569°W; 3132 m deep; 27 May. 2018; Smith & Durden leg.; GenBank: ON400691 (COI), ON406607 (16S); NHMUK 2022.78; Voucher code: CCZ_065.

###### Description.

**Figure 27.**
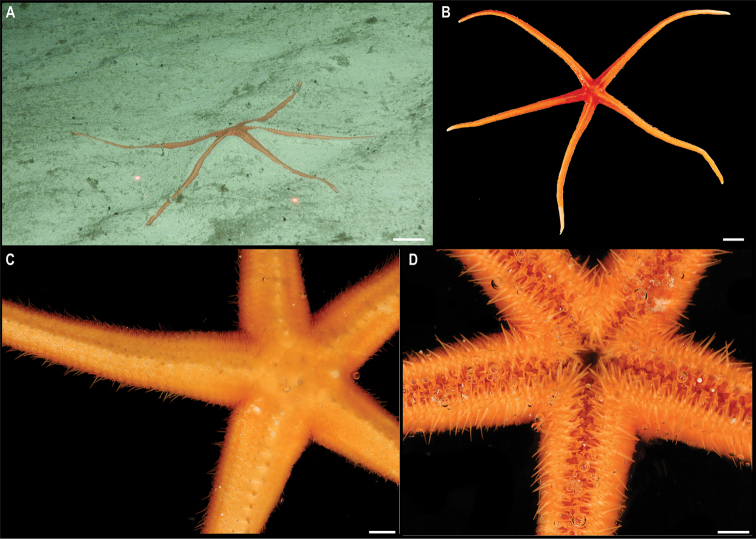
*Zoroaster* stet. CCZ_065 **A** in situ image **B** abactinal view of whole specimen **C** detail of abactinal surface **D** detail of actinal surface before preservation. Scale bars: 3 cm (**A**); 2 cm (**B**); 5 mm (**C, D**). Image attribution: Durden and Smith (**A**); Wiklund, Durden, Drennan, and McQuaid (**B–D**).

Single specimen (R = 16.6 cm, r = 1.3 cm). Actinal and abactinal surfaces are bright orange when alive, with ambulacrum slightly darker orange (Fig. 27A–D). Small disc; with five long, slender arms, gradually tapering distally (Fig. 27B). Carinal plates bear conical primary spines, forming a single longitudinal row that runs along the arm (Fig. 27A, C).

###### Remarks.

Morphological characters are concordant with the description of the genus *Zoroaster*. The phylogenetic analyses also recovered the specimen in a well-supported clade with other species of the genus (Fig. 24). However, COI divergence between species in the genus is very low (K2P distance ≈ 1–2%) and species-level clades were not recovered in the phylogeny, preventing us from assigning it to any species based on COI sequences. The closest match to the COI sequence of the CCZ specimen is a sequence from the long-armed morphotype (K2P distance 0.6%, GenBank accession number AY225785.1) identified for *Z.fulgens* Wyville Thomson, 1873 in the Porcupine Seabight, Atlantic Ocean (50.1987°N, 14.6593°W; 4001 m depth; Howell et al. 2004). Although this value is concordant with intraspecific divergence in the genus, the 16S sequences divergence between the CCZ specimen and the long-armed morphotype (K2P 1.1%) is larger than between the long-armed morphotype and *Z.spinulosus* Fisher, 1906 (K2P 0.0%) and between *Z.spinulosus* and *Z.ophiactis* Fisher, 1916 (K2P 0.3%).

###### Ecology.

The specimen was found partially buried in the sediment on the seamount on APEI 7 at 3133 m depth.

###### Comparison with image-based catalogue.

No similar Zoroasteridae morphotypes have been catalogued so far from seabed imagery in the eastern CCZ nor in abyssal areas of the Kiribati EEZ. Consequently, the in situ image of Zoroaster stet. CCZ_065 was catalogued as a new morphotype (i.e., *Zoroaster* sp. indet., AST_025).

**Class Crinoidea**

**Figure 28.**
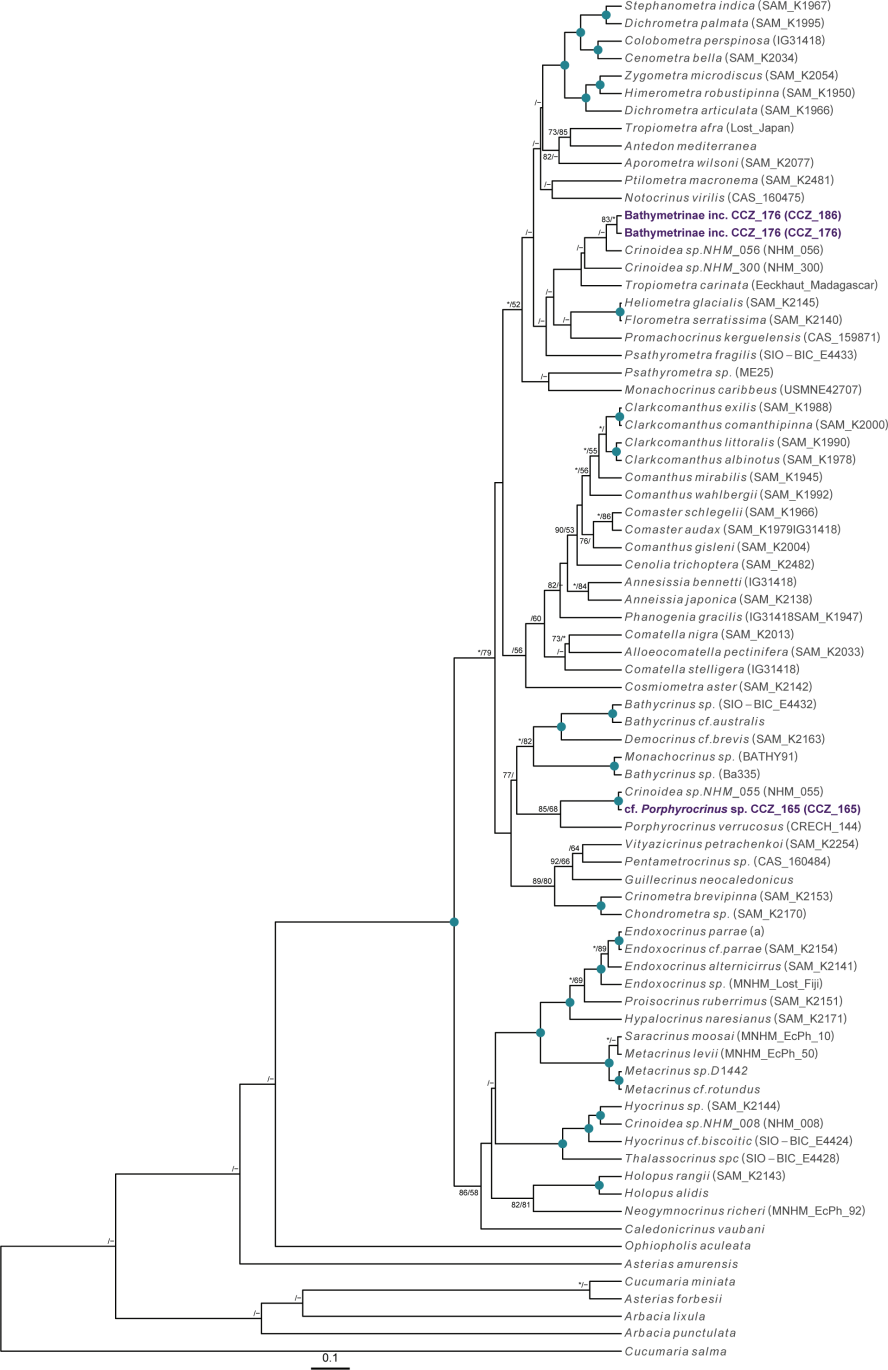
Rooted Bayesian phylogeny of Crinoidea. Concatenated (16S, 18S, 28S, COI, and CytB) median consensus BEAST tree with posterior probability (PP) and bootstrap (BS) values indicated. Only values of PP > 0.70 and BS > 50 are shown, with values of PP > 0.95 and BS > 90 indicated with a circle. Nodes not recovered on the RAxML tree are indicated with a hyphen. Sequences generated in this study are highlighted in violet.

To date, there are 66 records of crinoids occurring deeper than 3000 m in the CCZ, with only seven of these representing preserved specimens (OBIS 2022). Three specimens, belonging to two species, were collected in the western CCZ. The barcoding gene COI was amplified for all specimens, and sequences were included in a concatenated alignment (16S, 18S, 28S, COI, and CytB) used to estimate a phylogenetic tree for the class (Fig. 28).

**Subclass Articulata Zittel, 1879**

**Order Comatulida**

**Suborder Bourgueticrinina Sieverts-Doreck, 1953**

**Family Phrynocrinidae AH Clark, 1907**

**Subfamily Porphyrocrininae AM Clark, 1973**

#### Genus *Porphyrocrinus* Gislén, 1925

##### ﻿cf. Porphyrocrinus

Taxon classificationAnimaliaComatulidaPhrynocrinidae

sp. CCZ_165

0B801D6B-3673-5E0B-8261-B07B0B18B015

Fig. 29

###### Material.

Clarion-Clipperton Zone • 1 specimen; APEI 4; 6.9879°N, 149.9327°W; 5002 m deep; 06 Jun. 2018; Smith & Durden leg.; GenBank: ON400718 (COI), ON406616 (16S); NHMUK 2022.76; Voucher code: CCZ_165.

###### Description.

**Figure 29.**
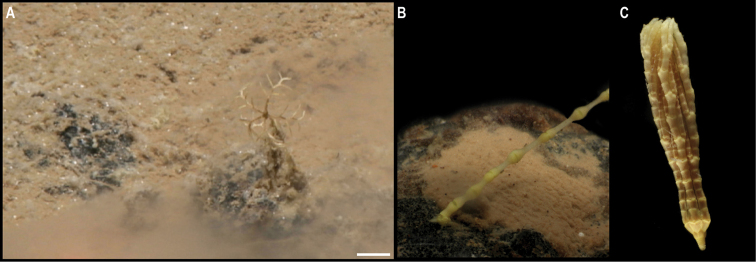
cf. sp. CCZ_165 **A** in situ image **B** xenomorph stalk attached to a polymetallic nodule **C** detached crown before preservation. Scale bars:1 cm (**A**). Image attribution: Durden and Smith (**A**); Wiklund, Durden, Drennan, and McQuaid (**B, C**).

Single specimen, attached to a nodule by a xenomorphic stalk (Fig. 29A). Crown (Fig. 29C) detached from stalk (Fig, 29B); L = 32 mm, composed of a crown and short proximal part of stalk. Proximal stalk composed of 5 very thin discoidal columnals up to 0.54 mm in diameter. Basal circlet truncated conical with distal diameter 0.54 mm and adoral diameter 0.78 mm; basals five, pentagonal in shape, with sunken lateral edges. Marked angle (~ 120°) between basals and radials. Radials five, pentagonal in shape; distal diameter 1.48 mm. Crown has five undivided arms. IBr1 are in close apposition with thin lateral flanges. Brachial formula 1+2 3+4 5+6 7+8 8+9 etc. First pinnule at IBr6; following pinnules every second ossicle; P1 has eight segments 4.04 mm in length; P2 is similar with eight pinnulars and 4.9 mm in length; P1 and P2 display lateral discoidal plates along ambulacral groove.

###### Remarks.

Morphological characters are concordant with those of the family Phrynocrinidae and the genus *Porphyrocrinus* as understood by Messing (2016). This is the first record of the genus in the Eastern Pacific. Only two specimens have been previously recorded from similar depths but collected from the Eastern Atlantic and attributed to Porphyrocrinuscf.incrassatus (Eléaume et al. 2012). In the phylogenetic tree the specimen is recovered in a monophyletic clade with other sequences from members of the family (Fig. 28) and represents a new species. Based on genetic divergence of the COI gene (0.5% K2P), the specimen found in the eastern CCZ (Crinoidea sp. NHM_055; Glover et al. 2016b) belongs to the same species. However, the specimen in Glover et al. (2016b) was not identified to family level or lower taxonomic level due to its early developmental stage, lacking key diagnostic morphological features.

###### Ecology.

The specimen was found attached to a nodule in the abyssal sediments of APEI 4 at 5001 m depth.

###### Comparison with image-based catalogue.

No similar Comatulida morphotypes have been catalogued so far from seabed imagery in the eastern CCZ or in abyssal areas of the Kiribati EEZ. Consequently, the in situ image of CCZ_065 was catalogued as a new morphotype (i.e., *Porphyrocrinus* sp. indet., CRI_008). Note however, that the in situ image of CCZ_065 was collected from an oblique angle and zoomed-in camera, generating a detailed view of a specimen that, owing to its small size, would be otherwise difficult to identify in quantitative assessments, e.g., where images are usually collected vertically-facing, fully zoomed out, and at a higher altitude above the seabed.

**Superfamily Antedonoidea Norman, 1865**

**Family Antedonidae Norman, 1865**

**Subfamily Bathymetrinae AH Clark, 1909**

**Bathymetrinae inc. CCZ_176**

Fig. 30

**Material.** Clarion-Clipperton Zone • 1 adult specimen; APEI 4; 6.9879°N, 149.9326°W; 5009 m deep; 06 Jun. 2018; Smith & Durden leg.; GenBank: ON400719 (COI), ON406617 (16S); NHMUK 2022.77; Voucher code: CCZ_176. • 1 specimen pentacrinoid stage; APEI 1; 11.2751°N, 153.7444°W; 5241 m deep; 09 Jun. 2018; Smith & Durden leg.; GenBank: ON400723 (COI), ON406618 (16S); NHMUK 2022.60; Voucher code: CCZ_186.

**Figure 30.**
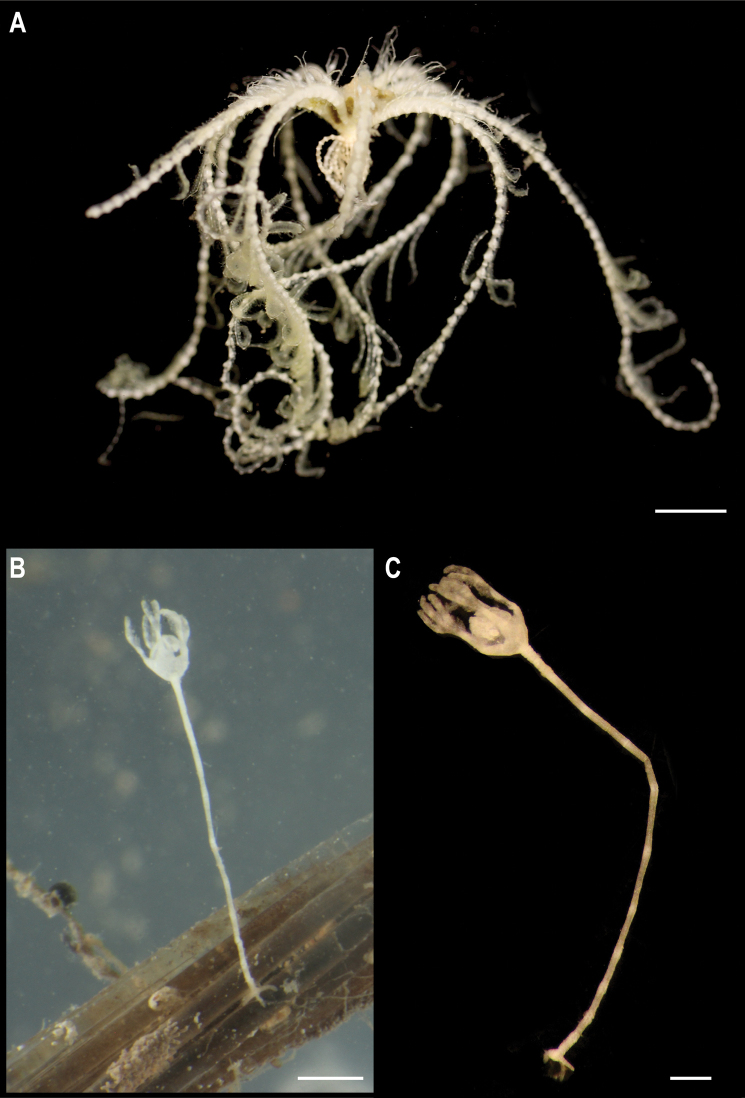
Bathymetrinae inc. CCZ_176 **A** side view of adult specimen. Specimen CCZ_186 **B** pentacrinoid stage attached to a glass sponge stalk **C** pentacrinoid stage. Scale bars: 5 mm (**A**); 1 mm (**B**); 0.5 mm (**C**). Image attribution: Wiklund, Durden, Drennan, and McQuaid (**A, B**); Bribiesca-Contreras 2019 (**C**).

**Description.** Two specimens, one adult (CCZ_176; Fig. 30A, B) and one pentacrinoid stage (CCZ_186; Fig. 30C, D), both whitish when alive and attached to a glass sponge stalk. Adult with 10 arms; centrodorsal low conical. Cirri ~ 17, length 7.5 mm; c1 W > L; c2 W = L; c3 longest L = 0.95 mm, W at centre of ossicle = 0,25, W distal = 0,45; following cirrals decreasing in length to c8 or c9; c3 to c17 with everted distal edge; c8 to c14 with a spine on distal edge; c17 slightly longer than wide; claw same length as c17; opposing spine small. No basal visible. Radial 5, visible, extending beyond the rim of centrodorsal. First brachitaxis of two ossicles well separated laterally. Ibr1 rectangular slightly incised by Ibr2; Ibr2 axillery, losangic. Subsequent brachials very long; syzygies at 3+4, 9+10. First pinnule P1 on br2, 14 segments very thin and slender, composed of very long segments starting at p3 with L < 6× W. In pentacrinoid stage, five arms are visible, with orals, and stalk.

**Remarks.** Morphological characters are concordant with those of the subfamily Bathymetrinae in the family Antedonidae. The closest match (2.7% K2P) to the COI sequences is a sequence of *Psathyrometrafragilis* (AH Clark, 1907) from Rodriguez Seamount (1887 m; SIO-BIC E4433), within the family Zenometridae. However, in the phylogenetic analysis the specimens were recovered in a different clade from *Psathyrometra* spp. (Fig. 28), but in a well-supported clade with two species (Crinoidea sp. NHM_056, Crinoidea sp. NHM_300) from the eastern CCZ (Glover et al. 2016b). The two species previously recorded in the eastern CCZ were delimited only from genetic sequences, as they seem to be early pentacrinoid stages and thus lack morphological characters for identification. However, based on the genetic divergence values with the species Bathymetrinae inc. CCZ_176 (~ 10% K2P), the two eastern CCZ species are most likely members of the subfamily Bathymetrinae.

**Ecology.** The adult specimen was found attached to a glass sponge stalk (Fig. 11A), along with an anemone, in abyssal sediments of APEI 4 at 5001 m depth. After careful examination of the material in the laboratory, a pentacrinoid stage was found attached to a sponge stalk (Figs 6A, 30B), along with the cirriped Catherinumcf.novaezelandiae (specimen CCZ_185) and the anemone Metridioidea stet. CCZ_164, in abyssal sediments of APEI 1 at 5241 m depth.

**Comparison with image-based catalogue.** A very similar Comatulida morphotype (i.e., Bathymetrinae gen. indet., CRI_001) has been commonly encountered in seabed image surveys conducted across the eastern CCZ, both in nodule fields and in seamount areas (Amon et al. 2017b). In contrast, CRI_001 was not encountered in image surveys conducted within abyssal areas of the Kiribati EEZ, where the presence of Crinoids was substantially lower than at the eastern CCZ (e.g., only nine specimens representing three morphotypes encountered in ~ 15,000 m^2^ of seabed surveyed; Simon-Lledó et al. 2019d).

**Class Echinoidea**

To date, there are 1455 records of echinoids occurring deeper than 3000 m in the CCZ, 11 of these representing preserved specimens (OBIS 2022). Two specimens belonging to different species were collected. Sequences for the barcoding gene COI were successfully amplified for both specimens and included in a COI-only phylogenetic tree.

**Figure 31.**
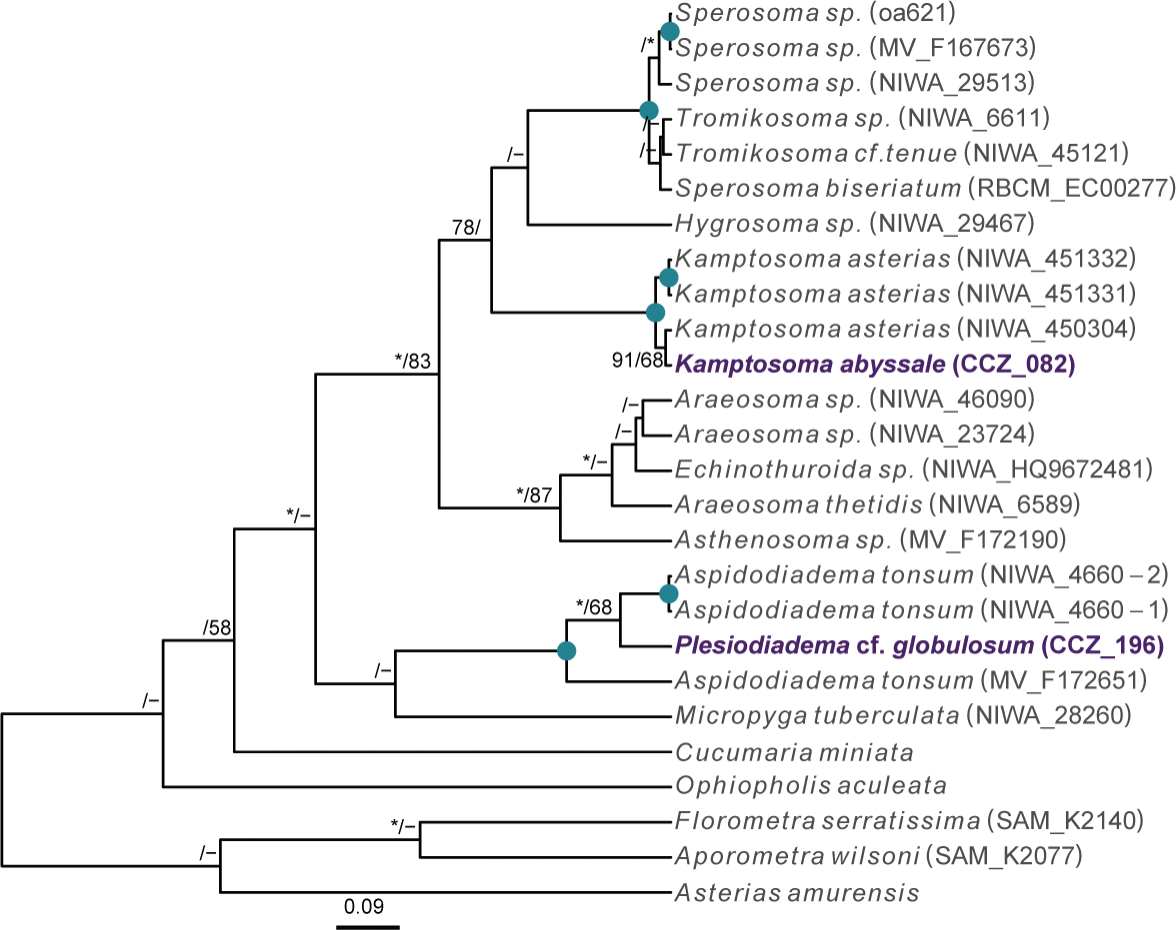
Phylogenetic tree of Echinoidea. COI-only median consensus BEAST tree with posterior probability (PP) and bootstrap (BS) values indicated. Only values of PP > 0.70 and BS > 50 are shown, with values of PP > 0.95 and BS > 90 indicated with a circle. Nodes not recovered on the RAxML tree are indicated with a hyphen. Sequences generated in this study are highlighted in violet.

**Subclass Euechinoidea Bronn, 1860**

**Infraclass Audolonta Jackson, 1912**

**Superoder Echinothuriacea Jensen, 1982**

**Order Aspidodiadematoida Kroh & Smith, 2010**

**Family Aspidodiadematidae Duncan, 1889**

#### Genus *Plesiodiadema* Pomel, 1883

##### ﻿ Plesiodiadema cf. globulosum

Taxon classificationAnimaliaAspidodiadematoidaAspidodiadematidae 

(A. Agassiz, 1898)

1691E484-09DF-56EB-B3F6-C9BE8857DFB0

Fig. 32

###### Material.

Clarion-Clipperton Zone • 1 specimen; APEI 1; 11.2527°N, 153.5848°W; 5204 m deep; 10 Jun. 2018; Smith & Durden leg.; GenBank: ON400726 (COI), ON406628 (18S); CASIZ 229305; Voucher code: CCZ_196.

###### Description.

**Figure 32.**
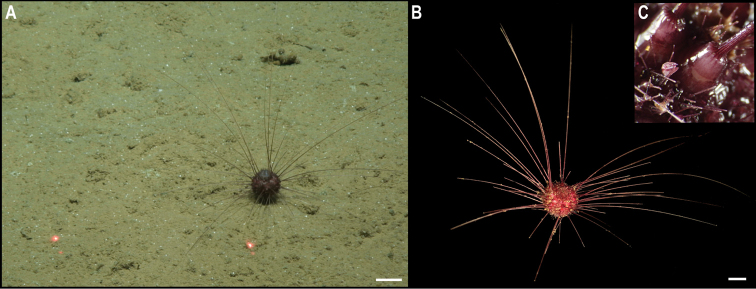
Plesiodiademacf.globulosum (A. Agassiz, 1898). Specimen CCZ_196: **A** in situ image **B** specimen after recovery **C** detail of pedicellaria of specimen CCZ_196. Scale bars: 2 cm (**A**); 1 cm (**B**). Image attribution: Durden and Smith (**A**); Wiklund, Durden, Drennan, and McQuaid (**B, C**).

Single specimen, with a somewhat spherical, slightly flattened test (d = 2 cm, H = 1.5 cm). In situ colouration is purple, but the inflated anal cone is greyish blue (Fig. 32A). Primary spines are also purple, very long (up to 17 cm), thin, flexible, and strongly verticillate (Fig. 32B, C). Pedicellariae are tridentate (Fig. 32C).

###### Remarks.

In 1980, the RV *Governor Ray* collected several Aspidodiadematidae specimens in the CCZ at ~ 4,800 m, and were assigned to the species *P.globulosum*. The type localities of *P.globulosum* are the north of Malpelo Island, and from off Galera Point, Ecuador in the Pacific Ocean, from 2877 to 3241 m depth (Agassiz 1898). There are no genetic sequences available on public databases for the genus, but both COI and 18S closest matches are to species of the genus *Aspidodiadema* A. Agassiz, 1879, within the same family (18S: 99.4% similar to *A.jacobyi* A. Agassiz, 1880). The COI-only tree recovered a monophyletic clade including three specimens of *A.tonsum* (Fig. 31), but the genetic divergence is within interspecific values for COI (6.5–11.7%). Despite morphological characters being in accordance with the diagnostic characters for *P.globulosum*, the specimen is listed as cf. as the collection site is much deeper than the type locality.

###### Ecology.

The specimen was collected on the sedimented abyssal plain of APEI 1, at 5203 m depth.

###### Comparison with image-based catalogue.

A very similar *Plesiodiadema* sp. morphotype (i.e., *Plesiodiademaglobulosum* sp. inc., URC_003) has been commonly found in image-based megafauna assessments conducted in the eastern CCZ (e.g., Amon et al. 2017b) and other areas of the eastern Pacific abyss (e.g., Yuzhmorgeologiya exploration area; Kamenskaya et al. 2013; Peru Basin; Simon-Lledó et al. 2019a), both in nodule fields and in seamount areas. URC_003 is usually the most abundant echinoid encountered in image-based megafauna surveys conducted at the eastern CCZ. In contrast, URC_003 was not encountered in surveys conducted in abyssal areas of the Kiribati EEZ, where kamptosomatids (e.g., see below) appeared to dominate the echinoid community (Simon-Lledó et al. 2019d).

**Order Echinothurioida Claus, 1880**

**Family Kamptosomatidae Mortensen, 1934**

#### Genus *Kamptosoma* Mortensen, 1903

##### ﻿ Kamptosoma abyssale

Taxon classificationAnimaliaEchinothurioidaKamptosomatidae

Mironov, 1971

5562C7A9-6CDF-514A-9DE0-48A3D4ACE624

Fig. 33

###### Material.

Clarion-Clipperton Zone • 1 specimen; APEI 4; 7.036°N, 149.9395°W; 5040 m deep; 01 Jun. 2018; Smith & Durden leg.; GenBank: ON400701 (COI); CASIZ 229306; Voucher code: CCZ_082.

###### Other material.

Pacific Ocean • 1 specimen, holotype of *Kamptosomaasterias* (A. Agassiz); off the coast of Chile; 33.5167°S, 74.7167°W; 3950 m deep; Challenger Expedition, Stn. 299; NHMUK 1881.11.22.114. • 2 specimens, *Kamptosomaabyssale* Mironov, 1971; Tasman Sea; 0°N, 0°E; 4850–4800 m deep; Galathea Expedition, Stn. 574; NHMUK 1984.1.25.86-87.

###### Description.

**Figure 33.**
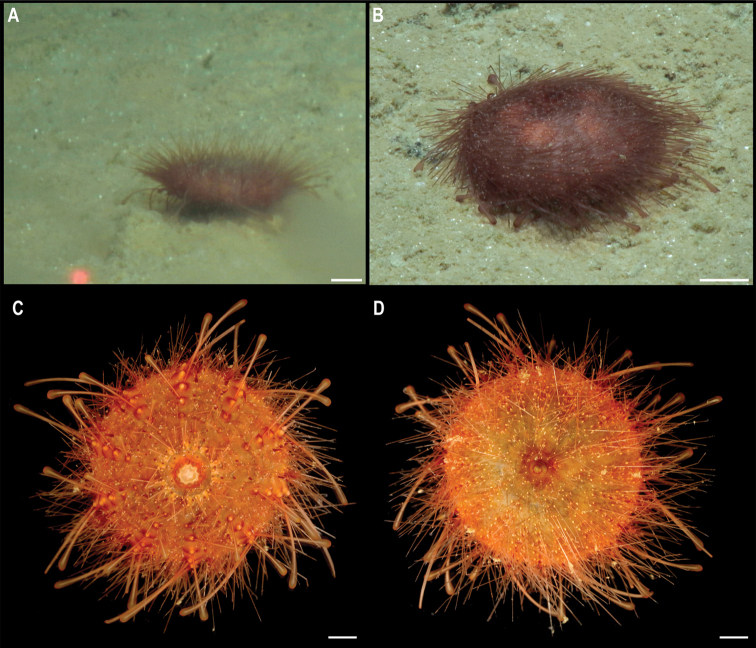
*Kamptosomaabyssale* Mironov, 1971. Specimen CCZ_082: **A, B** in situ images **C** oral view **D** aboral view of specimen before preservation. Scale bars: 1 cm (**A, B**); 5 mm (**C, D**). Image attribution: Durden and Smith (**A, B**); Wiklund, Durden, Drennan, and McQuaid (**C, D**).

Single specimen (d = 3.4 cm, H = 1.6 cm). In situ, the body is reddish brown, rounded and flattened (Fig. 33A, B). Spines are the same reddish brown colour of the body; oral primary spines are encased by a fleshy, clear sac, swollen and brighter at the tip. The test and covering skin are very thin and gonads are visible through; primary spines are projected upwards and tube feet extending downwards from the lower half of the body. Whole abactinal surface (ambulacral and interambulacral) covered by primaries arranged in irregular lines along the median lines of the plates, with few secondaries or militaries near ambitus (Fig. 33C). Claviform (globiferous) pedicellariae carries two saccules and two valves. Before preservation, colouration was bright orange.

###### Remarks.

Only two species of *Kamptosoma* have been described to date. *Kamptosomaasterias* (Agassiz, 1881) was first described from off the coast of Chile at 3950 m depth (type locality: H.M.S *Challenger* St. 299), and from the east of Malden Island, Central Pacific, at 4750 m depth (type locality for *K.indistinctum* synonymous with *K.asterias*: H.M.S. Challenger St. 272) (Agassiz 1881; 1904). It has also been reported for the central Pacific Ocean, the Tasman Sea, Chile, Antarctica, and the southern Indian Ocean from 3890–4950 m depth (Anderson 2016). *Kamptosomaabyssale* type locality is the Kuril-Kamchatka Trench, from 6090–6235 m (Mironov 1971), and occurs in the Northwest Pacific, from Aleutian Islands to Kermadec Trench, and from Madagascar to east of Hawaii between 4374–6235 m depth (Mooi et al. 2004). These two species are only differentiated by the shape of the claviform pedicellaria (previously referred to as globiferous pedicellaria, but Mironov et al. (2015) suggested these to belong to the claviform group as their rudimentary valves are not functional), with two valves in *K.abyssale*, and three valves in *K.asterias*. The specimen from the CCZ has pedicellariae with two saccules and two valves, as described for *K.abyssale*. However, the COI sequence is very similar (0.47–1.4% K2P distance) to sequences of *K.asterias* collected in the Tasman Sea from 4570–4744 m depth, and are recovered in a well-supported clade (Fig. 31). These values of genetic divergence are within the intraspecific divergence that has been reported for echinoids (Chow et al. 2016), and therefore might belong to the same species. Nonetheless these specimens identified as *K.asterias* were reported to have claviform pedicellariae with two valves as described for *K.abyssale* (Anderson 2016). It has been suggested that *K.abyssale* is a synonym of *K.asterias*, and the former species will only be validated once material from both type localities is examined in detail (Anderson 2016; Mironov et al. 2015; Mooi et al. 2004). *Kamptosomaasterias* from the Central Pacific (St. 272) has been re-examined and was reported to have pedicellariae with three valves (Mironov et al. 2015). The holotype from St. 299 was examined in this study. Unfortunately, most pedicellariae have been lost and only a single claviform pedicellariae was found, this with two valves.

###### Ecology.

The specimen was found crawling rapidly across abyssal sediment in APEI 4, at 5040 m depth. This morphotype has an unusually high crawling speed.

###### Comparison with image-based catalogue.

A very similar *Kamptosoma* sp. morphotype (i.e., *Kamptosomaabyssale* sp. inc., URC_010) has been encountered in seabed image surveys conducted in abyssal areas of Kiribati’s EEZ, but not in the eastern CCZ. URC_010 was the most abundant echinoid morphotype encountered in the abyssal areas explored within Kiribati’s EEZ (Simon-Lledó et al. 2019d).

**Class Holothuroidea**

**Figure 34.**
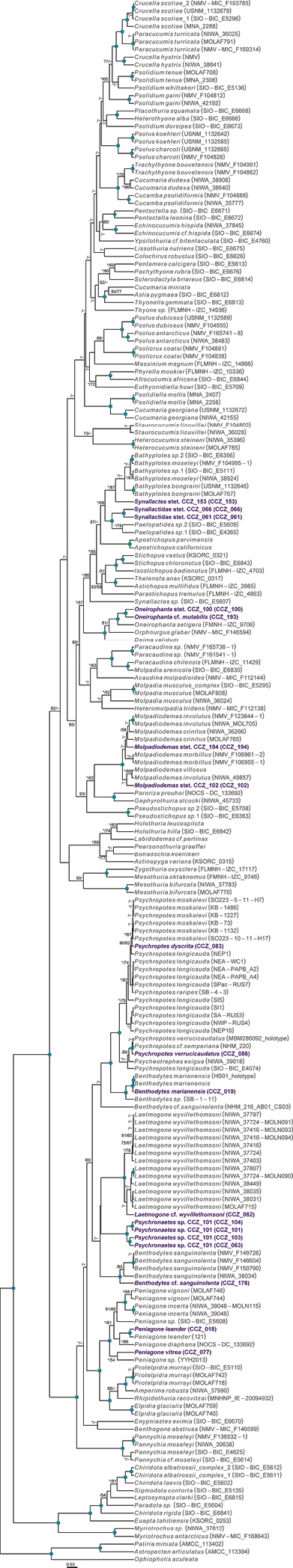
Phylogenetic tree of the class Holothuroidea. Concatenated (12S, 16S, 18S, 28S, COI, and H3) median consensus BEAST tree with posterior probability (PP) and bootstrap (BS) values indicated. Only values of PP > 0.70 and BS > 50 are shown, with values of PP > 0.95 and BS > 90 indicated with a circle. Nodes not recovered on the RAxML tree are indicated with a hyphen. Sequences generated in this study are highlighted in violet.

Holothurians are important components of the benthic deep-sea megafauna, and currently there are 367 records at a minimum depth of 3000 m in the CCZ, with 141 representing preserved specimens (OBIS 2022). Holothurians are amongst the most diverse invertebrate megafaunal taxa in the CCZ seafloor; a total of 106 different holothurian morphotypes has been so far catalogued in the image-based assessments consulted for this study, across the CCZ and nearby locations. We collected 18 specimens belonging to 15 different species, for which the COI gene was successfully amplified for all but one specimen. The gene 18S was successfully amplified for that specimen, as well as for other three. These were included in a concatenated alignment (12S, 16S, 18S, 28S, COI, and H3) used to estimate a phylogenetic tree (Fig. 34).

**Subclass Actinopoda Ludwig, 1891**

**Order Persiculida Miller, Kerr, Paulay, Reich, Wilson, Carvajal & Rouse, 2017**

**Family Molpadiodemidae Miller, Kerr, Paulay, Reich, Wilson, Carvajal & Rouse, 2017**

#### Genus *Molpadiodemas* Heding, 1935

##### ﻿ Molpadiodemas

Taxon classificationAnimaliaPersiculidaMolpadiodemidae

stet. CCZ_102

860E9C6F-966E-57FB-9B56-7356DACBB6E9

Fig. 35

###### Material.

Clarion-Clipperton Zone • 1 specimen; APEI 4; 7.2701°N, 149.7827°W; 3552 m deep; 03 Jun. 2018; Smith & Durden leg.; GenBank: ON400708 (COI); NHMUK 2022.66; Voucher code: CCZ_102.

###### Description.

**Figure 35.**
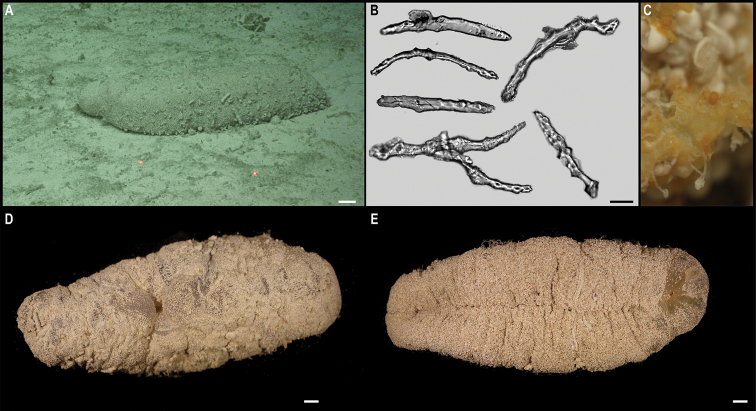
*Molpadiodemas* stet. CCZ_102 **A** in situ image **B** tentacle ossicles **C** tube feet **D** dorsal surface **E** ventral surface of specimen before preservation. Scale bars: 2 cm (**A**); 20 μm (**B**); 1 cm (**D, E**). Image attribution: Durden and Smith (**A**); Bribiesca-Contreras (**B, C**); Wiklund, Durden, Drennan, and McQuaid (**D, E**).

Single specimen, ~ 32 cm long (Fig. 35A). Body subcylindrical when alive, dorso-ventrally flattened in preserved specimen (L = 22 cm, W = 9 cm; Fig. 35E, F), tapering distally; body wall is completely covered in sediment and globigerinas, firm, wrinkly, with transverse folds and ridges giving a partly serrated appearance to the margin; brim present; anus and mouth ventral (Fig. 35D, E). Tube feet only visible on the ventral surface, cylindrical, and orange (Fig. 35C). Dorsal surface is whitish and ventral is yellowish in preserved specimen, but heavily covered by sediment. Ossicles in tentacles; unbranched rods and branched rods, with branches intertwining at the ends creating irregular perforated mesh (Fig. 35B).

###### Remarks.

COI sequence forms a clade with other species of *Molpadiodemas* including *M.villosus* (Théel, 1886), *M.morbillus* O’Loughlin & Ahearn, 2005, *M.crinitus* O’Loughlin & Ahearn, 2005 and *M.involutus* (Sluiter, 1901). Closest is to *M.morbilus* (K2P 3.7–3.9%), and in the phylogenetic tree it is recovered in a well-supported clade with other species within the genus (Fig. 34), but species were not separated within the genus. Three species within the genus *Molpadiodemas* have been previously reported in the CCZ: *M.altanticus* (R. Perrier, 1898), *M.villosus* and *M.helios* O’Loughlin & Ahearn, 2005, with the latter species being recently described from the CCZ and so far not reported elsewhere (O’Loughlin and Ahearn 2005). However, morphological characters of *Molpadiodemas* stet. CCZ_102 are not in accordance with the description of any of those three described species.

###### Ecology.

This specimen was collected on the sediment seafloor of a seamount in APEI 4 at 3552 m depth.

###### Comparison with image-based catalogue.

A very similar Molpadiodemidae morphotype (i.e., *Molpadiodemas* sp. indet., HOL_103) has been commonly encountered in seabed image surveys conducted across the eastern CCZ (e.g., Amon et al. 2017b) and in abyssal areas of the Kiribati EEZ, mostly in nodule field areas.

##### ﻿ Molpadiodemas

Taxon classificationAnimaliaPersiculidaMolpadiodemidae

stet. CCZ_194

E00FBA21-0CEF-58FE-AF03-9DC6E4BA83C9

Fig. 36

###### Material.

Clarion-Clipperton Zone • 1 specimen; APEI 1; 11.2517°N, 153.6055°W; 5205 m deep; 10 Jun. 2018; Smith & Durden leg.; GenBank: ON400725 (COI); NHMUK 2022.71; Voucher code: CCZ_194.

###### Description.

**Figure 36.**
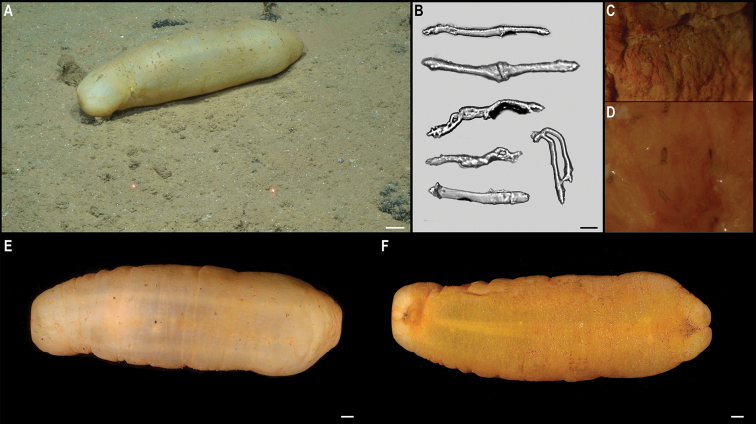
*Molpadiodemas* stet. CCZ_194 **A** in situ image **B** tentacle ossicles **C** epibionts on ventral surface **D** detail of epibionts **E** dorsal surface **F** ventral view of specimen before preservation. Scale bars: 2 cm (**A**); 25 μm (**B**); 1 cm (**E, F**). Image attribution: Durden and Smith (**A**); Bribiesca-Contreras (**B–D**); Wiklund, Durden, Drennan, and McQuaid (**E, F**).

Single specimen (Fig. 36A). Colouration of live specimen is whitish yellow, with skin somewhat translucent (Fig. 36A, E). Body subcylindrical in live specimen, but dorso-ventrally flattened when preserved, tapering anteriorly, ~ 4× as long as wide (L = 25 cm, W = 8.2 cm); semi-translucent body wall, longitudinal muscles visible through it; colouration of preserved specimen is yellowish (Fig. 36E), darker on the ventral side (Fig. 36F). Ventral surface with small, black, unidentified epibionts embedded in the skin (Fig. 36C, D). Specimen barely covered by sediment. Ossicles in tentacles; unbranched rods with thick central swelling; and branched rods, often with branches intertwining at the ends creating an irregular perforated mesh (Fig. 36B).

###### Remarks.

The COI sequence of *Molpadiodemas* stet. CCZ_194 is similar to sequences of other species of *Molpadiodemas*, including *M.villosus*, *M.morbillus*, *M.crinitus*, *M.involutus*, and *Molpadiodemas* stet. CCZ_102. COI genetic divergence between both specimens collected in the CCZ is 6%, in accordance with values of genetic interspecific divergence for the genus. The specimen is recovered in a well-supported clade along with other members of the genus (Fig. 34), but species are not well delimited. As mentioned above, three species of *Molpadiodemas* have been previously reported in the CCZ (O’Loughlin and Ahearn 2005). The tentacle ossicles from specimen CCZ_194 are very similar to those of *M.helios*, but this latter species is distinguished by the prominent tube feet that are barely visible in our specimen.

###### Ecology.

This specimen was found on the sedimented seafloor of an abyssal plain on APEI 1 at 5205 m depth.

###### Comparison with image-based catalogue.

A very similar *Molpadiodemas* sp. morphotype (i.e., *Molpadiodemas* sp. indet., HOL_004) has been commonly encountered in seabed image surveys conducted across nodule fields areas of the eastern CCZ (e.g., Amon et al. 2017b), but not in abyssal areas surveyed within the Kiribati EEZ.

**Order Synallactida Miller, Kerr, Paulay, Reich, Wilson, Carvajal & Rouse, 2017**

**Family Synallactidae Ludwig, 1894**

**Synallactidae stet. CCZ_061**

Fig. 37

**Material.** Clarion-Clipperton Zone • 1 specimen; APEI 7; 4.8877°N, 141.7569°W; 3132 m deep; 27 May. 2018; Smith & Durden leg.; GenBank: ON400688 (COI), ON406640 (18S); NHMUK 2022.75; Voucher code: CCZ_061.

**Figure 37.**
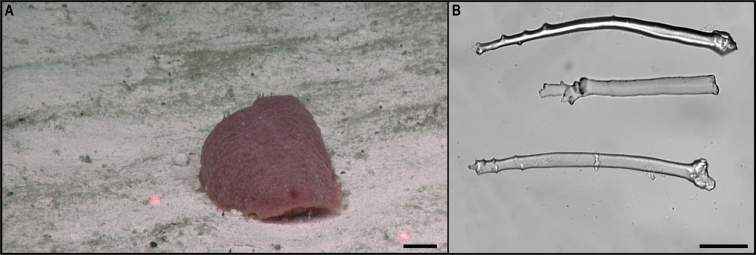
Synallactidae stet. CCZ_061 **A** in situ image **B** tentacle ossicles. Scale bars: 2 cm (**A**); 50 μm (**B**). Image attribution: Durden and Smith (**A**); Bribiesca-Contreras (**B**).

**Description.** Single specimen; description of external morphological features only from in situ image as the specimen was damaged during collection (Fig. 37A). Body semi-circular, with ventral surface flattened, tapering distally; very wide, widest part of body ~ 7 cm; anus posterodorsal; mouth anteroventral. Tegument seems thick. Ossicles present in tentacles, slightly curved rods, < 250 μm (Fig. 37B).

**Remarks.** There are no close matches to the COI sequence of Synallactidae stet. CCZ_061 in public databases. The closest match is to Synallactidae stet. CCZ_066 (14% K2P). Both specimens are recovered in a well-supported clade representing the family Synallactidae (Fig. 34). They are recovered very close to *Paelopatides* Théel, 1886, but COI divergence suggest they belong to different genera.

**Comparison with image-based catalogue.** No similar Synallactidae morphotypes have been so far catalogued from seabed imagery collected in the eastern CCZ or in abyssal areas of the Kiribati EEZ. Consequently, the in situ image of Synallactidae stet. CCZ_061 was catalogued as a new morphotype (i.e., Synallactidae gen. indet., HOL_120).

**Ecology.** This specimen was collected on the sedimented seafloor of a seamount in APEI 7 at 3132 m depth.

**Synallactidae stet. CCZ_066**

Fig. 38

**Material.** Clarion-Clipperton Zone • 1 specimen; APEI 7; 4.8896°N, 141.75°W; 3095 m deep; 27 May. 2018; Smith & Durden leg.; GenBank: ON400692 (COI), ON406642 (18S); NHMUK 2022.63; Voucher code: CCZ_066.

**Figure 38.**
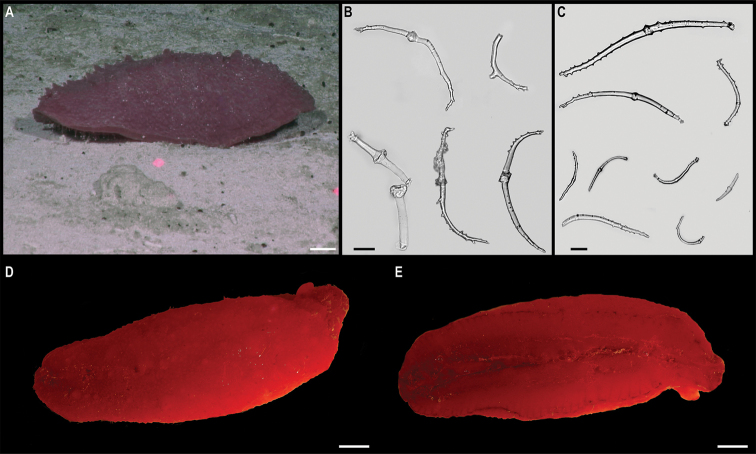
Synallactidae stet. CCZ_066 **A** in situ image **B** ossicles on dorsal skin **C** tentacle ossicles **D** dorsal surface **E** ventral surface of specimen before preservation. Scale bars: 2 cm (**A, D, E**); 50 μm (**B, C**). Image attribution: Durden and Smith (**A**); Bribiesca-Contreras (**B, C**); Wiklund, Durden, Drennan, and McQuaid (**D, E**).

**Description.** Single specimen, body semi-circular with ventral surface flattened; ~ 3× longer than wide (L = 21 cm, W = 6 cm; Fig. 38A). Mouth anteroventral, anus posterodorsal. Colouration in live specimen is bright red (Fig. 38D, E). Specimen severely damaged during collection, guts separated from skin. Tegument is thick and leathery, with wart-like protrusions on the dorsal surface, more evident on live specimen (Fig. 38A), and with a small, very short, triangular dorsal appendage. Brim evident on ventral surface. Small tube feet arranged in two irregular rows, one on each side of ventrum, running longitudinally. Tentacles 18. Ossicles present in dorsal skin (Fig. 38B) and tentacles (Fig. 38C).

**Remarks.** There are no close matches to the COI sequence of Synallactidae stet. CCZ_066 in public databases. The closest match is to specimen Synallactidae stet. CCZ_061 (14% K2P). Both specimens are recovered in a well-supported clade representing the family Synallactidae (Fig. 34). They are recovered very close to *Paelopatides* Théel, 1886, but COI divergence suggest they belong to different genera.

**Ecology.** This specimen was collected on the sedimented seafloor of a seamount on APEI 7 at 3095 m depth.

**Comparison with image-based catalogue.** No similar Synallactidae morphotypes have been so far catalogued from seabed imagery collected in the eastern CCZ nor in abyssal areas of the Kiribati EEZ. Consequently, the in situ image of CCZ_066 was catalogued as a new morphotype (i.e., Synallactidae gen. indet., HOL_121). The dorsal protrusions that differentiate HOL_121 from HOL_120 may not be clearly visible in vertically-facing seabed imagery, and hence these two taxa might only be classifiable into a single, generic morphotype (i.e., HOL_120) in quantitative analyses.

#### Genus *Synallactes* Ludwig, 1894

##### ﻿ Synallactes

Taxon classificationAnimaliaSynallactidaSynallactidae 

stet. CCZ_153

8DE6E9D2-1DE4-525E-8571-2A9207A4C66C

Fig. 39

###### Material.

Clarion-Clipperton Zone • 1 specimen; APEI 4; 6.9704°N, 149.9426°W; 5009 m deep; 06 Jun. 2018; Smith & Durden leg.; GenBank: ON400714 (COI); NHMUK 2022.69; Voucher code: CCZ_153.

###### Description.

**Figure 39.**
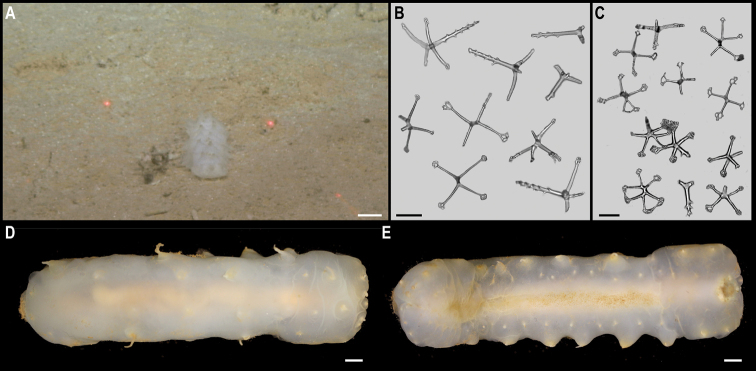
*Synallactes* stet. CCZ_153 **A** in situ image **B** ossicles from dorsal skin **C** ossicles from ventral skin **D** dorsal view of specimen before preservation, **E** ventral view. Scale bars: 2 cm (**A**); 50 μm (**B, C**); 5 mm (**D, E**). Image attribution: Durden and Smith (**A**); Bribiesca-Contreras (**B, C**); Wiklund, Durden, Drennan, and McQuaid (**D, E**).

Single specimen (Fig. 39A). Body cylindrical, white, ~ 4× as long as wide (L = 10 cm, W = 2.7 cm), flattened proximally and rounded distally; flattened ventral surface. Two rows, upper and lower, of lateral, small, conical, thin processes, similar to those around the proximal edge (Fig. 39D). There is a row of yellowish, very small, tube feet in the mid-ventral surface, along the odd ambulacrum (Fig. 39E). Skin firm but translucent. Colour on live and preserved specimen is white. Ossicles abundant on dorsal body wall, spatulated crosses only with a long spinous apophysis, end of arms spatulated with holes (Fig. 39B). Ventral ossicles also spatulated crosses with a long spinous apophysis, smaller, sometimes with more than four arms, also spatulated ends of arms with holes (Fig. 39C).

###### Remarks.

The closest matches for the barcoding gene COI sequence are published sequences from the genus *Bathyplotes* (89.9% similarity), also within the family Synallactidae. The sequence is distinct from the only sequence of *Synallactes* sp. (GenBank accession number: KX874365.1) included in the phylogeny (Fig. 34), and they were not recovered as a monophyletic group. The DeepCCZ specimen was recovered sister to species of *Bathyplotes*, with *Synallactes* sp. recovered separately from the other genera in the family Synallactidae, concordant with previous results (Miller et al. 2017). Despite this, the specimen was assigned to the genus *Synallactes* based on external morphological characters that are concordant with those described from the genus. Species of *Synallactes* have previously been reported in the CCZ: *Synallactesprofundus* (Koehler & Vaney, 1905) and *Synallactesaenigma* Ludwig, 1894; the latter being associated with manganese substrates. External morphology does not resemble to *S.profundus*.

###### Ecology.

This specimen was found on the sedimented seafloor of an abyssal plain on APEI 4 at 5008 m depth.

###### Comparison with image-based catalogue.

A very similar Synallactidae morphotype (i.e., *Synallactes* sp. indet., HOL_007) has been commonly encountered in seabed image surveys conducted across nodule field areas of the eastern CCZ (e.g., Amon et al. 2017b), but not in abyssal areas of the Kiribati EEZ, where synallactid specimens were very rarely encountered.

**Family Deimatidae Théel, 1882**

#### Genus *Oneirophanta* Théel, 1879

##### ﻿ Oneirophanta

Taxon classificationAnimaliaSynallactidaDeimatidae

stet. CCZ_100

9A805BDC-1CEE-58CC-806A-67B4AA80B026

Fig. 40

###### Material.

Clarion-Clipperton Zone • 1 specimen; APEI 4; 7.2647°N, 149.774°W; 3550 m deep; 03 Jun. 2018; Smith & Durden leg.; GenBank: ON400706 (COI), ON406643 (18S), ON406620 (16S); NHMUK 2022.84; Voucher code: CCZ_100.

###### Description.

**Figure 40.**
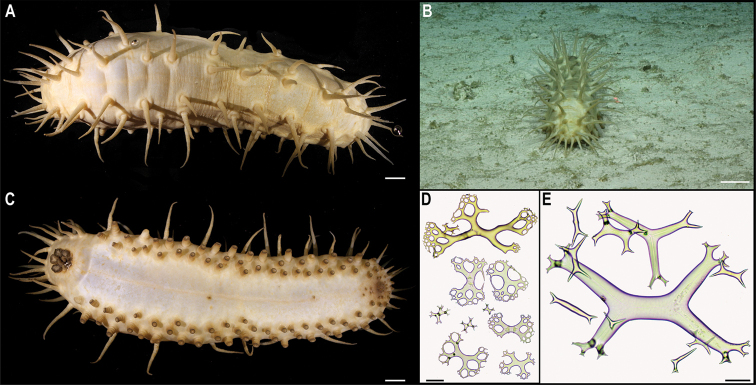
*Oneirophanta* stet. CCZ_100 **A** dorsal view of specimen before preservation **B** in situ image **C** ventral view **D** dorsal ossicles **E** ventral ossicles. Scale bars: 2 cm (**A, C**); 5 cm (**B**); 200 μm (**D**); 100 μm (**E**). Image attribution: Wiklund, Durden, Drennan, and McQuaid (**A, C**); Durden and Smith (**B**); Kremenetskaia (**D, E**).

Single specimen; colouration of live specimen is beige, spotted with light brown and yellow on dorsal surface (Fig. 40A, C, D), and lighter on ventral surface, with suckers on tube feet and tentacles being dark brown (Fig. 40D). Body cylindrical, ~ 33 cm long and 8.8 cm wide; mouth anteroventral, anus posteroventral. Tentacles partly retracted. Papillae arranged in one or two rows along the dorsal radii, and in a single row along the ventrolateral radii above the tube feet. Tube feet ~ 50 pairs, arranged in two or three rows on each ventrolateral ambulacrum; few tube feet located along mid-ventral ambulacrum, among them two tube feet, one placed approx. half the body length and the other approx. three quarter of the body length; and few smaller feet close to anus. Dorsal ossicles spatulated crosses, crosses with open ramifications, and small irregular perforated plates; ventral ossicles crosses with open ramifications of different stage of development.

###### Remarks.

Closest match for COI and 16S sequences is to *Oneirophantasetigera* (Ludwig, 1893) (86.7% and 96.3%, respectively). In the phylogenetic tree, it is recovered in a well-supported clade representing the family Deimatidae, including *Oneirophanta* (Fig. 34). According to the external morphology *Oneirophanta* sp. CCZ_100 differs from *Oneirophantamutabilismutabilis* Théel, 1879, *O.mutabilisaffinis* Ludwig, 1893 and *O.conservata* Koehler & Vaney, 1905 in high number of tube feet arranged in two or three rows and by absence of large, perforated plates on dorsum. It differs from *O.setigera* in high number of tube feet arranged in two or three rows and by presence of small, perforated plates and bigger perforations on spatulated crosses.

###### Ecology.

The specimen was found on the sediment seafloor of a seamount on APEI 4 at 3550 m depth.

###### Comparison with image-based catalogue.

No exactly similar Deimatidae morphotypes have been so far catalogued from seabed imagery collected in the eastern CCZ nor in abyssal areas of the Kiribati EEZ. Consequently, the in situ image of CCZ_100 was catalogued as a new morphotype (i.e., *Oneirophanta* sp. indet., HOL_063). However, HOL_063 could be potentially confused with a similar shaped Deimatidae morphotype (e.g., Deimatidae gen. indet., HOL_062; also beige, cylindrical, with conspicuous projections on the dorsal surface arranged in four rows) found in the eastern CCZ (e.g., Amon et al. 2017b), with more abundant -though slightly thinner- projections, that may be difficult to distinguish in vertically facing images.

##### ﻿ Oneirophanta cf. mutabilis

Taxon classificationAnimaliaSynallactidaDeimatidae

Théel, 1879

DA9FD0E4-A8B0-53EE-922C-254918C4D596

Fig. 41

###### Material.

Clarion-Clipperton Zone • 1 specimen; APEI 1; 11.252°N, 153.5847°W; 5203 m deep; 10 Jun. 2018; Smith & Durden leg.; GenBank: ON400724 (COI), ON406629 (18S), ON406619 (16S); NHMUK 2021.20; Voucher code: CCZ_193.

###### Other material.

Indian Ocean • 3 specimens, syntypes of *Oneirophantamutabilis* Théel, 1879; Eastern Indian, Antarctic Basin; 53.9167° S, 108.5833° E; 3566 m deep; Challenger Expedition, Stn. 157; NHMUK 1883.6.18.33.

###### Description.

**Figure 41.**
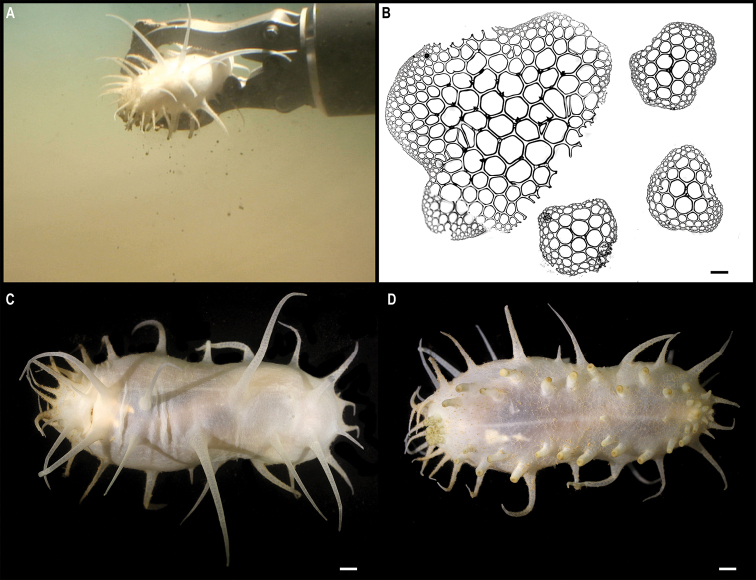
Oneirophantacf.mutabilis Théel, 1879. Specimen CCZ_193 **A** in situ image **B** dorsal ossicles **C** dorsal view before preservation **D** ventral view. Scale bars: 200 μm (**B**); 1 cm (**C, D**). Image attribution: Durden and Smith (**A**); Bribiesca-Contreras (**B**); Wiklund, Durden, Drennan, and McQuaid (**C, D**).

Single specimen, body uniformly white (Fig. 41A). Body almost cylindrical, > 2× as long as wide (L = 16 cm; W = 6.9 cm), being of almost equal breadth throughout the whole length and tapering posteriorly; mouth anteroventral and anus posteroventral. Tentacles 20, small, with a lightly brown tip, each with a terminal part with 6–8 unbranched processes. Long, pointed processes, or different lengths, arranged in four distinct rows, two rows running along the dorsal ambulacra with eight processes on each row, and the longest being approx. half of the body length (Fig. 41C); the other two rows placed on the sides of the body, slightly above the ventral lateral ambulacra. Ventral ambulacra with 11 and 13 tube feet, arranged in two irregular rows; odd ambulacrum naked, except for two tube feet arranged in the posterior half of the body, and ten surrounding the anus; processes crowded anteriorly (Fig. 41D). Thin skin, translucent, but hard and brittle, with numerous small and large perforated plates, with the small ones bearing two or three spines near the centre, and the large ones ~ 30 spines; ossicles imbricated, almost forming a skeleton (Fig. 41B).

###### Remarks.

Sequences for the 18S, 16S, and COI genes were most similar to sequences from *Oneirophantasetigera* (99.07%, 95.6%, 88.51% similarity, respectively), followed by other species within the family Deimatidae (i.e., *Orphnurgusglaber* Walsh, 1891 and *Deimavalidum* Théel, 1879). The specimen was recovered in a well-supported clade including all members of Deimatidae (Fig. 34), closest to *Oneirophanta* sp. CCZ_100 (K2P distance: 11%). Calcareous ossicles are concordant with those in *Oneirophantamutabilis*. This species was originally described west of the Crozet Islands (H.M.S. Challenger station 146: 46.7667°S, 45.5167°E) at 2514 m depth (Théel 1879) but has been further divided in two sub-species, *O.mutabilismutabilis* and *O.mutabilisaffinis*. The former has been reported to be cosmopolitan, while the later has been recorded from the tropical eastern Pacific. Further analyses will be required to determine the validity of the subspecies and if the CCZ specimen belongs to any of those. It differs from the original description of *O.mutabilis* (Théel, 1879) in having an irregular number of pedicels around the ventral surface, the pedicels around the anus arranged triangularly instead of a transversal row, as well as the arrangement of the processes on bivium and trivium. However, Théel (1879) mentioned that several of the specimens examined differed from the specimen described in the number of pedicels and the arrangement of processes, and further studies should clarify if these are indeed subspecies.

###### Ecology.

The specimen was found on the sediment surface of an abyssal plain on APEI 1 at 5203 m depth.

###### Comparison with image-based catalogue.

A very similar *Oneirophanta* sp. morphotype (i.e., *Oneirophanta* sp. indet., HOL_058) has been encountered in seabed image surveys conducted across nodule fields areas of the eastern CCZ (e.g., Amon et al. 2017b), but not in the abyssal areas surveyed at the Kiribati EEZ.

**Order Elasipodida Théel, 1882**

**Family Psychropotidae Théel, 1882**

#### Genus *Psychropotes* Théel, 1882

##### ﻿ Psychropotes verrucicaudatus

Taxon classificationAnimaliaElasipodidaPsychropotidae

Xiao, Gong, Kou, Li, 2019

DE7D0BEC-D4C3-5592-965D-E11F0D130D35

Fig. 42

###### Material.

Clarion-Clipperton Zone • 1 specimen; APEI 4; 6.9878°N, 149.9119°W; 4999 m deep; 02 Jun. 2018; Smith & Durden leg.; GenBank: ON400703 (COI); NHMUK 2021.19; Voucher code: CCZ_086.

###### Description.

**Figure 42.**
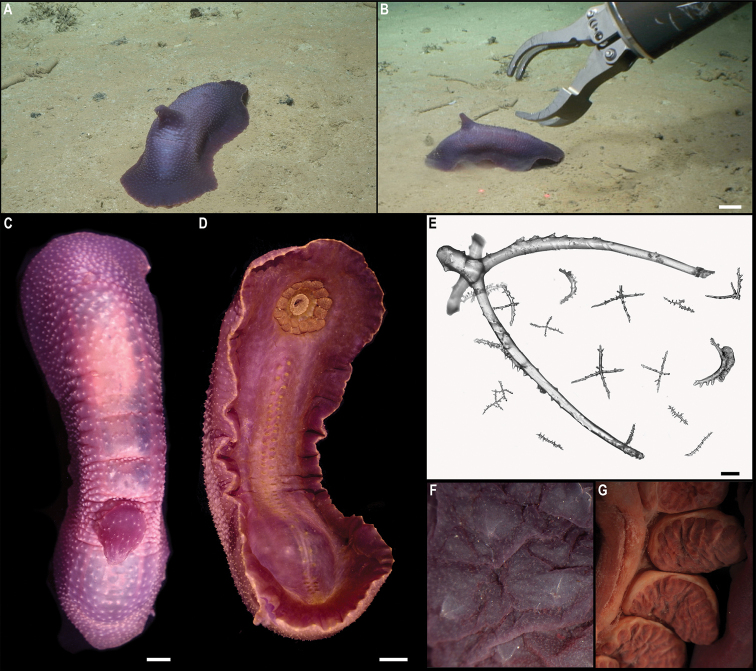
*Psychropotesverrucicaudatus* Xiao, Gong, Kou, Li, 2019. Specimen CCZ_086: **A, B** in situ images **C** dorsal view of specimen before preservation **D** ventral view **E** dorsal ossicles **F** detail of warts and ossicles on dorsal body wall **G** mouth tentacles. Scale bars: 5 cm (**B**); 2 cm (**C, D**); 100 μm (**E**). Image attribution: Durden and Smith (**A, B**); Wiklund, Durden, Drennan, and McQuaid (**C, D**); Bribiesca-Contreras (**E–G**).

Single specimen, colouration in situ is violet (Fig. 42A, B). Body elongated and anteriorly depressed (L = 34.7 cm, W = 10.2 mm); with a broad brim. Short (approx. one twelfth of body length), conical, single-pointed, dorsal unpaired appendage, placed 2/5 of the body length from the posterior end (Fig. 42A–C). Dorsal skin, including the dorsal appendage, covered in warts (Fig. 42C, F). Each wart has an ossicle in the centre, a giant cross with a central apophysis and strongly curved arms, all visible through the skin (Fig. 42E, F). Dorsal skin also contains smaller crosses with spiny arms (Fig. 42E, F). Approximately 30 pairs of mid-ventral tube feet arranged in two rows along the mid-ventral ambulacrum, arranged very close together on the anterior two thirds of the body, and scattered after posteriorly, with the last pairs being very close together again. Colouration of preserved specimen is also purple, with slightly lighter ventrum.

###### Remarks.

COI sequence is very similar (K2P distance = 0.77%) to the holotype of *P.verrucicaudatus*, and they were recovered together in the phylogenetic tree (Fig. 34). This species was described from the Jiaolong seamount, in the South China Sea, western Pacific Ocean at 3615 m deep (Xiao et al. 2019). External morphological characters are in accordance with the original description.

###### Ecology.

The specimen was found on the sedimented abyssal plain in APEI 4 at 4999 m depth.

###### Comparison with image-based catalogue.

A very similar *Psychropotes* sp. morphotype (i.e., *Psychropotesverrucicaudatus* sp. inc., HOL_045) has been commonly encountered in seabed image surveys conducted across nodule fields areas of the eastern CCZ (e.g., Amon et al. 2017b), but not in the abyssal areas surveyed within the Kiribati EEZ.

##### ﻿ Psychropotes dyscrita

Taxon classificationAnimaliaElasipodidaPsychropotidae 

(Clark, 1920)

2C1F5292-4D55-5700-8AC8-D59FB002D0D0

Fig. 43

###### Material.

Clarion-Clipperton Zone • 1 specimen; APEI 4; 7.0212°N, 149.9355°W; 5040 m deep; 02 Jun. 2018; Smith & Durden leg.; GenBank: ON400702 (COI); NHMUK 2022.83; Voucher code: CCZ_083.

###### Description.

**Figure 43.**
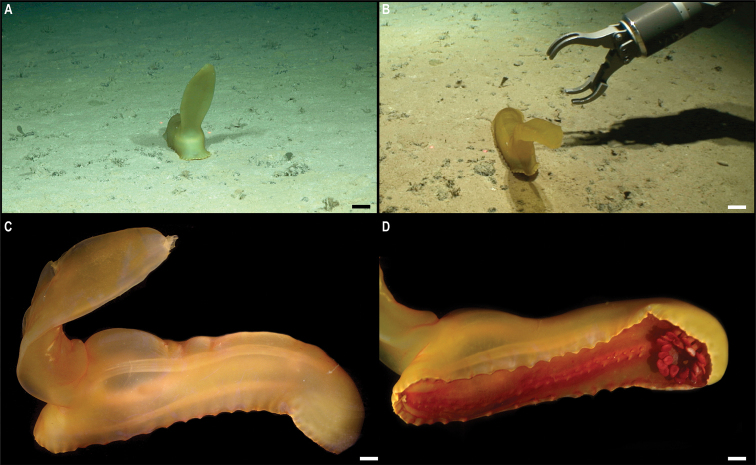
*Psychropotesdyscrita* (Clark, 1920). Specimen CCZ_083: **A, B** in situ images **C** lateral view **D** ventral view. Scale bars: 5 cm (**A, B**); 2 cm (**C, D**). Image attribution: Durden and Smith (**A, B**); Wiklund, Durden, Drennan, and McQuaid (**C, D**).

Single specimen, ~ 30 cm long (Fig. 43A). Colouration of live specimen is yellow (Fig. 43A, B), with reddish-light purple on ventral surface (Fig. 43C). Tentacles 18, also reddish-light purple. Long, dorsal appendage with round end, slightly longer than the total body length, and developed very close to the posterior end of the body.

###### Remarks.

Gebruk et al. (2020) morphologically examined the specimen collected during the DeepCCZ and re-established the species *Psychropotesdyscrita* based on this specimen. The holotype was collected in Peru, at 5206 m depth, and the species is known from the Central Pacific Ocean at depths of 5040–5206 m (Gebruk et al. 2020). *Psychropotesdyscrita* and *P.moskalevi* Gebruk & Kremenetskaia, 2020 are the two only known yellow species for this genus and were recovered as sister species (Fig. 34). The COI sequence for the DeepCCZ specimen is 1.1 ± 0.4% divergent (K2P distance) from specimens of *P.moskalevi*. Although this value seems low, the COI gene seems to be more conserved in the genus *Psychropotes* (1.1–13.4%, mean = 6.5%), with < 2% interspecific divergence between some species pairs (*P.dyscrita*-*P.moskalevi*, *P.moskalevi*-*P.raripes* Ludwig, 1893).

###### Ecology.

The specimen was found on the sediment seafloor of an abyssal plain in APEI 4 at 5040 m depth.

###### Comparison with image-based catalogue.

A very similar *Psychropotes* sp. morphotype (i.e., *Psychropotes* sp. indet., HOL_047) has been encountered in seabed image surveys conducted across nodule fields areas of the eastern CCZ (e.g., Tilot 2006), and in the Kiribati EEZ, where this taxon was the most abundant holothurian encountered (Simon-Lledó et al. 2019d). In pioneer seabed image surveys conducted at the CCZ, prior to the re-establishment of the species (Gebruk et al. 2020), this morphotype was typically classified as *P.longicauda*.Based on seabed imagery (e.g., without analysis of ossicles), it is not possible to determine whether HOL_047 specimens are *P.dyscrita* or *P.moskalevi*.

#### Genus *Benthodytes* Théel, 1882

##### ﻿ Benthodytes cf. sanguinolenta

Taxon classificationAnimaliaElasipodidaPsychropotidae

Théel, 1882

7EEBDE34-5394-57F9-94F5-82773FCBAC7A

Fig. 44

###### Material.

Clarion-Clipperton Zone • 1 specimen; APEI 1; 11.2953°N, 153.742°W; 5245 m deep; 09 Jun. 2018; Smith & Durden leg.; GenBank: ON400720 (COI); NHMUK 2022.70; Voucher code: CCZ_178.

###### Description.

**Figure 44.**
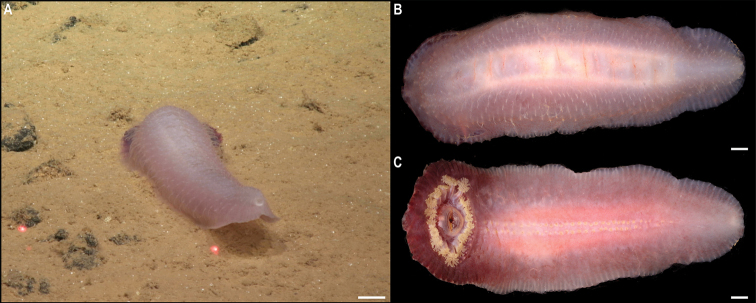
Benthodytescf.sanguinolenta Théel, 1882. Specimen CCZ_178: **A** in situ image **B** dorsal view of specimen before preservation **C** ventral view. Scale bars: 2 cm (**A**); 1 cm (**B, C**). Image attribution: Durden and Smith (**A**); Wiklund, Durden, Drennan, and McQuaid (**B, C**).

Single specimen (Fig. 44A). Colouration of live specimen is light pink dorsally (Fig. 44B), darker ventrally (Fig. 44C). Tentacles 18, yellow, digitiform. Numerous dorsal papillae scattered on dorsal. Brim wide. Tube feet in double rows along the mid-ventral ambulacrum, ~ 30 pairs, yellowish. Ossicles not found.

###### Remarks.

The closest match for the COI sequence is a sequence from *B.sanguinolenta* (GenBank: HM196505.1; 93.54% similarity) from the Ross Sea, Antarctica. A genetic study revealed two separate clades within *B.sanguinolenta* (O’Loughlin et al. 2011): (1) specimens from northwest Australia, and (2) Ross Sea. None of the samples included in O’Loughlin et al. (2011) are from the type locality (34.1167° S 73.9399°’W, off Chile, Pacific Ocean; 4000 m), but they identified at least two separate genetic species. The COI sequence of the specimen collected in the CCZ forms a third clade within the *B.sanguinolenta* species complex (Fig. 34). Genetic divergence (K2P distance) between the CCZ specimens and both NW Australia and Ross Sea clades is 10.1% and 7.3%, respectively, corresponding to values of intraspecific divergence in the group. In original description, Théel (1882) describes the body to be 6–7× longer than wide, whereas the preserved specimen collected in the CCZ is only ~ 3× longer than wide, but might be due to preservation as in in situ images it appears longer. However, the number of digitiform tentacles and appearance of small processes are concordant with the description of *B.sanguinolenta*. The sequence of Benthodytescf.sanguinolenta from Glover et al. (2016b) does not form a clade with the CCZ specimen, with COI genetic distance being large (K2P 23%).

###### Ecology.

The specimen was found on the sedimented seafloor of an abyssal plain in APEI 1 at 5249 m depth.

###### Comparison with image-based catalogue.

No exactly similar *Benthodytes* sp. morphotypes have been so far catalogued from seabed imagery collected in the eastern CCZ or in abyssal areas of the Kiribati EEZ. Consequently, the in situ image of specimen CCZ_178 was catalogued as a new morphotype (i.e., *Benthodytessanguinolenta* sp. inc., HOL_124).

##### ﻿ Benthodytes marianensis

Taxon classificationAnimaliaElasipodidaPsychropotidae

Li, Xiao, Zhang & Zhang, 2018

07DA9927-D709-58BC-9828-6BF452AB4064

Fig. 45

###### Material.

Clarion-Clipperton Zone • 1 specimen; APEI 7; 5.1043°N, 141.8865°W; 4861 m deep; 25 May. 2018; Smith & Durden leg.; GenBank: ON400682 (COI); NHMUK 2022.82; Voucher code: CCZ_019.

###### Description.

**Figure 45.**
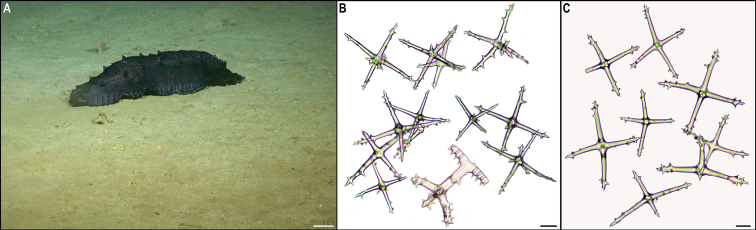
*Benthodytesmarianensis* Li, Xiao, Zhang & Zhang, 2018. CCZ_019: **A** in situ image **B** dorsal ossicles including peculiar cross-shaped ossicle **C** ventral ossicles. Scale bar: 5 cm (**A**); 100 µm (**B, C**). Image attribution: Durden and Smith (**A**), Kremenetskaia (**B, C**).

Single specimen (Fig. 45). Body is elongated, ~ 49.4 cm, dorso-ventrally flattened with flat ventral surface and inflated dorsal surface; anteriorly depressed and tapering posteriorly; colouration in live specimen is dark violet. Two irregular rows of large conical papillae running along the paired dorsal ambulacra.

###### Remarks.

The COI sequence is identical to the holotype of *B.marianensis* (K2P genetic distance = 0%) collected in the Mariana Trench at 5567 m depth (Li et al. 2018). These two sequences are also recovered together in the phylogenetic tree (Fig. 34). The species is only known from this location. Morphological characters are also concordant with the original description, including an uncommon, very peculiar, cross-shaped, dorsal ossicle (Fig. 45B).

###### Ecology.

The specimen was found on the sedimented seafloor of an abyssal plain in APEI 7 at 4860 m depth.

###### Comparison with image-based catalogue.

CCZ_019 resembles a *Benthodytes* sp. morphotype (i.e., *Benthodytes* sp. indet., HOL_111) encountered in seabed image surveys conducted across nodule fields areas of the eastern CCZ (Amon et al. 2017b) and the Kiribati EEZ. However, the vivid dark/violet colouration of HOL_011 (contrasting with background bright sediment) can constrain the visibility of papillae features in in situ photographed specimens, potentially making these hard to differentiate from other *Benthodythes* sp. morphotypes in vertically-facing seabed imagery.

**Family Elpidiidae Théel, 1882**

#### Genus *Peniagone* Théel, 1882

##### ﻿ Peniagone leander

Taxon classificationAnimaliaElasipodidaElpidiidae

Pawson & Foell, 1986

DFCD19FD-1EBA-5FD9-8FAF-BD1DE8BC0981

Fig. 46

###### Material.

Clarion-Clipperton Zone • 1 specimen; APEI 7; 5.1042°N, 141.8861°W; 4860 m deep; 25 May. 2018; Smith & Durden leg.; GenBank: ON400681 (COI), ON406621 (16S); NHMUK 2022.61; Voucher code: CCZ_018.

###### Description.

**Figure 46.**
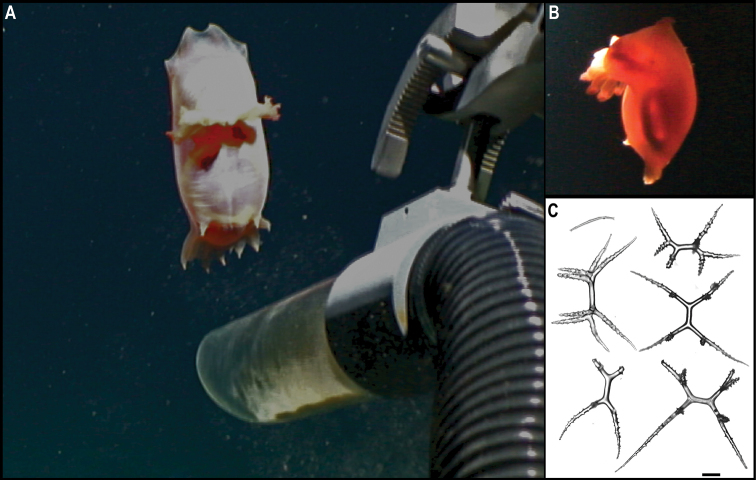
*Peniagoneleander* Pawson & Foell, 1986. Specimen CCZ_018: **A, B** in situ images **C** tentacle ossicles. Scale bars: 100 µm (**C**). Image attribution: Durden and Smith (**A, B**); Bribiesca-Contreras (**C**).

Single specimen observed swimming (Fig. 46A). Specimen was severely damaged during collection, with only a few tentacles recovered, and hence description of morphological characters is based on in situ images. Body ovoid, slightly > 2× as long as it is wide. Velum composed of two pairs of fully fused papillae. Tube feet four pairs; three posteriormost pairs fused together forming a posterior swimming lobe; tube feet from the anteriormost pair very short.

###### Remarks.

The specimen collected during the DeepCCZ expedition was recovered in bits, so no morphological features can be distinguished. Only four reddish orange tentacles were recovered, which are embedded in a transparent skin where ossicles are evident. However, *P.leander* is one of the few species that can be identified from images. The external morphological characters evident in in situ images from the CCZ specimen are in accordance with the species description. The species was originally described from in situ images and video footage collected across the eastern CCZ (Pawson and Foell 1986) and subsequently observed in the area (e.g., Amon et al. 2017b).

In the phylogenetic tree, the CCZ specimen was recovered in a well-supported clade with other species of *Peniagone* (Fig. 34). It was recovered together with a sequence of *P.leander*, which was recently rediscovered and collected for the first time in the Mariana Trench (Gong et al. 2020), both close to *P.diaphana* as reported by Gong et al. (2020). The 16S sequence of the CCZ specimen is similar (K2P distance = 2%) to the only available sequence from *P.leander*, but no COI sequence was made available. Our COI sequence is > 12% divergent (K2P distance) from other species within the genus. The COI gene seems to be highly divergent between species in this genus. Using the data provided in Kremenetskaia et al. (2021) and including the CCZ sequence of *P.leander*, COI mean interspecific divergence in the genus is 15.9% (min = 2.5% and max = 22.7%), with our sequence of *P.leander* being 14.5%–21.2% divergent from other species within the genus. Intraspecific divergence for species in the genus was estimated between 0.9%–3.0%.

###### Ecology.

The specimen was found swimming near the sediment surface on an abyssal plain in APEI 7 at 4860 m depth.

###### Comparison with image-based catalogue.

*Peniagoneleander* (HOL_028) has been commonly encountered in seabed image surveys conducted across the eastern CCZ (e.g., Amon et al. 2017b) and in abyssal areas of the Kiribati EEZ, usually swimming above the seabed but sometimes creeping on it. Body colour appears to be variable; bright red, semi-transparent, purplish, and whitish HOL_028 specimens have been encountered in seabed image surveys across the CCZ.

##### ﻿ Peniagone vitrea

Taxon classificationAnimaliaElasipodidaElpidiidae

Théel, 1882

ACBB0DC0-8668-58E1-94B0-3D06DBF0B370

Fig. 47

###### Material.

Clarion-Clipperton Zone • 1 specimen; APEI 7; 5.0442°N, 141.8164°W; 4875 m deep; 28 May. 2018; Smith & Durden leg.; GenBank: ON400699 (COI), ON406622 (16S); NHMUK 2022.64; Voucher code: CCZ_077.

###### Other material.

Pacific Ocean • 1 specimen, syntype of Peniagonevitreavar.setosa Ludwig; South Pacific; 0.6°S, 86.7667°W; 2418 m deep; Albatross Expedition; NHMUK 1895.11.12.7. • 3 specimens, syntypes of *Peniagonevitrea* Théel, 1882; East of St. Paul, Indian-Antarctic Ridge; 42.7167°S, 82.1833°W; 2652 m deep; Challenger Expedition, Stn. 302; NHMUK 1883.6.18.82.

###### Description.

**Figure 47.**
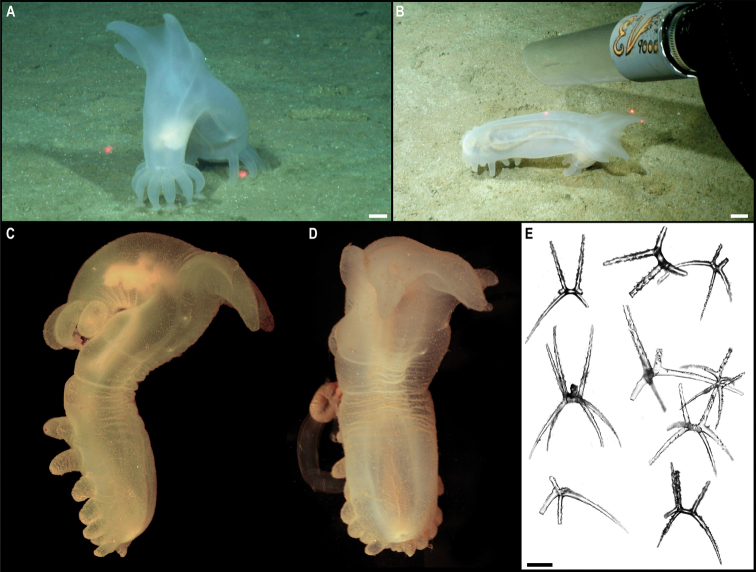
*Peniagonevitrea* Théel, 1882. Specimen CCZ_077: **A, B** in situ images **C** lateral view before preservation **D** dorsal view **E** dorsal ossicles. Scale bars:2 cm (**A**); 3 cm (**B**); 200 μm (**E**). Image attribution: Durden and Smith (**A, B**); Wiklund, Durden, Drennan, and McQuaid (**C, D**); Bribiesca-Contreras (**E**)

Single specimen. Body long, ~ 3× as long as wide (Fig. 47C, D). Mouth anterior, downwards; foremost neck-like part bent forwards in acute angle with ventral surface (Fig. 47C); with ten tentacles of similar sizes; anus terminal. Velum consists of two pairs processes, fully fused by a membrane forming a lobe, with only the tips free; the two middle processes are much larger (Fig. 47A, B, D). Eight pairs of tube feet surrounding the posterior third of ventral surface, decreasing in size distally. Skin translucent in live specimen (Fig. 47A, B), but white, hard, and brittle after preservation, with numerous calcareous deposits (Fig. 47C, D). Dorsal ossicles with four spinose arms, slightly arched, with mostly two long spinose processes (Fig. 47E).

###### Remarks.

Morphological external characters and ossicle morphology are in accordance with the original description of *Peniagonevitrea*. Unfortunately, no genetic sequences of *P.vitrea* are available in public databases. This species was described from off Patagonia at 2652 m depth. Using data from Kremenetskaia et al. (2021), the COI sequence of *P.vitrea* is 16.5%–18.8% divergent (K2P) from other species of *Peniagone*, and 17.9% divergent from the COI sequence of *P.leander* generated in this study. In the phylogenetic tree, it is recovered in a well-supported clade with other species of *Peniagone* (Fig. 34).

###### Ecology.

The specimen was found feeding on the sedimented seafloor of an abyssal plain in APEI 7 at 4874 m.

###### Comparison with image-based catalogue.

A very similar *Peniagone* sp. morphotype (i.e., *Peniagonevitrea* sp. inc., HOL_059) has been commonly encountered in seabed image surveys conducted across nodule fields areas of the eastern CCZ (e.g., Amon et al. 2017b), but not in the abyssal areas surveyed within the Kiribati EEZ.

**Family Laetmogonidae Ekman, 1926**

#### Genus *Psychronaetes* Pawson, 1983

##### ﻿ Psychronaetes

Taxon classificationAnimaliaElasipodidaLaetmogonidae

sp. CCZ_101

2DF2853B-29E7-5CB4-BDC2-7EBB65A1289B

Fig. 48

###### Material.

Clarion-Clipperton Zone • 1 specimen; APEI 7; 4.8877°N, 141.757°W; 3132 m deep; 27 May. 2018; Smith & Durden leg.; GenBank: ON400690 (COI), ON406630 (18S); NHMUK 2022.62; Voucher code: CCZ_063. • 1 specimen; APEI 4; 7.2647°N, 149.7741°W; 3562 m deep; 03 Jun. 2018; Smith & Durden leg.; GenBank: ON400707 (COI), ON406631 (18S); NHMUK 2022.65; Voucher code: CCZ_101. • 1 specimen; APEI 4; 7.2647°N, 149.7741°W; 3562 m deep; 03 Jun. 2018; Smith & Durden leg.; GenBank: ON400709 (COI), ON406639 (18S); NHMUK 2022.67; Voucher code: CCZ_103. • 1 specimen; APEI 4; 7.2647°N, 149.7741°W; 3562 m deep; 03 Jun. 2018; Smith & Durden leg.; GenBank: ON400710 (COI), ON406632 (18S); NHMUK 2022.68; Voucher code: CCZ_104.

###### Description.

**Figure 48.**
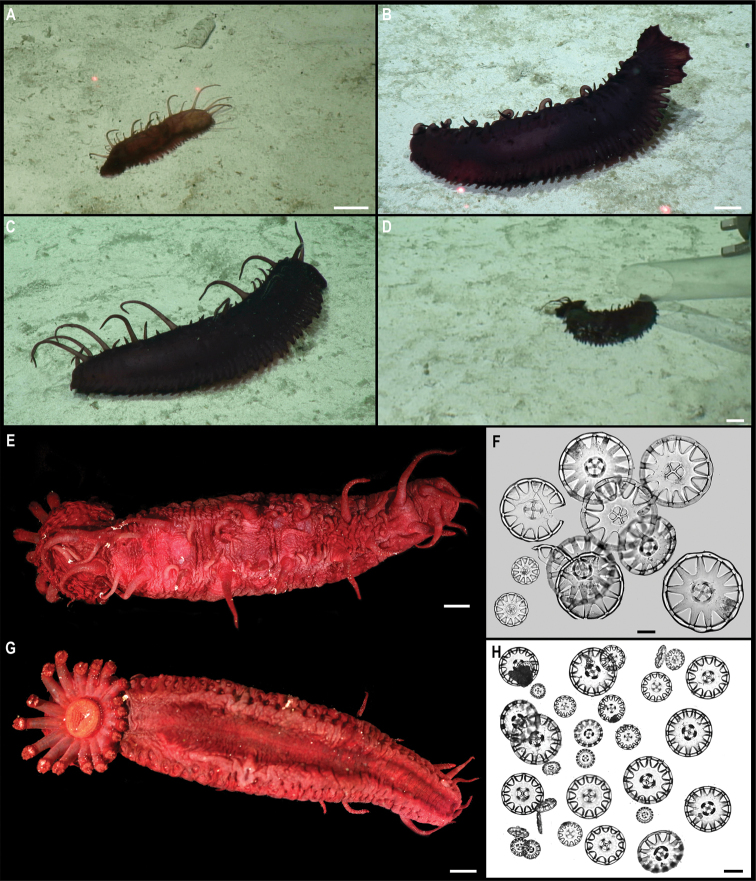
*Psychronaetes* sp. CCZ_101. Specimen CCZ_063 **A** in situ image. Specimen CCZ_101 **B** in situ image **F** dorsal ossicles. Specimen CCZ_103 **C** in situ image **H** dorsal ossicles. Specimen CCZ_104 **D** in situ image **E** dorsal view of specimen before preservation **G** ventral view. Scale bars: 5 cm (**A, D**); 2 cm (**B**); 1 cm (**E, G**); 75 μm (**F**); 100 μm (**H**). Image attribution: Durden and Smith (**A–D**); Wiklund, Durden, Drennan, and McQuaid (**E, G**); Bribiesca-Contreras (**F, H**).

Four specimens (Fig. 48A–D). Body dorso-ventrally flattened (L = 15.9 cm, W = 3.4 cm), tapering at both ends; pronounced “neck” anteriorly; mouth anteroventral, anus posterodorsal. Tentacles 18, with long stems and large elongate oval discs (Fig. 48G). Body wall firm and leathery, dark violet in preserved specimen, with evident, numerous calcareous wheel-like ossicles that make the skin sparkle under light. Mid-ventral ambulacrum is naked; one irregular row of ≥ 40 tube feet running along each ventrolateral ambulacra (Fig. 48G), very conspicuous on live specimens (Fig. 48A–D), but fully retracted on preserved specimens. Paired dorsal ambulacra with ~ 20 papillae each; ~ 7 long thick papillae distributed along each ambulacrum, interspersed with smaller ones (Fig. 48A–E). There are ~ 18 large conical papillae surrounding the anterior margin dorsally, fully fused forming a fringe (Fig. 48B, D). Dorsal ossicles numerous, wheel-like, of different sizes (ranging from 77–340 μm in diameter) but mostly large; strongly concave; central primary cross with 4–6 struts, mostly four; smooth rim; with 10–16, mostly 12, short spokes (Fig. 48F, H).

###### Remarks.

Based on ossicle morphology, the four specimens were considered to belong to the same species. Sequence of the 18S were found to be identical between specimens CCZ_063, CCZ_101, and CCZ_104 (0.0% K2P distance) but 1.3% divergent from CCZ_103. The COI gene was amplified for the four specimens and genetic divergence ranges between 0.8% to 7.4%. The two specimens collected in APEI 4 are less genetically divergent (CCZ_101-CCZ_104 = 0.8% K2P). The specimen collected in APEI 7 (CCZ_063) is 2.3–2.9% divergent from the other three. The specimen CCZ_103 is 7.4% divergent to CCZ_101 and CCZ_104, but only 2.9% divergent from CCZ_063. While the former values are within the range of interspecific genetic divergence, we considered the specimen to belong to the same species as both the ossicle and external morphological characters are similar to the other three specimens. In addition, the trace files for both 18S and COI for this specimen are messy and the high genetic divergence could be an artifact of miss-called nucleotides. Unfortunately, there are no sequences available for *Psychronaetes*, but we included sequences of other genera within the family (*Pannychia*, *Laetmogone*, and *Benthogone*) for which COI genetic divergence ranged from 23–31%. In the phylogenetic tree, the four specimens were recovered in a well-supported clade (Fig. 34), close to other Laetmogonidae (poorly supported). This species has an anterior brim, which is characteristic of the monotypic genus *Psychronaetes. Psychronaeteshanseni* Pawson, 1983 differs from the four specimens in having smaller dorsal wheel ossicles (d = 50–80 µm) with 9–12 spokes, only 15 tube feet on each of paired ventral ambulacrum, 15 mouth tentacles instead of 18, and in the number of papillae on the dorsal paired ambulacra. The species and genus were described from two specimens collected in the CCZ (Pawson 1983).

###### Ecology.

The four specimens were found on the sedimented seafloor of seamounts in APEIs 4 and 7 between 3132–3562 m depth.

###### Comparison with image-based catalogue.

No exactly similar *Psychronaetes* sp. morphotypes have been encountered in seabed image surveys conducted in the eastern CCZ or in abyssal areas of the Kiribati EEZ. Consequently, the in situ images of these specimens were catalogued as a new morphotype (i.e., *Psychronaetes* sp. indet., HOL_110). However, HOL_110 can resemble at least two other Laetmogonidae morphotypes catalogued from seabed imagery; Laetmogonidae gen. indet., HOL_030 (e.g., dark violet, but with 8+ long papillae) which is commonly found in the eastern CCZ (but not in the Kiribati EEZ); and *Psychronaetes* sp. indet., HOL_122 (e.g., violet, but only with six or seven long papillae and with fewer (< 20) and larger, thick tube feet) which was also only found in the western CCZ.

#### Genus *Laetmogone* Théel, 1879

##### ﻿ Laetmogone cf. wyvillethomsoni

Taxon classificationAnimaliaActinopodaLaetmogonidae

Théel, 1979

4EAF8230-AF8B-529E-8962-EB5441589374

Fig. 49

###### Material.

Clarion-Clipperton Zone • 1 specimen; APEI 7; 4.8877°N, 141.7569°W; 3132 m deep; 27 May. 2018; Smith & Durden leg.; GenBank: ON400689 (COI), ON406641 (18S); NHMUK 2021.18; Voucher code: CCZ_062.

###### Other material.

Pacific Ocean • 1 specimen, holotype of *Laetmogonespongiosa* Théel, 1879; south of Japan; 34.1167°N, 138°E; 1033 m deep; Challenger Expedition, Stn. 235; NHMUK 1883.6.18.47.

###### Description.

**Figure 49.**
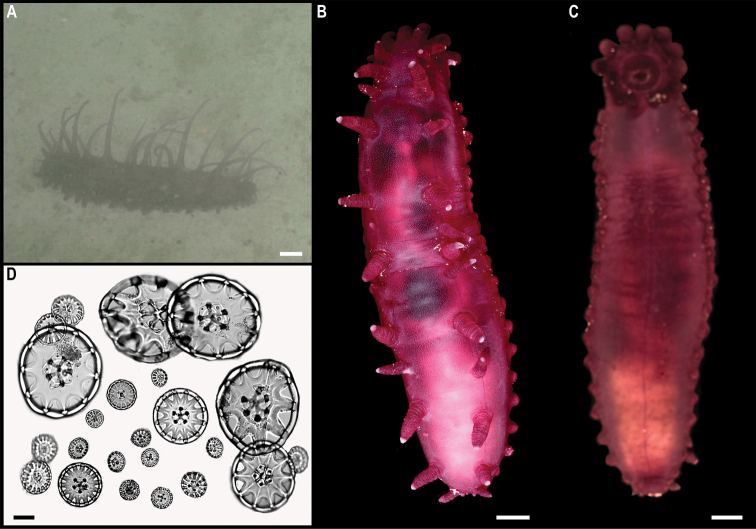
Laetmogonecf.wyvillethomsoni Théel, 1979. Specimen CCZ_062 **A** in situ image; **B** dorsal view of specimen before preservation **C** ventral view **D** dorsal calcareous ossicles. Scale bars: 2 cm (**A**); 1 cm (**B, C**); 50 μm (**D**). Image attribution: Durden and Smith (**A**); Wiklund, Durden, Drennan, and McQuaid (**B, C**); Bribiesca-Contreras (**D**).

Single specimen (Fig. 49A). Body cylindrical, ~ 3× as long as wide (L = 15.6 cm, W = 5.2 cm), with convex dorsal surface and somewhat flattened ventral surface, tapering posteriorly; mouth anterior, subventral, terminal; anus posterior, terminal, slightly dorsal; violet colouration in live and preserved specimen, with darker ventral surface (Fig. 49A–C). Tentacles 15, of almost equal size, and very dark at the tips. Odd ambulacrum is naked; 27 or 28 tube feet arranged in a single row on each of the paired ventral ambulacra, forming a continuous line on the anterior ⅔ of the body and scattered posteriorly, also decreasing in size (Fig. 49C). Each paired dorsal ambulacrum with a single row of long processes, 12 on the left and 13 on the right; longest processes longer than ⅓ of the body length. Twenty pedicels along each side of the ventral surface, posterior pairs smaller than the others. Fourteen processes of the bivium along the left ambulacra and thirteen along the right. Tegument is thick, completely covered by calcareous ossicles. Dorsal ossicles are wheel-like of various sizes (40–226 μm in diameter), with four or five studs, mostly five, on primary central crosses, and with 8–17 spokes, mostly eight on large wheels; ossicle is convex, rim smooth, interspoke areas small, and large central area on large wheels (Fig. 49D).

###### Remarks.

Closest match on public databases for the COI gene sequence was other sequences of *Laetmogonewyvillethomsoni* Théel, 1879 (4.0–5.8% K2P genetic distance) from the Ross Sea and Marie Byrd Seamounts (O’Loughlin et al. 2011). The specimen from the CCZ and specimens from *L.wyvillethomsoni* from Antarctica were recovered in our phylogeny (Fig. 34) in a well-supported clade (Fig. 34), which is subdivided in three clades including the two Antarctic clades stratified by depth reported in O’Loughlin et al. (2011), and the specimen from the CCZ. Type material for *L.wyvillethomsoni* was collected during the H.M.S. Challenger expedition at stations 300 (off the coast of South America; 33.7° S, 78.3°W; 2514 m depth) and 147 (west of the Crozet Islands; 46.2667° S, 48.45° E; 2926 m), and high morphological variability was reported (Théel 1879). The CCZ specimen morphologically resembles *L.wyvillethomsoni*, but no rod-shaped ossicles were found in the dorsal skin, differing from the original description.

###### Ecology.

The specimen was found on the sedimented seafloor of a seamount in APEI 7 at 3132 m depth.

###### Comparison with image-based catalogue.

No similar laetmogonid morphotypes have been encountered in seabed image surveys conducted in the eastern CCZ or in abyssal areas of the Kiribati EEZ. Consequently, the in situ image of specimen CCZ_062 was catalogued as a new morphotype (i.e., *Laetmogone* sp. indet., HOL_123).

**Class Ophiuroidea**

**Figure 50.**
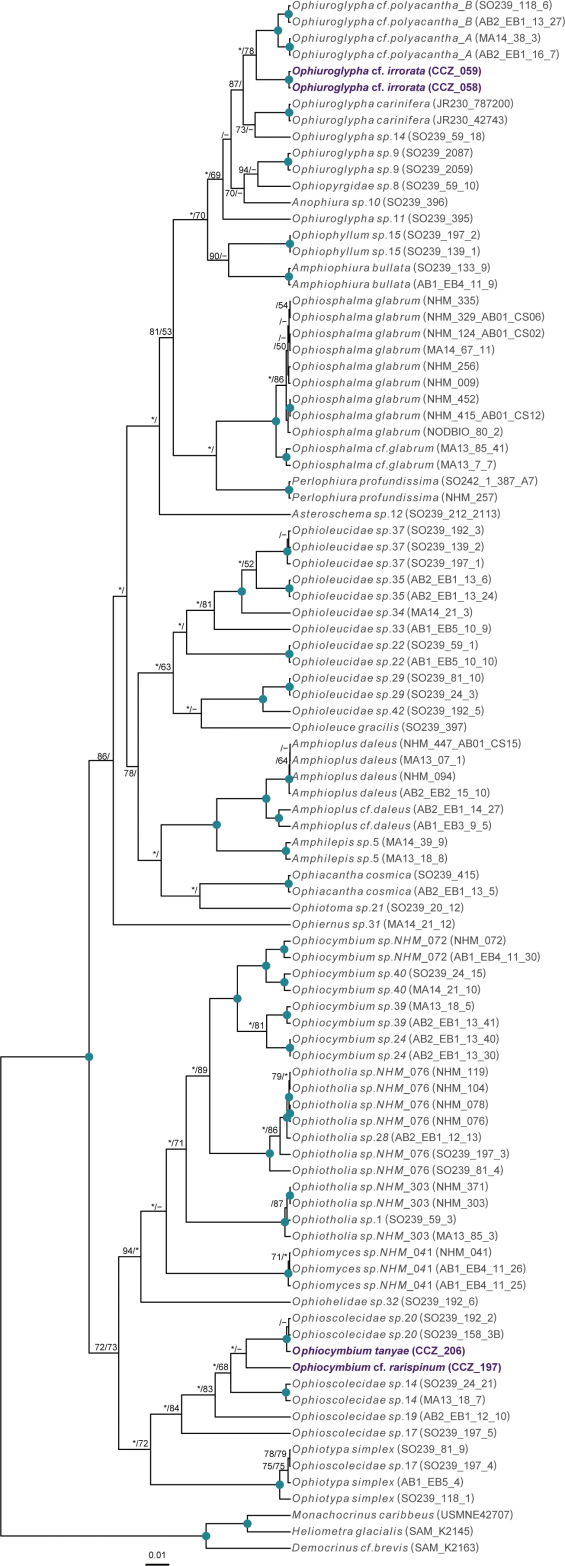
Phylogenetic tree of Ophiuroidea. Concatenated (28S, and COI) median consensus BEAST tree with posterior probability (PP) and bootstrap (BS) values indicated. Only values of PP > 0.70 and BS > 50 are shown, with values of PP > 0.95 and BS > 90 indicated with a circle. Nodes not recovered on the RAxML tree are indicated with a hyphen. Sequences generated in this study are highlighted in violet.

To date, there are 1201 records of ophiuroids occurring at > 3000 m depth in the CCZ, with 117 representing preserved specimens (OBIS 2022). Four specimens belonging to three different species were collected and the barcoding gene COI was amplified for all but one, for which both 18S and 28S were amplified. These sequences, excluding 18S, were included in a concatenated alignment (28S, and COI) and used to estimate a phylogenetic tree (Fig. 50). Ophiuroidea is amongst the most challenging groups to identify and classify based on seabed image data only; key morphological features are too small to be appropriately visualised (e.g., plates and scales) and/or are found on the ventral disc (not visible in images). As a result, the taxonomic resolution of ophiuroid morphotypes catalogued from seabed imagery is usually much lower than that in other echinoderm groups. Consequently, connectivity and distribution patterns of ophiuroids derived from seabed image data should be interpreted cautiously.

**Subclass Myophiuroidea Matsumoto, 1915**

**Infraclass Metophiurida Matsumoto, 1913**

**Superorder Ophintegrida O’Hara, Hugall, Thuy, Stöhr & Martynov, 2017**

**Order Ophioscolecida O’Hara, Hugall, Thuy, Stöhr & Martynov, 2017**

**Family Ophioscolecidae Lütken, 1869**

#### Genus *Ophiocymbium* Lyman, 1880

##### ﻿ Ophiocymbium tanyae

Taxon classificationAnimaliaOphioscolecidaOphioscolecidae

Martynov, 2010

362E33D7-6308-5457-B57D-3BE76EAFB1AB

Fig. 51

###### Material.

Clarion-Clipperton Zone • 1 specimen; APEI 1; 11.2523°N, 153.5848°W; 5204 m deep; 10 Jun. 2018; Smith & Durden leg.; GenBank: ON406633 (18S), ON406596 (28S); NHMUK 2022.74; Voucher code: CCZ_206.

###### Description.

**Figure 51.**
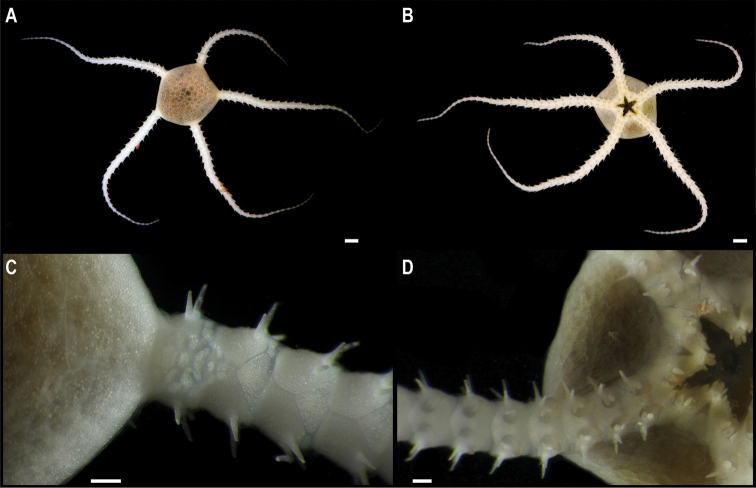
*Ophiocymbiumtanyae* Martynov, 2010 **A** dorsal view of specimen CCZ_206 before preservation **B** ventral view **C** detail of dorsal disc surface and dorsal arm plates **D** detail of jaws, ventral disc surface and ventral arm plates. Scale bars: 2 cm (**A, B**); 5 mm (**C, D**). Image attribution: Wiklund, Durden, Drennan, and McQuaid (**A–D**).

Single specimen (disc diameter = 9 mm, maximum arm length = 25 mm). Disc subpentagonal, flattened (Fig. 51A, B). Dorsal disc surface covered with numerous, imbricated, delicate disc scales, which are irregular in shape, decrease in size distally and extend dorsally onto the first arm segments (Fig. 51C). Radial shields and genital plates apparently absent. Disc covered by thin skin, not obscuring the scales. Ventral surface of the disc covered by scales similar to the dorsal disc scales (Fig. 51D). Oral shield somewhat triangular, approx. as long as wide, with convex distal edge; separated from first lateral arm plate by the adoral shields. Adoral shields are wing-shaped, narrowing proximally. Each jaw bears a large, spiniform, apical papillae and a smaller adjacent one on each side; additionally, there are two to three modified papillae placed distally on each side of the jaws, block-shaped, the distalmost being wing-shaped. Genital slit is not conspicuous. Arms are thin, longer than twice the disc diameter (Fig. 51A, B). Dorsal arm plates are triangular, wider than long, with pointed proximal and straight distal edges; separated by lateral arm plates and therefore not overlapping with preceding dorsal arm plate (Fig. 51C). Arm spines are conical, tapering distally but with rounded tips; two arm spines on first three arm segments, three arm spines on next three segments and four on the rest; middle arm spine is the longest, but all are approx. the same length, approx. half the length of one arm segment. First ventral arm plate is triangular, while the rest are pole-axe shaped, approx. as long as wide, separated from the preceding plate by the lateral arm plates except for the first two ventral arm plates (Fig. 51D). Tentacle pores are large and evident throughout the entire length of the arm. Three flattened, rounded, large adoral shield papillae. First four arms segments with two large papilliform tentacle scales attached to the lateral arm plate; subsequent arm segment with a single tentacle scale; tentacle scales absent on the remaining arm segments.

###### Remarks.

Morphological characters of the specimen are in accordance with the description of *O.tanyae*, which was collected in the Izu-Bonin Trench at 6740–6850 m depth. It differs from the original description in having arms ≥ 2× as long as the disc diameter (dd), instead of being approx. the same. It also differs on the tentacle scales, which extend to the fifth segment, instead of just the third, having two tentacle scales in the first four segments instead of just one, and in the number of arm spines of the first arm segments. The number of arm spines is discussed to vary amongst the paratypes (Martynov 2010), and it is very likely that tentacle scales are easily lost and therefore the number could differ between specimens. Only 18S and 28S were amplified for this specimen. The 28S sequence of the CCZ specimen is identical (K2P = 0%) to the sequence of the species Ophioscolecidae sp. 20 recently reported for the CCZ (Christodoulou et al. 2020). Both specimens are recovered within the same clade, that includes other species of the order Ophioscolescida (Fig. 50). Ophioscolescida sp. 20, *Ophiocymbiumtanyae*, and *O.rarispinum* Martynov, 2010 are recovered as a clade possibly representing the genus *Ophiocymbium*. The species from Christodoulou et al. (2020) was identified from DNA sequences only, as the four specimens collected (eastern IFREMER and APEI 3) are tiny juveniles with no distinctive morphological characters. The species is therefore distributed in the Izu-Bonin Trench and the Clarion-Clipperton Zone.

###### Ecology.

The specimen was found on the sedimented seafloor of an abyssal plain on APEI 1 at 5204 m depth.

##### ﻿ Ophiocymbium cf. rarispinum

Taxon classificationAnimaliaOphioscolecidaOphioscolecidae

Martynov, 2010

90646AB6-1F16-5E90-A4DC-B58E0252598A

Fig. 52

###### Material.

Clarion-Clipperton Zone • 1 specimen; APEI 1; 11.2518°N, 153.6059°W; 5206 m deep; 10 Jun. 2018; Smith & Durden leg.; GenBank: ON400727 (COI); NHMUK 2022.73; Voucher code: CCZ_197.

###### Description.

**Figure 52.**
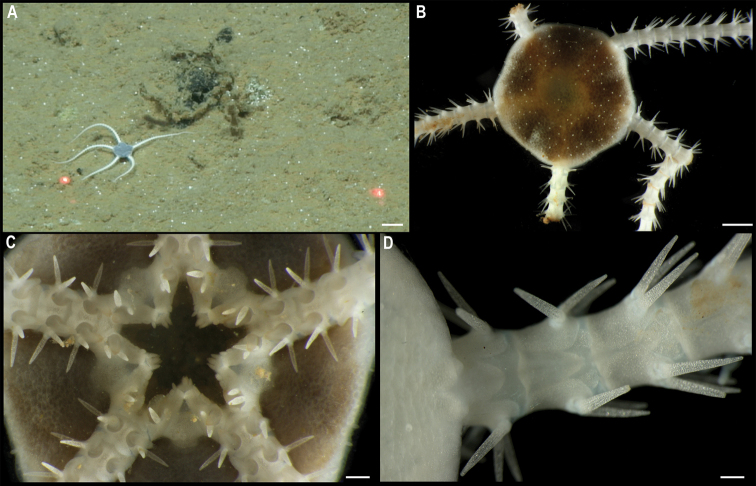
Ophiocymbiumcf.rarispinum Martynov, 2010. Specimen CCZ_197 **A** in situ image **B** dorsal surface before preservation **C** detail of ventral surface, jaws and ventral arm plates **D** detail of dorsal arm plates. Scale bars: 1 cm (**A, B**); 5 mm (**C**); 2.5 mm (**D**). Image attribution: Durden and Smith (**A**); Wiklund, Durden, Drennan, and McQuaid (**B–D**).

Single specimen, with white arms and greyish blue disc in situ (Fig. 52A). Disc is flattened and somewhat pentagonal; brownish when alive and white after preservation (Fig. 52B, D). Dorsal disc surface is covered by minute, thin, imbricated scales covered by a thin skin, not obscuring the scale margins; few granuliform spinelets scattered on the dorsal surface (Fig. 52B). Small, oval, radial shields, approx. as long as wide, arranged diagonally and touching proximally; distal margin extends beyond the disc margin (Fig. 52D). Ventral surface of the disc covered by scales similar to the ones on the dorsal surface, but lacking spinelets; gonads are visible through the thin scales (Fig. 52C). Each jaw bears two or three apical papillae, and three oral papillae on each side; two distalmost are block-shaped while the third distalmost is spiniform. Oral shield is triangular, longer than wide, rounded proximally; separated from the first lateral arm plate by the wing-shaped adoral shield. Supplementary oral shield, wider than long, located on the distal margin of the oral shield. Two adoral shield papillae, with one placed in the middle of each shield, resembling arm spines in shape and size. Arms are slender, ≥ 3× as long as the disc diameter. Dorsal arm plates triangular, with rounded distal margin and slightly convex distal edge, separated from preceding plates by lateral arm plates; first two dorsal arm plates absent but arm segments covered by thick skin with smaller plates embedded (Fig. 52D). Adjacent lateral arm plates are slightly separated by soft tissue; each lateral arm plate bears four long arm spines, similar in size; only two arm spines present in the first three arm segments and three on subsequent two arm segments. First ventral arm plate is small, broad and triangular, with the rest being pole-axe shaped and separated from the preceding plate except for the first two segments. Tentacle pores are large throughout the entire length of the arm, with no tentacle scales except for the first arm segment, where there is one attached to the lateral arm plate and resembles a small arm spine.

###### Remarks.

In the phylogenetic tree, the specimen from the western CCZ is recovered as closely related to *Ophiocymbiumtanyae* (Fig. 50). *Ophiocymbiumrarispinum* was described from the Izu-Bonin Trench, between 6740 and 6850 m depth (ZMMU D-798), and no genetic sequences have been published. Morphological characters are concordant with the description of *O.rarispinum*, but differs in the length of the arms, the number of oral papillae and the number of arm spines.

###### Ecology.

The specimen was found crawling on the abyssal sediments of APEI 1 at 5206 m depth.

###### Comparison with image-based catalogue.

A similar Ophiuroidea morphotype (i.e., *Ophiocymbium* sp. indet., OPH_013) has been encountered in seabed image surveys conducted across nodule fields areas of the eastern CCZ, but not in the abyssal areas surveyed within the Kiribati EEZ.

**Superorder Euryophiurida O’Hara, Hugall, Thuy, Stöhr & Martynov, 2017**

**Order Ophiurida Müller & Troschel, 1840 sensu O’Hara et al. 2017**

**Suborder Ophiurina Müller & Troschel, 1840 sensu O’Hara et al. 2017**

**Family Ophiopyrgidae Perrier, 1893**

#### Genus *Ophiuroglypha* Hertz, 1927

##### ﻿ Ophiuroglypha cf. irrorata

Taxon classificationAnimaliaOphiuridaOphiopyrgidae

(Lyman, 1878)

07FCEAB9-BF5D-596C-9813-8C6015EEB01E

Fig. 53

###### Material.

Clarion-Clipperton Zone • 1 specimen; APEI 7; 4.9081°N, 141.6813°W; 3239 m deep; 26 May. 2018; Smith & Durden leg.; GenBank: ON400685 (COI); NHMUK 2021.21; Voucher code CCZ_058. • 1 specimen; APEI 7; 4.8897°N, 141.75°W; 3096 m deep; 27 May. 2018; Smith & Durden leg.; GenBank: ON400686 (COI); NHMUK 2022.72; Voucher code: CCZ_059.

###### Description.

**Figure 53.**
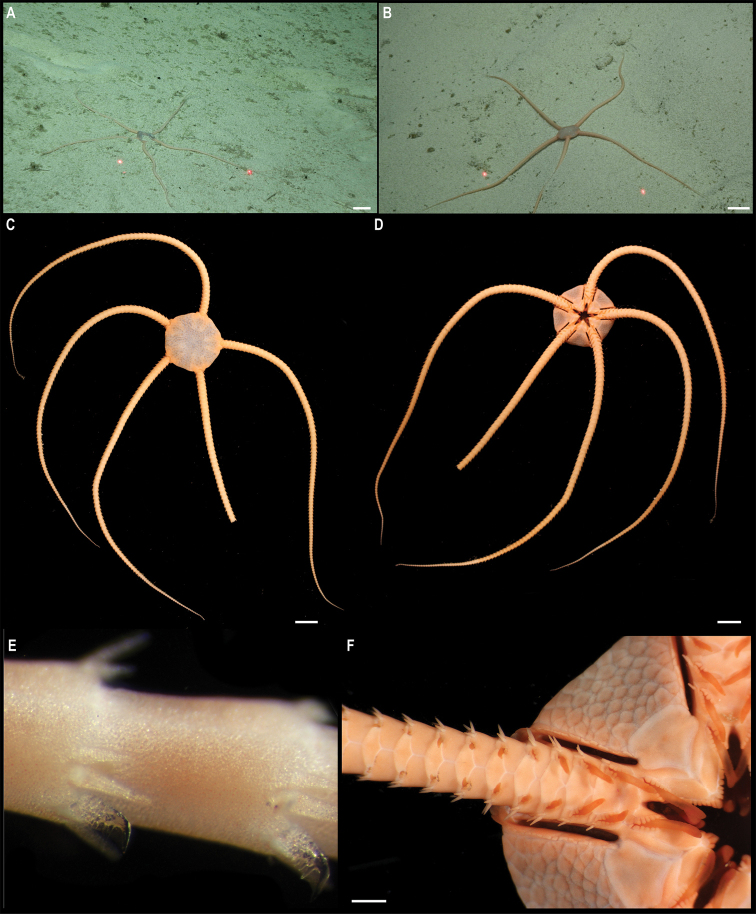
Ophiuroglyphacf.irrorata (Lyman, 1878). Specimen CCZ_059 **A** in situ image. Specimen CCZ_058 **B** in situ image **C** dorsal view of specimen before preservation **D** ventral view **E** arm hooklets **F** detail of ventral disc surface and ventral arm plates. Scale bars: 2 cm (**A, B**); 1 cm (**C, D**); 2 mm (**F**). Image attribution: Durden and Smith (**A, B**); Wiklund, Durden, Drennan, and McQuaid (**C–F**).

Two specimens, with greyish disc and pale arms in situ (Fig. 53A, B). Disc rounded to pentagonal, flattened, with slender, long arms, at least disc diameter (disc diameter = 2.6 cm, arm length = 13.1 cm; Fig. 53C, D). Dorsal disc surface covered by irregular, larger disc scales surrounded by small, imbricated disc scales that also vary in size and shape. Radial shields are small, subtriangular, almost as wide as long. Arm combs visible under the radial shields; with five short block-like arm-comb spinelets, not continuous on dorsal midline. Ventral surface of the disc is covered by large, imbricated scales, increasing in size towards the margin of the disc (Fig. 53F). Oral plates with 6–8 oral papillae, proximalmost are pointed, becoming block-like towards the distal side of the oral plate. Oral shield approx. as long as wide, subpentagonal, with somewhat concave proximal margins, a convex distal margin, and with lateral margins slightly constricted in the middle, where the genital slit begins. Adoral shields touching proximally, with a similar width all along, and separating the oral shield from the first lateral arm plates. Genital slits run from the middle of the oral shield to the disc margin, bordered by a continuous row of block-like genital papillae that continues dorsally as an arm-comb.

Dorsal arm plates fan-shaped, contiguous. Lateral arm plates bear three short (less than a third of the length of the arm segment) arm spines from the third arm segment; two are located ventrally, very close together, and one located dorsally, approx. halfway through the lateral arm plate; first arm segment bears two arm spines, the second two or three spines. Ventral arm plate trapezoidal, wider than long, only touching the preceding plate only on first three arm segments, after which they are separated by the lateral arm plates and become fan-shaped to rhomboidal, more than twice as wide as long, with pointed proximal edge and rounded distal margin. Towards the distal end of the arms, the second lowest spine is modified into a hyaline hooklet (Fig. 53E). Tentacle pores only on most proximal segments (8–11), with six ventral and six lateral tentacle scales on first arm segment and decreasing in number until there is a single, very small, spiniform, tentacle scale remaining for most of the arm length.

###### Remarks.

Both specimens collected are only 0.4% divergent (K2P distance) in COI sequences between them. Closest genetic match is *Ophiuroglypha* sp. (8% K2P distance) collected in the CCZ (Christodoulou et al. 2020), and in the phylogenetic tree they were recovered in a well-supported clade along with other species of *Ophiuroglypha* (Fig. 50). Both specimens have an upturned hook in the second lowest arm spine, which is characteristic of species of the genus *Ophiuroglypha* (previously a subgenus but raised to genus by O’Hara et al. (2018)). Morphologically, the species resembles to *Ophiuroglyphairrorataconcreta* (Koehler, 1901) based on the arm spine arrangement, dorsalmost spine separated from the two ventral spines. However, the DeepCCZ specimens are listed as O.cf.irrorata, as a recent study suggested that the arm spine arrangement might not be species specific, hence questioning the validity of *O.irroratairrorata* (Lyman, 1878) and *O.irrorataconcreta* (Stöhr and O’Hara 2021). Additionally, molecular data has suggested that *O.irrorata* represents an unresolved complex of species (Christodoulou et al. 2019).

###### Ecology.

Both specimens were found on the sedimented seafloor of a seamount in APEI 7, at 3096 (specimen CCZ_059) and 3239 m (specimen CCZ_058) depth.

###### Comparison with image-based catalogue.

No similar Ophiuroidea morphotypes have been encountered in seabed image surveys conducted in the eastern CCZ nor in abyssal areas of the Kiribati EEZ. Consequently, the in situ images of CCZ_058 and CCZ_059 were catalogued as a new morphotype (i.e., *Ophiuroglypha* sp. indet., OPH_012).

**Phylum Porifera Grant, 1836**

**Figure 54.**
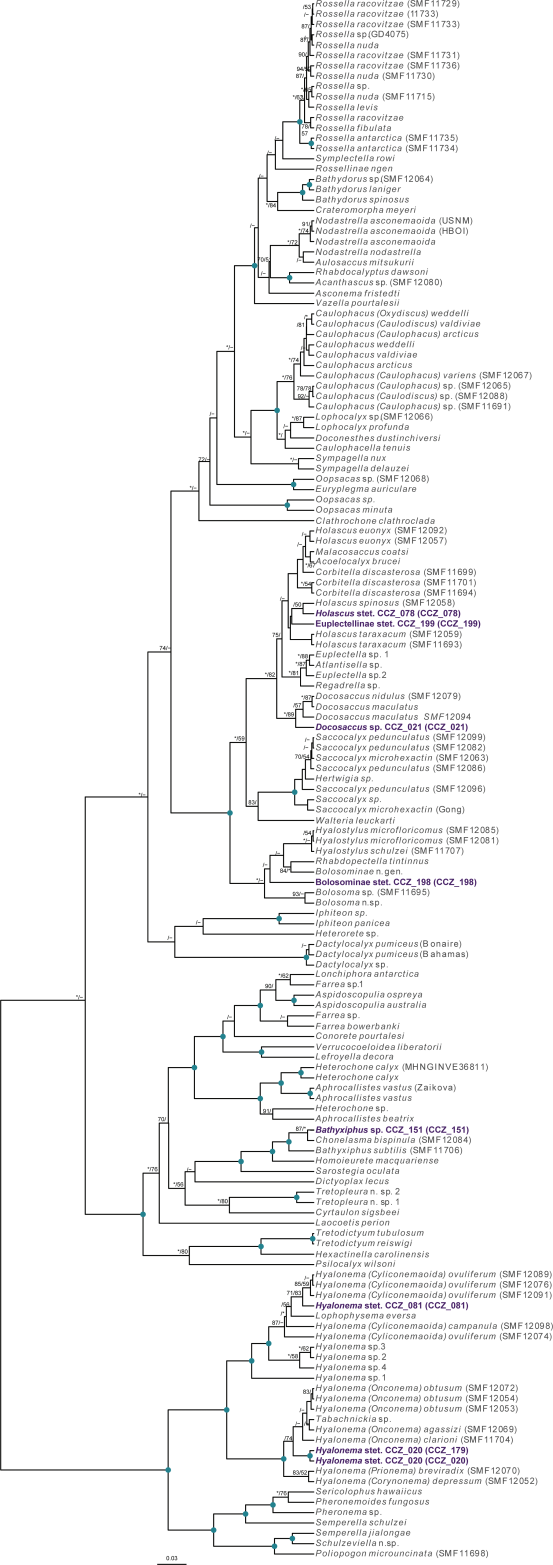
Phylogenetic tree of Hexactinellida. Concatenated (16S, 18S, 28S, and COI) median consensus BEAST tree with posterior probability (PP) and bootstrap (BS) values indicated. Only values of PP > 0.70 and BS > 50 are shown, with values of PP > 0.95 and BS > 90 indicated with a circle. Nodes not recovered on the RAxML tree are indicated with a hyphen. Sequences generated in this study are highlighted in violet.

A total of eight sponges was collected in the western CCZ. All these belong to the class Hexactinellida and represent seven different species, but none was confidently assigned to any known species. To date, there are 255 records of hexactinellid sponges occurring at > 3000 m depth in the CCZ, with only eight representing preserved specimens (OBIS 2022). Several genes were targeted for amplification, but only 16S was successfully amplified for all of them. Other genes amplified were COI (7 specimens), 18S (5), 28S (5), and ALG11 (3). Sequences of these genes were combined with the concatenated alignment from Dohrmann (2018), and the phylogenetic tree was estimated using the same parameters (Fig. 54).

**Class Hexactinellidae Schmidt, 1870**

**Subclass Amphidiscophora Schulze, 1886**

**Order Amphidiscosida Schrammen, 1924**

**Family Hyalonematidae Gray, 1857**

#### Genus *Hyalonema* Gray, 1832

##### ﻿ Hyalonema

Taxon classificationAnimaliaAmphidiscosidaHyalonematidae

stet. CCZ_020

0AB8C416-30A6-508E-92CA-2102141B4806

Fig. 55

###### Material.

Clarion-Clipperton Zone • 1 specimen; APEI 7; 5.1149°N, 141.8967°W; 4856 m deep; 25 May. 2018; Smith & Durden leg.; GenBank: ON400683 (COI), ON406634 (18S), ON406608 (16S), ON406597 (28S), ON411254 (ALG11); NHMUK; Voucher code: CCZ_020. • 1 specimen; APEI 1; 11.2954°N, 153.7422°W; 5245 m deep; 09 Jun. 2018; Smith & Durden leg.; GenBank: ON400721 (COI), ON406609 (16S); NHMUK 2022.8; Voucher code: CCZ_179.

###### Description.

**Figure 55.**
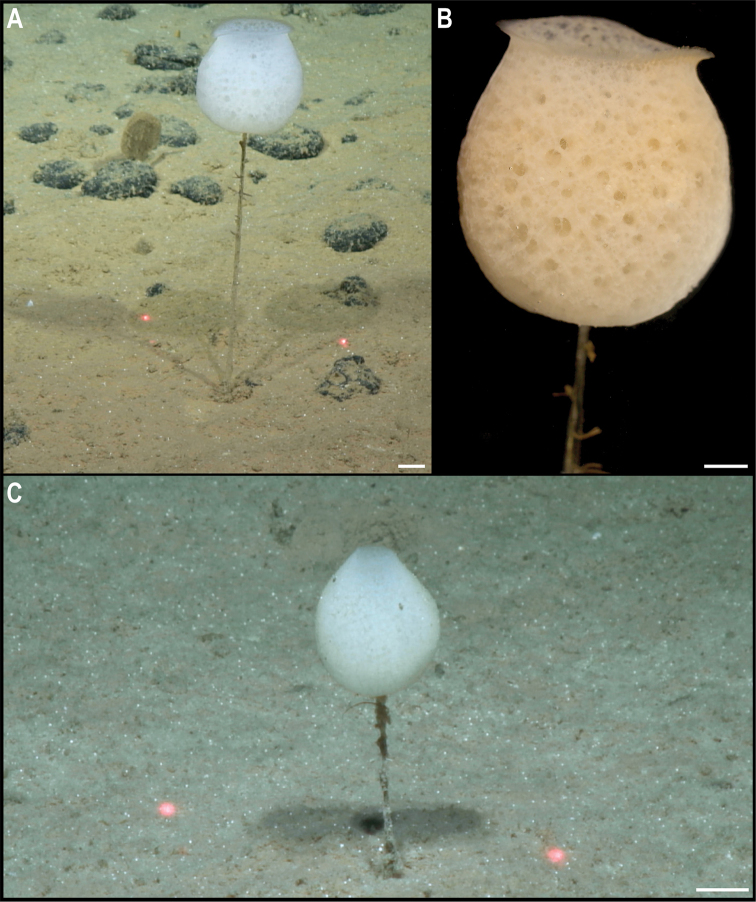
*Hyalonema* stet. CCZ_020. Specimen CCZ_179 **A** in situ image **B** specimen before preservation. Specimen CCZ_020 **C** in situ image. Scale bars: 2 cm (**A, C**); 1 cm (**B**). Image attribution: Durden and Smith (**A, C**); Wiklund, Durden, Drennan, and McQuaid (**B**).

Two specimens. Lophophytous sponges (Fig. 55A–C). Body is white, ovoid, almost as wide as long (CCZ_020 W = 4.7 cm, L = 5 cm; CCZ_179 W = 8 cm, L = 8 cm); with an osculum (CCZ_020 d = 1 cm; CCZ_179 d = 4 cm) surrounded by a thin margin; separated atrial cavity. Attached to the sediment by a thin tuft of basalia that extends from the central lower body (CCZ_020 L > 7 cm; CCZ_179 L > 20 cm).

###### Remarks.

Genetic sequences between specimens CCZ_020 and CCZ_179 are 1% and 0.8% divergent (K2P distance) for COI and 16S, respectively. COI and 16S closest matches are sequences from *Tabachnickia* sp. within the Hyalonematidae. The sequence for the 18S is > 95% similar to other species of *Hyalonema*. In the phylogenetic tree, both specimens were recovered together, in a well-supported clade with other members from different subgenera within the genus *Hyalonema* and including *Tabachnickia* sp. (Fig. 54).

###### Ecology.

The specimens were collected attached to abyssal sediments of APEI 7 and APEI 1 at 4856 and 5245 m depth, respectively.

###### Comparison with image-based catalogue.

A very similar hyalonematid morphotype (i.e., *Hyalonema* sp. indet., HEX_002) has been commonly encountered in seabed image surveys conducted across the eastern CCZ and in abyssal areas of the Kiribati EEZ, mostly in nodule field areas. In situ images of HEX_002 (Fig. 55A, C) show that the aperture width of the central osculum is an unreliable character to distinguish different *Hyalonema* sp. morphotypes (nor these from other genera) in seabed imagery, as this contracts and expands episodically (e.g., Kahn et al. 2020).

**Figure 56.**
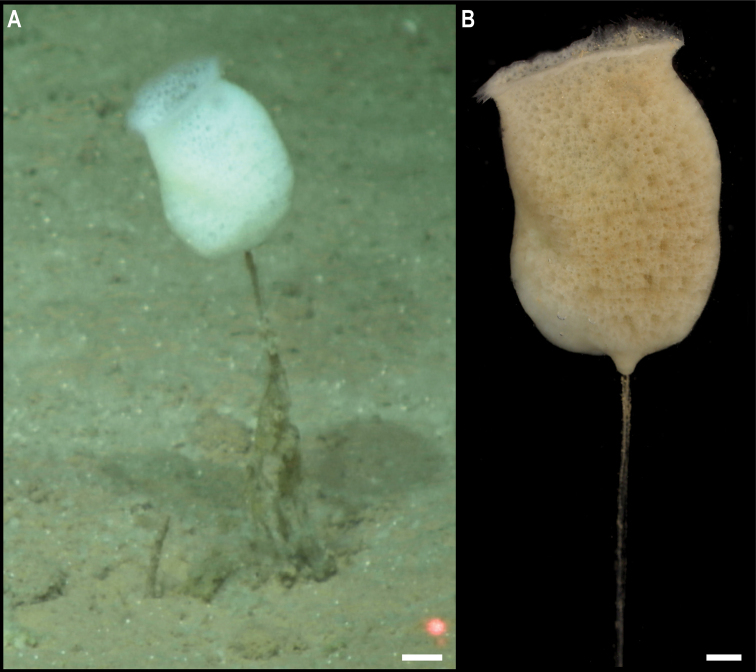
*Hyalonema* stet. CCZ_081 **A** in situ image, **B**. Scale bars: 1 cm (**A**); 5 mm (**B**). Image attribution: Durden and Smith (**A**); Wiklund, Durden, Drennan, and McQuaid (**B**).

##### ﻿ Hyalonema

Taxon classificationAnimaliaAmphidiscosidaHyalonematidae

stet. CCZ_081

2EA9AF49-1BF7-52A8-8881-6E82E6D1B3E9

Fig. 56

###### Material.

Clarion-Clipperton Zone • 1 specimen; APEI 4; 7.036°N, 149.9395°W; 5031 m deep; 01 Jun. 2018; Smith & Durden leg.; GenBank: ON406610 (16S); NHMUK 2022.9; Voucher code: CCZ_081.

###### Description.

Single specimen (Fig. 56). Lophophytous sponge; body white, somewhat bowl-shaped, longer than wide (L = 3.9 cm, W = 2.8 cm); with a wide osculum (d = 2.6 cm) surrounded by a thin margin. Attached to the sediment by a thin tuft of basalia that extends from the central lower body.

###### Remarks.

Morphological characters were found concordant with those of the genus *Hyalonema*. The 16S sequence is very similar (99.34%) to sequences from H. (Cyliconemaoida) ovuliferum Schulze, 1899, being the closest match on public databases. It is recovered in a well-supported clade along with other hyalonematids (Fig. 54), supporting its placement within the genus. It was not possible to assign it any subgenus, as the different subgenera have not been recovered as monophyletic (Dohrmann 2018).

###### Ecology.

This specimen was collected anchored to the sediment on the abyssal plain of APEI 4 at 5031 m.

###### Comparison with image-based catalogue.

A very similar hyalonematid morphotype (i.e., *Hyalonema* sp indet., HEX_003) has been commonly encountered in seabed image surveys conducted across the eastern CCZ and in abyssal areas of the Kiribati EEZ. As observed in HEX_002, the aperture width of the central osculum in HEX_003 can vary owing to body contractions or expansions (Kahn et al. 2020), and should hence not be used to guide identifications of these morphotypes based on seabed imagery.

**Subclass Hexasterophora Schulze, 1886**

**Order Lyssacinosida Zittel, 1877**

**Family Euplectellidae Gray, 1867**

**Subfamily Euplectellinae Gray, 1867**

**Euplectellinae stet. CCZ_199**

Fig. 57

**Material.** Clarion-Clipperton Zone • 1 specimen; APEI 1; 11.2518°N, 153.5853°W; 5202 m deep; 10 Jun. 2018; Smith & Durden leg.; GenBank: ON400729 (COI), ON406611 (16S); NHMUK; Voucher code: CCZ_199.

**Figure 57.**
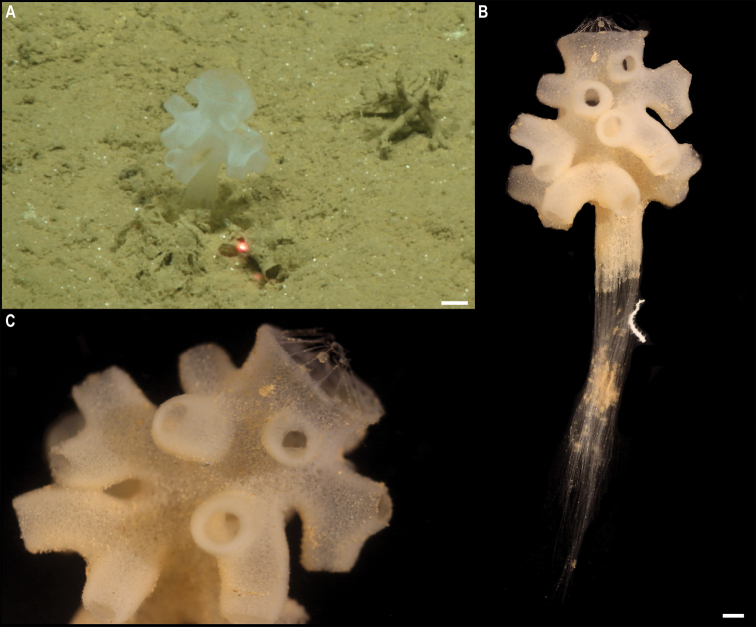
Euplectellinae stet. CCZ_199 **A** in situ image **B** detail of body **C** whole specimen with protruding basalia. Scale bars: 1 cm (**A**); 5 mm (**B**). Image attribution: Durden and Smith (**A**); Wiklund, Durden, Drennan, and McQuaid (**B, C**).

**Description.** Single specimen (Fig. 57A). Lophophytous white sponge with tubular habitus (L = 5 cm). There is a large, central osculum (d = 2cm), and protruding fistules with terminal suboscula, each with one or two, mostly two, openings (Fig. 57B). Long basalia (L = 6 cm) arranged as a tube, protruding from the lower end of the habitus and anchored to the sediment (Fig. 57C).

**Remarks.** The 16S sequence is close to *Corbitelladiscasterosa* Tabachnick & Lévi, 2004 (2.3% K2P distance), and it is also similar to other species within the family Euplectellidae. The closest COI match is *Docosaccusmaculatus* Kahn, Geller, Reiswig & Smith Jr., 2013 (91.5% similarity). Morphological characters are concordant with those of the family Euplectellidae, and in the phylogenetic analysis it is recovered within the Euplectellidae (Fig. 54), along with other species of *Holascus* Schulze, 1886, but poorly supported. Although the subfamilies were not recovered as monophylectic, it is very likely it belongs to the subfamily Euplectillinae based on its lophophytous form.

**Ecology.** This specimen was found anchored to the abyssal sediments of APEI 1 at 5202 m depth.

**Comparison with image-based catalogue.** A very similar Euplectellidae morphotype (i.e., Euplectellidae gen. indet., HEX_005) has been commonly encountered in seabed image surveys conducted across nodule fields areas of the eastern CCZ, but not in abyssal areas of the Kiribati EEZ. Kersken et al. (2019) collected a few specimens of HEX_005 at the APEI 3 (Northeastern CCZ), identified as *Corbitelladiscasterosa* based on morphological characters. *Corbitelladiscasterosa* is basiphytous, while our specimen is lophophytous, but this would hardly be distinguished from seabed images.

#### Genus *Docosaccus* Topsent, 1910

##### ﻿ Docosaccus

Taxon classificationAnimaliaLyssacinosidaEuplectellidae

sp. CCZ_021

D15A123C-7CC1-5E8A-A03C-D77BD6552E41

Fig. 58

###### Material.

Clarion-Clipperton Zone • 1 specimen; APEI 7; 5.1043°N, 141.8867°W; 4860 m deep; 25 May. 2018; Smith & Durden leg.; GenBank: ON400684 (COI), ON406635 (18S), ON406612 (16S), ON406598 (28S), ON411255 (ALG11); NHMUK 2022.6; Voucher code: CCZ_021.

###### Description.

**Figure 58.**
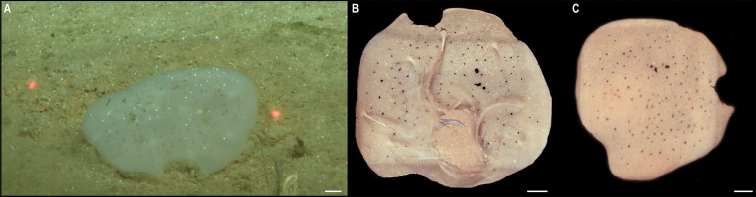
*Docosaccus* sp. CCZ_021 **A** in situ image **B** dermal surface **C** and atrial surface. Scale bars: 1 cm (**A, B, C**). Image attribution: Durden and Smith (**A**); Wiklund, Durden, Drennan, and McQuaid (**B, C**).

Single specimen; lophophytous sponge. Plate-like, flat, subcircular body; 8.7 cm at its longest axis, 1 mm thick (Fig. 58A). Colouration is yellowish. Atrial surface facing up (Fig. 58C), and dermal surface almost in contact with the seafloor, with basalia protruding from it and anchoring it to the sediment (Fig. 58B).

###### Remarks.

External morphological characters are concordant with the description of *D.maculatus* (Kahn et al. 2013). However, sequences for the 16S and COI genes from the holotype are 2.4% and 3.9% divergent (K2P distance), respectively, from the western CCZ specimen. Sequences from 18S and 28S do not match to sequences from *D.maculatus*. The species was described from Station M, off California, in the Pacific Ocean at depths of 3,953–4,000 m, but the genus was originally thought to be restricted to Antarctica (Kahn et al. 2013). This species has been recorded in the CCZ, in the eastern IFREMER contract area and in APEI 3, from 4905–4998 m depth (Kersken et al. 2019). The specimen collected in the western CCZ differs from the holotype of *D.maculatus* in having more parietal oscula, and is smaller, and therefore considered a different species. External morphological characters also differ from the other species reported for the CCZ, *D.nidulus* Kersken, Janussen & Martínez Arbizu, 2019.

###### Ecology.

This specimen was found anchored to abyssal sediments of APEI 7 at 4860 m depth.

###### Comparison with image-based catalogue.

A very similar *Docosaccus* sp. morphotype (i.e., *Docosaccusmaculatus* sp. inc., HEX_015) has been very frequently encountered in seabed image surveys conducted across nodule fields areas of the eastern CCZ and in abyssal areas of the Kiribati EEZ.

#### Genus *Holascus* Schulze, 1886

##### ﻿ Holascus

Taxon classificationAnimaliaLyssacinosidaEuplectellidae

stet. CCZ_078

449B3905-CEB5-58B4-92A7-1847091A9897

Fig. 59

###### Material.

Clarion-Clipperton Zone • 1 specimen; APEI 7; 5.0443°N, 141.8162°W; 48745 m deep; 28 May. 2018; Smith & Durden leg.; GenBank: ON400700 (COI), ON406636 (18S), ON406613 (16S), ON406599 (28S), ON411256 (ALG11); NHMUK 2022.7; CCZ_078.

###### Description.

**Figure 59.**
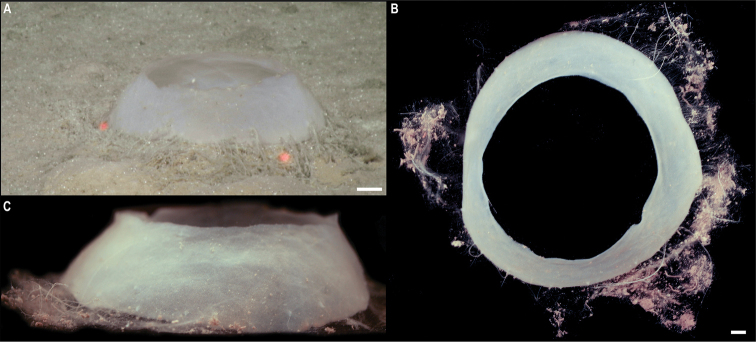
*Holascus* stet. CCZ_078 **A** in situ image **B** lateral view of specimen before preservation **C** top view. Scale bars: 2 cm (**A**); 1 cm (**B**). Image attribution: Durden and Smith (**A**); Wiklund, Durden, Drennan, and McQuaid (**B, C**).

Single specimen; lophophytous white sponge (Fig. 59A). Band-like body, collar formed from thin wall with two large openings, with lower opening being larger than upper opening (Fig. 59B, C). Body height of 4.8 cm, lower body diameter of 15.1 cm, and upper body diameter of 11.8 cm. basalia almost as long as the body height, protruding from the lower margin and anchoring it to the sediment (Fig. 59A).

###### Remarks.

Morphological external characters are concordant with the description of *Holascusspinosus* Kersken, Janussen & Martínez Arbizu, 2019, which was described from the IOM area in the CCZ. The closest genetic matches on GenBank for the 16S correspond to species within the genus *Holascus* (2.1–3.5% genetic divergence), with the holotype of *Holascusspinosus* being the closest match (2.1% genetic divergence). There are no 18S sequences available for *H.spinosus*, but it is 0.23% divergent from another species within the genus, *H.euonyx* (Lendenfeld, 1915), from which it differs morphologically. In the phylogenetic tree it was recovered, with low support, as sister to *H.spinosus* (Fig. 54), but 16S genetic divergence suggested these to be separate species.

###### Ecology.

This specimen was found anchored to abyssal sediments of APEI 7 at 4874 m depth.

###### Comparison with image-based catalogue.

A very similar *Holascus* sp. morphotype (i.e., *Holascus* sp. indet., HEX_014) has been commonly encountered in seabed image surveys conducted across nodule fields areas of the eastern CCZ and in abyssal areas of the Kiribati EEZ.

**Subfamily Bolosominae Tabachnick, 2002**

**Bolosominae stet. CCZ_198**

Fig. 60

**Material.** Clarion-Clipperton Zone • 1 specimen; APEI 1; 11.2518°N, 153.6053°W; 5205 m deep; 10 Jun. 2018; Smith & Durden leg.; GenBank: ON400728 (COI), ON406637 (18S), ON406614 (16S), ON406600 (28S); NHMUK 2022.10; Voucher code: CCZ_198.

**Figure 60.**
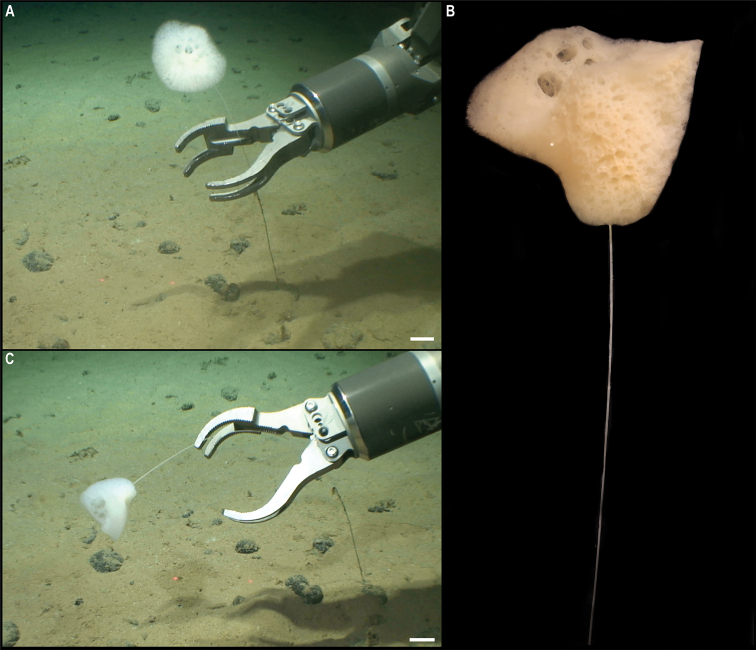
Bolosominae stet. CCZ_198 **A, C** in situ images **B** specimen before preservation. Scale bars: 5 cm (**A, C**). Image attribution: Durden and Smith (**A, C**); Wiklund, Durden, Drennan, and McQuaid (**B**).

**Description.** Single specimen; lophophytous white sponge (Fig. 60A, B). Body is cup- to bell-shaped, slightly wider than long (L = 10 cm, W = 11 cm), with a long, slender stalk (L = 70 cm) anchored to soft sediment. Central osculum with thick body walls.

**Remarks.** The closest match with the 18S sequence is the holotype of *Hyalostylusmicrofloricomus* Kersken, Janussen & Martínez Arbizu, 2019 (99.8%), described from the Heip Mountains in the GSR contract area in the CCZ at 3788 m depth (Kersken et al. 2019). However, in the phylogenetic tree (Fig. 54) it was recovered in a well-supported clade representing the subfamily Bolosominae, but subclades were not well supported and hence the specimen is not attributed to any genera.

**Ecology.** This specimen was collected on abyssal sediments of APEI 1 at 5205 m depth, and was anchored to the sediment.

**Comparison with image-based catalogue.** A very similar stalked sponge morphotype (i.e., Hexactinellidae ord. indet., HEX_026) has been encountered in seabed image surveys conducted at the eastern CCZ, but not in abyssal areas of Kiribati’s EEZ. In seabed images, HEX_026 highly resembles Hyalonema (Cyliconemaoida) campanula Lendenfeld, 1915, as identified by Kersken et al. (2019) based on morphological traits observed in specimens encountered in the eastern CCZ.

**Order Sceptrulophora Mehl, 1992**

**Family Euretidae Zittel, 1877**

**Subfamily Chonelasmatinae Schrammen, 1912**

#### Genus *Bathyxiphus* Schulze, 1899

##### ﻿ Bathyxiphus

Taxon classificationAnimaliaSceptrulophoraEuretidae

sp. CCZ_151

E67AFDBB-F71E-5AB3-A42E-60EABA91C59B

Figure 61

###### Material.

Clarion-Clipperton Zone • 1 specimen; APEI 4; 6.9881°N, 149.9321°W; 5001 m deep; 06 Jun. 2018; Smith & Durden leg.; GenBank: ON400713 (COI), ON406638 (18S), ON406615 (16S), ON406601 (28S); NHMUK 2022.11; Voucher code: CCZ_151.

###### Description.

**Figure 61.**
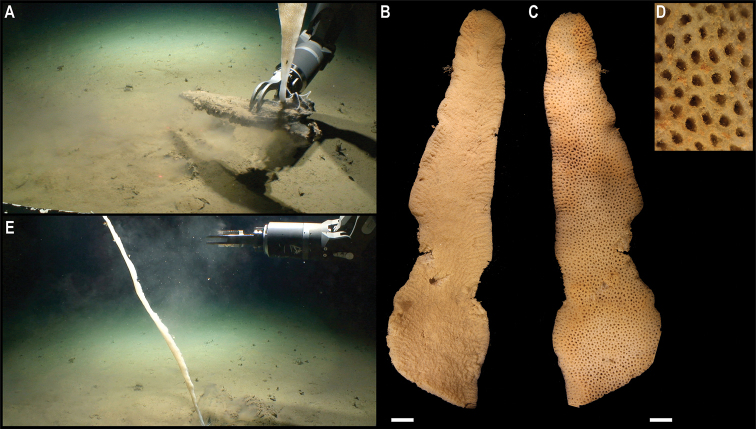
*Bathyxiphus* sp. CCZ_151. Specimen CCZ_151 **A, E** in situ images **B, C, D** specimen before preservation. Scale bars: 2 cm (**B, C**). Image attribution: Durden and Smith (**A, E**); Wiklund, Durden, Drennan, and McQuaid (**B–D**).

Single specimen; basiphytous sponge (Fig. 61A, E). Body white, elongated (L > 60 cm, W = 10 cm), thin (W = 11 mm), upright-blade shaped habitus (Fig. 61B, C, E), attached to, possibly, a beaked-whale rostrum covered in manganese crust (Fig. 61A).

###### Remarks.

Morphological characters are concordant with those of the genus, being very similar to *Bathyxiphussubtilis* Schulze, 1899, the only known species in the genus. However, a midrib has been suggested as a key morphological feature absent in the specimen presented here. The species was described from Isla Guadalupe at 1251 m depth, and was recently recorded in APEI 3 at 4914 m (Kersken et al. 2019). However, 16S sequences between the APEI 3 specimen and the western CCZ specimen are 3% divergent (K2P distance). Additionally, they were not recovered as monophyletic in the phylogenetic tree (Fig. 54) and hence considered different species. Measurements of total length were estimated from in situ images as only approx. half the specimen was recovered.

###### Ecology.

The specimen was found attached to a beaked-whale rostrum covered in polymetallic crust, on abyssal sediments of APEI 4 at 5001 m depth.

###### Comparison with image-based catalogue.

A similar *Bathyxiphus* sp. morphotype (i.e., *Bathyxiphus* sp. indet., HEX_025), though usually much smaller-sized, has been commonly encountered in seabed image surveys conducted across nodule fields areas of the eastern CCZ, but not in abyssal areas of the Kiribati EEZ.

## ﻿Discussion

The DeepCCZ expedition surveyed three APEIs on the western CCZ, targeting both abyssal seafloor and seamounts, and sampling the different megafaunal components. The ROV survey for benthic megafauna yielded a remarkably diverse collection from a small number of specimens, with 48 species from only 55 specimens (Table 1). Most species were represented by a single specimen, but whenever more than one specimen of the same species was collected, they were found at similar depths and same geoform (i.e., seamount, abyssal plain or seamount slope), even if collected in different sites. More than half of the species, 26, were sediment dwellers found both on seamounts and abyssal plains, mostly representing mobile megafauna, such as sea urchins, sea cucumbers, brittle stars, sea stars, a polychaete worm, and a jellyfish observed skimming the seafloor, and a single sessile species of cup coral. Eight species, mainly sponges, were found anchored to the sediment, five species were found attached to nodules, two species attached to polymetallic crust found on seamount slopes, and a single species of sponge was found attached to a fossilised beaked-whale rostrum covered in manganese crust. Additionally, six species were found attached to old glass sponge stalks, including scalpellids, crinoids, and actiniarians, with a few species co-occurring on the same sponge stalks.

Many of the taxa presented here had been encountered in image-based surveys from across the CCZ but never collected before, making our collections particularly important for improving taxonomic knowledge (Glover et al 2018). We were able to obtain genetic sequences for all but one specimen. The vast majority of taxa (29) could not be attributed to described species, with only four having been previously reported for the CCZ (Christodoulou et al. 2020; Glover et al. 2016b; Pawson and Foell 1986). The high number of delimited but undescribed species reported here highlights our still-limited knowledge on abyssal invertebrate megafauna in the CCZ, especially in the protected APEI regions, and illustrates the importance of publishing open DNA taxonomic data, even if full species identification and description are not always possible.

Although the CCZ is often considered a vast and relatively homogeneous abyssal plain, this region has substantial, ecologically important, seafloor heterogeneity (McQuaid et al. 2020; Washburn et al. 2021a). Variations in community structure and biodiversity have been documented across the region (Smith et al. 2021), mainly in the central and eastern CCZ, resulting from local and regional environmental conditions, such as topographical changes, food input, and nodule abundance (Stoyanova 2012; Vanreusel et al. 2016; Simon-Lledó et al. 2019c). While this study was not designed to investigate drivers of benthic megafaunal assemblages, some differences were observed between seamounts and abyssal plains. In other areas, seamounts have been found to harbour similar megafaunal taxonomic compositions as adjacent continental slopes (Clark et al. 2010). However, little overlap between seamount and abyssal plain taxa has been reported from surveys in the eastern CCZ (Cuvelier et al. 2020), and in western APEIs (Bribiesca-Contreras et al. 2021; Durden et al. 2021; Leitner et al. 2021). Due to the sampling strategy of maximising diversity, most species were represented by a single specimen, but whenever more than one specimen from the same species was collected, they were found in the same geoform (i.e., seamount or abyssal seafloor) even when collected in different APEIs (Table 1). Eight of the nine species that were assigned to previously described taxa were found in abyssal sites, except for *Calyptrophoradistolos* that was originally described from a seamount (Cairns 2018). This is possibly a result of seamounts being understudied and undersampled compared to abyssal plains.

While the lack of shared species between habitats in this study could be a result of undersampling, faunistic changes between habitats have been reported in the CCZ (Cuvelier et al. 2020; Durden et al. 2021). Differences between abyssal plains and seamounts in APEIs 4 and 7 (with only 16 and 19% overlap in Operational Taxonomic Units; OTUs) have been observed from environmental-DNA sampling of sediments, with most OTUs being rare and limited to small areas (Laroche et al. 2020). Similarly, image-based surveys in the western APEIs observed very few common morphotypes shared across habitats, with several rare morphotypes observed only once (Durden et al. 2021). In addition, Leitner et al. (2021) have argued that even if some species are shared between seamounts and the abyssal seafloor, the areal coverage of seamounts is a tiny proportion compared to the areal of abyssal seafloor in the CCZ, so seamounts could supply only a very limited number of propagules for recolonisation over ecological time scales. Previous findings and our data currently suggest that CCZ seamounts could not act as viable refugia for abyssal taxa and provide propagules for recolonisation of areas disturbed by mining. Following the principles of a precautionary approach, representative and suitable refuge areas for megafaunal communities needs to be defined and protected as source of propagules for recolonisation.

The study of deep-sea ecosystems presents several challenges, from the difficulties of collecting at great depths, to the resources required for an oceanographic survey, and the labour to document and describe all the collected material (Glover et al. 2018). With the pressing need to describe the biodiversity of these habitats, taxonomic studies that synthesise morphological, ecological, and genetic information, even for undescribed and indeterminate species (e.g., Dahlgren et al. 2016; Glover et al. 2016b; Amon et al. 2017a, b; Wiklund et al. 2017; Christodoulou et al. 2020) contribute fundamentally to the iterative building of biodiversity baselines (Engel et al. 2021; Glover et al. 2015). For instance, based on DNA sequences, we found two taxa with distributions likely spanning the entire CCZ, because they matched previously indeterminate juveniles collected in the eastern CCZ (*Ophiocymbiumtanyae* as Ophioscolecidae sp. 20 in Christodoulou et al. (2020), and cf. sp. CCZ_165 as Crinoidea sp. NHM_055 in Glover et al. (2016b)).

One of the limitations of this study is the scarcity of published barcodes for deep-see invertebrates. For instance, an environmental DNA study in the western APEIs found that only 25% and 1.5% of OTUs could be assigned to family level using reference libraries for 18S and COI, respectively (Laroche et al. 2020). We mainly targeted the barcoding gene COI because it has been used before to document biodiversity in the CCZ (e.g., Dahlgren et al. 2016; Glover et al. 2016b; Wiklund et al. 2017; Christodoulou et al. 2020). However, while the gene is useful for species delimitation in most groups, it is so variable that it does not accurately reflect phylogenetic relationships. Thus, in several cases it was not possible to assign our specimens to lower taxonomic levels based solely on genetic information because comparisons of our generated sequences to public databases showed that the closest taxa were only ~ 80% similar to multiple species belonging to different higher taxonomic ranks (Order or Family levels). Hence, additional genes were targeted to resolve higher rank relationships (16S, 18S, and 28S), but even for these genes, the lack of reference libraries hindered taxonomic placement. This is not surprising because the deep sea is severely undersampled and genetic sequences from bathyal and abyssal species are scarce in public databases. Even though there is much greater availability of genetic information for shallow water species, this did not inform our taxonomic assignments for most groups, because it has been found that bathyal and abyssal taxa often represent separate lineages from shallow-water ones (e.g., brittle stars: Bribiesca-Contreras et al. 2017; Christodoulou et al. 2019; isopods: Lins et al. 2012).

Additional challenges result from the documentation that COI and other mitochondrial genes show very little variation in anthozoan cnidarians (Hebert et al. 2003), preventing use of COI for species delimitation. For instance, the sequence of *Chrysogorgia* sp. CCZ_112 collected in APEI 4 differed from the COI sequence of *C.abludo* by a single nucleotide. Although the specimen was not assigned to the species because previous findings show that congeneric species of octocorals can share the same COI haplotype (McFadden et al. 2011), we also took a precautionary approach when assigning species to avoid overestimation of species ranges. In some syntheses, most deep-sea species have been assumed to have wide distributional ranges (McClain and Hardy 2010), likely resulting from the greater ease of discovering abundant, wide-ranging species than species with narrow distributions (Higgs and Attrill 2015), but also due to low or overlooked morphological variability that hinders our ability to discriminate species. Molecular studies have revealed different distinct lineages in some common, previously considered wide-ranging species. For instance, genetic sequences and further detailed morphological examination revealed at least two distinct species within the cosmopolitan *Psychropoteslongicauda* Théel, 1882 (Gubili et al. 2017; Gebruk et al. 2020). One represents the cosmopolitan species *P.pawsoni* Gebruk & Kremenetskaia in Gebruk et al. 2020 distributed in all four oceans, and the second represents a species only occurring in the northwest Pacific, *P.moskalevi* Gebruk & Kremenetskaia in Gebruk et al. 2020.

While detailed taxonomic studies can reveal an overlooked biodiversity in deep-sea taxa and are greatly improving our understanding of species ranges, they are time-consuming and usually target a small area or a few taxa (e.g., O’Loughlin and Ahearn 2005; Molodtsova and Opresko 2017; Herzog et al. 2018; Kersken et al. 2019). In contrast, the use of in situ seabed imagery to investigate megabenthic communities allows much larger area surveys, but at a lower taxonomic resolution that can potentially underestimate biodiversity and over-estimate species ranges. For instance, the sponge subfamilies Euplectellinae and Corbitellinae are differentiated by their mode of fixation to substrata. From seabed imagery, two hexactinellid sponges belonging to these subfamilies (Euplectellinae stet. CCZ_199 herein, and *Corbitelladiscasterosa* in (Kersken et al. 2019), respectively) were classified as the same morphotype because the mode of fixation cannot be observed when photographed from the top. However, the integration of both methods can provide key insights into species composition and connectivity of benthic communities across the CCZ, which are fundamental aspects to guide conservation strategies.

The alignment of in situ specimen images from this study with invertebrate morphotypes previously catalogued from (and standardised across) different seabed image surveys conducted in the CCZ (Amon et al. 2016; Simon-Lledó et al. 2019c, d, 2020; Cuvelier et al. 2020; Durden et al. 2021) provided preliminary insight into the connectivity of western megafauna populations. The 53 specimens that had an associated in situ image were classified into a total of 45 morphotypes, from which 11 represented new additions to the existing CCZ megafauna catalogue. Few morphotypes (9) were found to have wider distributions, being reported from both the Kiribati EEZ to the west of the CCZ, and from the eastern CCZ. Surprisingly, only two morphotypes were uniquely shared with Kiribati, while 16 were uniquely shared with the eastern CCZ. These results suggest, rather tentatively, that western CCZ megafauna communities may share a much larger species pool with (the more distant) eastern CCZ areas than with closer areas towards the west, like Kiribati’s abyss. However, it is important to note that a much larger sampling effort was previously conducted in eastern CCZ areas than around Kiribati. In addition, the ROV sampling conducted for this study was limited and the number of species identified in this study (48) is much lower than the number of morphotypes that have been identified from seabed imagery in the western CCZ (143; Durden et al. 2021). Further interpretation and synthesis of existing megafauna distribution data, as well as additional sampling, will expand and contextualise these preliminary observations.

## ﻿Conclusions

We provide the first megafaunal faunistic study from the western CCZ based on voucher specimens. Our findings indicate a high diversity, represented mostly by undescribed species of megafauna in the western CCZ with little overlap between abyssal plains and seamounts, and within similar habitats located in greater distances to one another. Further studies should aim to increase our knowledge of patterns of biodiversity across the entire CCZ in order to inform environmental management plans to protect its biodiversity. Our work also highlights the need for detailed taxonomic studies, not only within the CCZ, an area targeted for deep-sea mining, but in other bathyal, abyssal, and hadal regions. While species identification through genetic markers can facilitate the generation of species inventories, this is only achievable when genetic reference libraries are representative of the area and taxon of study, and these remains limited for the CCZ megafauna.

## Supplementary Material

XML Treatment for Laetmonice

XML Treatment for Trianguloscalpellum gigas

XML Treatment for Catherinum cf. albatrossianum

XML Treatment for Catherinum cf. novaezelandiae

XML Treatment for Fungiacyathus (Fungiacyathus) cf.fragilis

XML Treatment for Chrysogorgia

XML Treatment for Calyptrophora distolos

XML Treatment for Protoptilum

XML Treatment for Freyastera cf. tuberculata

XML Treatment for Freyastera

XML Treatment for Zoroaster

XML Treatment for Porphyrocrinus

XML Treatment for Plesiodiadema cf. globulosum

XML Treatment for Kamptosoma abyssale

XML Treatment for Molpadiodemas

XML Treatment for Molpadiodemas

XML Treatment for Synallactes

XML Treatment for Oneirophanta

XML Treatment for Oneirophanta cf. mutabilis

XML Treatment for Psychropotes verrucicaudatus

XML Treatment for Psychropotes dyscrita

XML Treatment for Benthodytes cf. sanguinolenta

XML Treatment for Benthodytes marianensis

XML Treatment for Peniagone leander

XML Treatment for Peniagone vitrea

XML Treatment for Psychronaetes

XML Treatment for Laetmogone cf. wyvillethomsoni

XML Treatment for Ophiocymbium tanyae

XML Treatment for Ophiocymbium cf. rarispinum

XML Treatment for Ophiuroglypha cf. irrorata

XML Treatment for Hyalonema

XML Treatment for Hyalonema

XML Treatment for Docosaccus

XML Treatment for Holascus

XML Treatment for Bathyxiphus

## Supplementary materials

Supplementary material 1 Table S1 Data type phylogenetic Explanation note Detail of sequences included in the phylogenetic analyses including details of voucher numbers, code, GenBank accession number for different genes, details on whether specimens included are part of type material. https://binary.pensoft.net/file/716388This dataset is made available under the Open Database License (http://opendatacommons.org/licenses/odbl/1.0/). The Open Database License (ODbL) is a license agreement intended to allow users to freely share, modify, and use this Dataset while maintaining this same freedom for others, provided that the original source and author(s) are credited.Guadalupe Bribiesca-Contreras, Thomas G. Dahlgren, Diva J. Amon, Stephen Cairns, Regan Drennan, Jennifer M. Durden, Marc P. Eléaume, Andrew Hosie, Antonina Kremenetskaia, Kirsty McQuaid, Timothy D. O’Hara, Muriel Rabone, Erik Simon-Lledó, Craig R. Smith, Les Watling, Helena Wiklund, Adrian G. Glover

## References

[B1] Agassiz A (1881) Report on the Echinoidea dredged by H.M.S. Challenger during the years 1873–1876. Report on the Scientific Results of the Voyage of HMS Challenger during the years 1873–76. Zoology (Jena, Germany) 3: 1–321.

[B2] Agassiz A (1898) Reports on the dredging operations off the west coast of Central America to the Galápagos, to the west coast of México, and in the Gulf of California, in charge of Alexander Agassiz, carried on by the U.S. Fish Commission Streamer "Albatross", during 1891, Lieut. Commander Z. L. Tanner, U.S.N., Commanding. XXIII. Preliminary report on the Echini. Bulletin of the Museum of Comparative Zoology 32: 71–86.

[B3] Agassiz A (1904) The Panamic deep sea echini. Memoirs of the Museum of Comparative Zoology at Harvard College 31: 1–243.

[B4] Amon DJ Ziegler AF Dahlgren TG Glover AG Goineau A Gooday AJ Wiklund H Smith CR (2016) Insights into the abundance and diversity of abyssal megafauna in a polymetallic-nodule region in the eastern Clarion-Clipperton Zone. Scientific Reports 6(1): 1–12. 10.1038/srep30492 27470484 PMC4965819

[B5] Amon DJ Ziegler AF Drazen JC Grischenko AV Leitner AB Lindsay DJ Voight JR Wicksten MK Young CM Smith CR (2017a) Megafauna of the UKSRL exploration contract area and eastern Clarion-Clipperton Zone in the Pacific Ocean: Annelida, Arthropoda, Bryozoa, Chordata, Ctenophora, Mollusca. Biodiversity Data Journal 14598: e14598. 10.3897/BDJ.5.e14598 PMC5565845 28874906

[B6] Amon DJ Ziegler AF Kremenetskaia A Mah CL Mooi R O’Hara T Pawson DL Roux M Smith CR (2017b) Megafauna of the UKSRL exploration contract area and eastern Clarion-Clipperton Zone in the Pacific Ocean: Echinodermata. Biodiversity Data Journal 11794: e11794. 10.3897/BDJ.5.e11794 PMC5515089 28765722

[B7] Anderson OF (2016) A review of New Zealand and southeast Australian echinothurioids (Echinodermata: Echinothurioida)–excluding the subfamily Echinothuriinae–with a description of a new species of *Tromikosoma* Zootaxa 4092: 451–488. 10.11646/zootaxa.4092.4.1 27394469

[B8] Bonifácio P Martínez Arbizu P Menot L (2020) Alpha and beta diversity patterns of polychaete assemblages across the nodule province of the eastern Clarion-Clipperton Fracture Zone (equatorial Pacific). Biogeosciences 17(4): 865–886. 10.5194/bg-17-865-2020

[B9] Bouckaert R Heled J Kuhnert D Vaughan T Wu CH Xie D Suchard MA Rambaut A Drummond AJ (2014) BEAST 2: A software platform for Bayesian evolutionary analysis. PLoS Computational Biology 10(4): e1003537. 10.1371/journal.pcbi.1003537 PMC3985171 24722319

[B10] Bribiesca-Contreras G Verbruggen H Hugall AF O’Hara TD (2017) The importance of offshore origination revealed through ophiuroid phylogenomics. Proceedings. Biological Sciences 284(1858): e20170160. 10.1098/rspb.2017.0160 PMC5524485 28679721

[B11] Bribiesca-Contreras G Dahlgren TG Horton T Drazen JC Drennan R Jones DOB Leitner AB McQuaid KA Smith CR Taboada S Wiklund H Glover AG (2021) Biogeography and Connectivity Across Habitat Types and Geographical Scales in Pacific Abyssal Scavenging Amphipods. Frontiers in Marine Science 8: e1028. 10.3389/fmars.2021.705237

[B12] Cairns SD (2015) New abyssal Primnoidae (Anthozoa: Octocorallia) from the Clarion-Clipperton Fracture Zone, equatorial northeastern Pacific. Marine Biodiversity 46(1): 141–150. 10.1007/s12526-015-0340-x

[B13] Cairns SD (2018) Primnoidae (Cnidaria: Octocorallia: Calcaxonia) of the Okeanos Explorer expeditions (CAPSTONE) to the central Pacific. Zootaxa 4532(1): 1–43. 10.11646/zootaxa.4532.1.1 30647372

[B14] Castresana J (2000) Selection of conserved blocks from multiple alignments for their use in phylogenetic analysis. Molecular Biology and Evolution 17(4): 540–552. 10.1093/oxfordjournals.molbev.a026334 10742046

[B15] Christodoulou M O’Hara TD Hugall AF Arbizu PM (2019) Dark Ophiuroid Biodiversity in a Prospective Abyssal Mine Field. Current Biology 29(22): 3909–3912. 10.1016/j.cub.2019.09.012 31630951

[B16] Christodoulou M O’Hara T Hugall AF Khodami S Rodrigues CF Hilario A Vink A Martinez Arbizu P (2020) Unexpected high abyssal ophiuroid diversity in polymetallic nodule fields of the northeast Pacific Ocean and implications for conservation. Biogeosciences 17(7): 1845–1876. 10.5194/bg-17-1845-2020

[B17] Chow S Konishi K Mekuchi M Tamaki Y Nohara K Takagi M Niwa K Teramoto W Manabe H Kurogi H Suzuki S Ando D Jinbo T Kiyomoto M Hirose M Shimomura M Kurashima A Ishikawa T Kiyomoto S (2016) DNA barcoding and morphological analyses revealed validity of Diadema clarki Ikeda, 1939 (Echinodermata, Echinoidea, Diadematidae). ZooKeys 585: 1–16. 10.3897/zookeys.585.8161 PMC4857035 27199601

[B18] Clark MR Rowden AA Schlacher T Williams A Consalvey M Stocks KI Rogers AD O’Hara TD White M Shank TM Hall-Spencer JM (2010) The ecology of seamounts: Structure, function, and human impacts. Annual Review of Marine Science 2(1): 253–278. 10.1146/annurev-marine-120308-081109 21141665

[B19] Cuvelier D Ribeiro PA Ramalho SP Kersken D Martinez Arbizu P Colaço A (2020) Are seamounts refuge areas for fauna from polymetallic nodule fields? Biogeosciences 17(9): 2657–2680. 10.5194/bg-17-2657-2020

[B20] Dahlgren TG Wiklund H Rabone M Amon DJ Ikebe C Watling L Smith CR Glover AG (2016) Abyssal fauna of the UK-1 polymetallic nodule exploration area, Clarion-Clipperton Zone, central Pacific Ocean: Cnidaria. Biodiversity Data Journal 4: e9277. 10.3897/BDJ.4.e9277 PMC5018120 27660533

[B21] Danovaro R Fanelli E Aguzzi J Billett D Carugati L Corinaldesi C Dell’Anno A Gjerde K Jamieson AJ Kark S McClain C Levin L Levin N Ramirez-Llodra E Ruhl H Smith CR Snelgrove PVR Thomsen L Van Dover CL Yasuhara M (2020) Ecological variables for developing a global deep-ocean monitoring and conservation strategy. Nature Ecology & Evolution 4(2): 181–192. 10.1038/s41559-019-1091-z 32015428

[B22] Dohrmann M (2018) Progress in glass sponge phylogenetics: A comment on Kersken et al. (2018). Hydrobiologia 843(1): 51–59. 10.1007/s10750-018-3708-7

[B23] Downey ME (1986) Revision of the Atlantic Brisingida (Echinodermata: Asteroidea), with description of a new genus and family. Smithsonian Contributions to Zoology 435(435): 1–57. 10.5479/si.00810282.435

[B24] Durden JM Putts M Bingo S Leitner AB Drazen JC Gooday AJ Jones DOB Sweetman AK Washburn TW Smith CR (2021) Megafaunal Ecology of the Western Clarion Clipperton Zone. Frontiers in Marine Science 2021: 722. 10.3389/fmars.2021.671062

[B25] Eléaume M Bohn J-M Roux M Améziane N (2012) Stalked crinoids (Echinodermata) collected by the R/V Polarstern and Meteor in the south Atlantic and in Antarctica. Zootaxa 3425(1): 1–22. 10.11646/zootaxa.3425.1.1

[B26] Engel MS Ceríaco LMP Daniel GM Dellapé PM Löbl I Marinov M Reis RE Young MT Dubois A Agarwal I Lehmann AP Alvarado M Alvarez N Andreone F Araujo-Vieira K Ascher JS Baêta D Baldo D Bandeira SA Barden P Barrasso DA Bendifallah L Bockmann FA Böhme W Borkent A Brandão CRF Busack SD Bybee SM Channing A Chatzimanolis S Christenhusz MJM Crisci JV D`elía G Da Costa LM Davis SR De Lucena CAS Deuve T Fernandes Elizalde S Faivovich J Farooq H Ferguson AW Gippoliti S Gonçalves FMP Gonzalez VH Greenbaum E Hinojosa-Díaz IA Ineich I Jiang J Kahono S Kury AB Lucinda PHF Lynch JD Malécot V Marques MP Marris JWM McKellar RC Mendes LF Nihei SS Nishikawa K Ohler A Orrico VGD Ota H Paiva J Parrinha D Pauwels OSG Pereyra MO Pestana LB Pinheiro PDP Prendini L Prokop J Rasmussen C Rödel M-O Rodrigues MT Rodríguez SM Salatnaya H Sampaio Í Sánchez-García A Shebl MA Santos BS Solórzano-Kraemer MM Sousa ACA Stoev P Teta P Trape J-F Dos Santos CV-D Vasudevan K Vink CJ Vogel G Wagner P Wappler T Ware JL Wedmann S Zacharie CK (2021) The taxonomic impediment: A shortage of taxonomists, not the lack of technical approaches. Zoological Journal of the Linnean Society 193(2): 381–387. 10.1093/zoolinnean/zlab072

[B27] Fauchald K (1977) The polychaete worms. Definitions and keys to the orders, families and genera. Natural History Museum of Los Angeles County, Science Series 28, 188 pp. https://repository.si.edu/bitstream/handle/10088/3435/PinkBook-plain.pdf

[B28] Forero-Mejia AC Molodtsova T Östman C Bavestrello G Rouse GW (2019) Molecular phylogeny of Ceriantharia (Cnidaria: Anthozoa) reveals non-monophyly of traditionally accepted families. Zoological Journal of the Linnean Society 190(2): 397–416. 10.1093/zoolinnean/zlz158

[B29] Gallo ND Cameron J Hardy K Fryer P Bartlett DH Levin LA (2015) Submersible- and lander-observed community patterns in the Mariana and New Britain trenches: Influence of productivity and depth on epibenthic and scavenging communities. Deep-sea Research. Part I, Oceanographic Research Papers 99: 119–133. 10.1016/j.dsr.2014.12.012

[B30] Gebruk AV Kremenetskaia A Rouse GW (2020) A group of species “*Psychropoteslongicauda*” (Psychropotidae, Elasipodida, Holothuroidea) from the Kuril-Kamchatka Trench area (North-West Pacific). Progress in Oceanography 180: e102222. 10.1016/j.pocean.2019.102222

[B31] Glover A Dahlgren T Wiklund H Mohrbeck I Smith C (2015) An End-to-End DNA Taxonomy Methodology for Benthic Biodiversity Survey in the Clarion-Clipperton Zone, Central Pacific Abyss. Journal of Marine Science and Engineering 4(1): 2. 10.3390/jmse4010002

[B32] Glover A Dahlgren T Taboada S Paterson G Wiklund H Waeschenbach A Cobley A Martínez P Kaiser S Schnurr S Khodami S Raschka U Kersken D Stuckas H Menot L Bonifacio P Vanreusel A Macheriotou L Cunha M Hilário A Rodrigues C Colaço A Ribeiro P Błażewicz M Gooday A Jones D Billett D Goineau A Amon D Smith C Patel T McQuaid K Spickermann R Brager S (2016a) The London Workshop on the Biogeography and Connectivity of the Clarion-Clipperton Zone. Research Ideas and Outcomes 2: e10528. 10.3897/rio.2.e10528

[B33] Glover AG Wiklund H Rabone M Amon DJ Smith CR O’Hara T Mah CL Dahlgren TG (2016b) Abyssal fauna of the UK-1 polymetallic nodule exploration claim, Clarion-Clipperton Zone, central Pacific Ocean: Echinodermata. Biodiversity Data Journal 4: e7251. 10.3897/BDJ.4.e7251 PMC4759440 26929713

[B34] Glover AG Wiklund H Chen C Dahlgren TG (2018) Managing a sustainable deep-sea ‘blue economy’ requires knowledge of what actually lives there. eLife 7: e41319. 10.7554/eLife.41319 PMC6257809 30479272

[B35] Gong L Li X Xiao N He L Zhang H Wang Y (2020) Rediscovery of the abyssal species *Peniagoneleander* Pawson & Foell, 1986 (Holothuroidea: Elasipodida: Elpidiidae): the first record from the Mariana Trench area. Journal of Oceanology and Limnology 38(4): 1319–1327. 10.1007/s00343-020-0067-9

[B36] Gooday AJ Durden JM Holzmann M Pawlowski J Smith CR (2020a) Xenophyophores (Rhizaria, Foraminifera), including four new species and two new genera, from the western Clarion-Clipperton Zone (abyssal equatorial Pacific). European Journal of Protistology 75: 125715. 10.1016/j.ejop.2020.125715 32585572

[B37] Gooday AJ Durden JM Smith CR (2020b) Giant, highly diverse protists in the abyssal Pacific: Vulnerability to impacts from seabed mining and potential for recovery. Communicative & Integrative Biology 13(1): 189–197. 10.1080/19420889.2020.1843818 33312334 PMC7714518

[B38] Gubili C Ross E Billett DSM Yool A Tsairidis C Ruhl HA Rogacheva A Masson D Tyler PA Hauton C (2017) Species diversity in the cryptic abyssal holothurian *Psychropoteslongicauda* (Echinodermata). Deep-sea Research. Part II, Topical Studies in Oceanography 137: 288–296. 10.1016/j.dsr2.2016.04.003

[B39] Hebert PD Ratnasingham S deWaard JR (2003) Barcoding animal life: Cytochrome c oxidase subunit 1 divergences among closely related species. Proceedings. Biological Sciences 270: S96–S99. 10.1098/rsbl.2003.0025 PMC1698023 12952648

[B40] Hein JR Koschinsky A Kuhn T (2020) Deep-ocean polymetallic nodules as a resource for critical materials. Nature Reviews Earth & Environment 1: 158–169. https://www.nature.com/articles/s43017-020-0027-0

[B41] Herzog S Amon DJ Smith CR Janussen D (2018) Two new species of *Sympagella* (Porifera: Hexactinellida: Rossellidae) collected from the Clarion-Clipperton Zone, East Pacific. Zootaxa 4466(1): 152–163. 10.11646/zootaxa.4466.1.12 30313444

[B42] Hestetun JT Vacelet J Boury-Esnault N Borchiellini C Kelly M Rios P Cristobo J Rapp HT (2016) The systematics of carnivorous sponges. Molecular Phylogenetics and Evolution 94: 327–345. 10.1016/j.ympev.2015.08.022 26416707

[B43] Higgs ND Attrill MJ (2015) Biases in biodiversity: Wide-ranging species are discovered first in the deep sea. Frontiers in Marine Science 2(61): 1–8. 10.3389/fmars.2015.00061

[B44] Hoek PPC (1883) Report on the Cirripedia collected by H.M.S. Challenger during the years 1873–76. Report on the Scientific Results of the Voyage of HMS Challenger during the years 1873–76 Zoology 8: 1–169. 10.5962/bhl.title.12873

[B45] Horton T Marsh L Bett BJ Gates AR Jones DOB Benoist NMA Pfeifer S Simon-Lledó E Durden JM Vandepitte L Appeltans W (2021) Recommendations for the Standardisation of Open Taxonomic Nomenclature for Image-Based Identifications. Frontiers in Marine Science 62: 1–13. 10.3389/fmars.2021.620702

[B46] Howell KL Rogers AD Tyler PA Billett DSM (2004) Reproductive isolation among morphotypes of the Atlantic seastar species *Zoroasterfulgens* (Asteroidea: Echinodermata). Marine Biology 144(5): 977–984. 10.1007/s00227-003-1248-8

[B47] International Seabed Authority I (2020) Workshop Report: Deep CCZ Biodiversity Synthesis Workshop Friday Harbor, Washington, USA, 1–4 October 2019. Kingston, Jamaica.

[B48] Jones DO Kaiser S Sweetman AK Smith CR Menot L Vink A Trueblood D Greinert J Billett DS Arbizu PM Radziejewska T Singh R Ingole B Stratmann T Simon-Lledó E Durden JM Clark MR (2017) Biological responses to disturbance from simulated deep-sea polymetallic nodule mining. PLoS ONE 12: e0171750. 10.1371/journal.pone.0171750 PMC5298332 28178346

[B49] Jones DOB Simon-Lledó E Amon DJ Bett BJ Caulle C Clément L Connelly DP Dahlgren TG Durden JM Drazen JC Felden J Gates AR Georgieva MN Glover AG Gooday AJ Hollingsworth AL Horton T James RH Jeffreys RM Laguionie-Marchais C Leitner AB Lichtschlag A Menendez A Paterson GLJ Peel K Robert K Schoening T Shulga NA Smith CR Taboada S Thurnherr AM Wiklund H Young CR Huvenne VAI (2021) Environment, ecology, and potential effectiveness of an area protected from deep-sea mining (Clarion Clipperton Zone, abyssal Pacific). Progress in Oceanography 197: e102653. 10.1016/j.pocean.2021.102653

[B50] Kahn AS Geller JB Reiswig HM Smith KL (2013) *Bathydoruslaniger* and *Docosaccusmaculatus* (Lyssacinosida; Hexactinellida): Two new species of glass sponge from the abyssal eastern North Pacific Ocean. Zootaxa 3646(4): 386–400. 10.11646/zootaxa.3646.4.4 26213771

[B51] Kahn AS Pennelly CW McGill PR Leys SP (2020) Behaviors of sessile benthic animals in the abyssal northeast Pacific Ocean. Deep-sea Research. Part II, Topical Studies in Oceanography 173: e104729. 10.1016/j.dsr2.2019.104729

[B52] Kaiser S Smith CR Arbizu PM (2017) Editorial: Biodiversity of the Clarion Clipperton Fracture Zone. Marine Biodiversity 47(2): 259–264. 10.1007/s12526-017-0733-0

[B53] Kamenskaya OE Melnik VF Gooday AJ (2013) Giant protists (xenophyophores and komokiaceans) from the Clarion-Clipperton ferromanganese nodule field (eastern Pacific). Biology Bulletin Reviews 3(5): 388–398. 10.1134/S2079086413050046 23136792

[B54] Katoh K Rozewicki J Yamada KD (2019) MAFFT online service: Multiple sequence alignment, interactive sequence choice and visualization. Briefings in Bioinformatics 20(4): 1160–1166. 10.1093/bib/bbx108 28968734 PMC6781576

[B55] Kersken D Janussen D Arbizu PM (2019) Deep-sea glass sponges (Hexactinellida) from polymetallic nodule fields in the Clarion-Clipperton Fracture Zone (CCFZ), northeastern Pacific: Part II–Hexasterophora. Marine Biodiversity 49(2): 947–987. 10.1007/s12526-018-0880-y

[B56] Kremenetskaia A Gebruk A Alt CHS Budaeva N (2021) New and Poorly Known Species of *Peniagone* (Holothuroidea, Elpidiidae) from the Northwest Pacific Ocean with Discussion on Phylogeny of the Genus. Diversity (Basel) 13(11): e541. 10.3390/d13110541

[B57] Lanfear R Frandsen PB Wright AM Senfeld T Calcott B (2017) PartitionFinder 2: New methods for selecting partitioned models of evolution for molecular and morphological phylogenetic analyses. Molecular Biology and Evolution 34: 772–773. https://academic.oup.com/mbe/article-abstract/34/3/772/2738784 28013191 10.1093/molbev/msw260

[B58] Laroche O Kersten O Smith CR Goetze E (2020) Environmental DNA surveys detect distinct metazoan communities across abyssal plains and seamounts in the western Clarion Clipperton Zone. Molecular Ecology 29(23): 4588–4604. 10.1111/mec.15484 32452072 PMC7754508

[B59] Leitner AB Drazen JC Smith CR (2021) Testing the Seamount Refuge Hypothesis for Predators and Scavengers in the Western Clarion-Clipperton Zone. Frontiers in Marine Science 8: 1–22. 10.3389/fmars.2021.63630535685121

[B60] Li YN Xiao N Zhang LP Zhang H (2018) *Benthodytesmarianensis*, a new species of abyssal elasipodid sea cucumbers (Elasipodida: Psychropotidae) from the Mariana Trench area. Zootaxa 4462(3): 443–450. 10.11646/zootaxa.4462.3.10 30314039

[B61] Lins LS Ho SY Wilson GD Lo N (2012) Evidence for Permo-Triassic colonization of the deep sea by isopods. Biology Letters 8(6): 979–982. 10.1098/rsbl.2012.0774 23054914 PMC3497147

[B62] Martynov AV (2010) Reassessment of the classification of Ophiuroidea (Echinodermata), based on morphological characters. I. General character evaluation and delineation of the families Ophiomyxidae and Ophiacanthidae. Zootaxa 2697(1): 1–154. 10.11646/zootaxa.2697.1.1

[B63] McClain CR Hardy SM (2010) The dynamics of biogeographic ranges in the deep sea. Proceedings. Biological Sciences 277(1700): 3533–3546. 10.1098/rspb.2010.1057 20667884 PMC2982252

[B64] McFadden CS Benayahu Y Pante E Thoma JN Nevarez PA France SC (2011) Limitations of mitochondrial gene barcoding in Octocorallia. Molecular Ecology Resources 11(1): 19–31. 10.1111/j.1755-0998.2010.02875.x 21429097

[B65] McQuaid KA Attrill MJ Clark MR Cobley A Glover AG Smith CR Howell KL (2020) Using habitat classification to assess representativity of a protected area network in a large, data-poor area targeted for deep-sea mining. Frontiers in Marine Science 7: e1066. 10.3389/fmars.2020.558860

[B66] Messing CG (2016) *Porphyrocrinusdaniellalevyae* n. sp. (Echinodermata: Crinoidea), a sea lily from the tropical western Atlantic with a unique crown pattern. Zootaxa 4147(1): 1–35. 10.11646/zootaxa.4147.1.1 27515601

[B67] Miller AK Kerr AM Paulay G Reich M Wilson NG Carvajal JI Rouse GW (2017) Molecular phylogeny of extant Holothuroidea (Echinodermata). Molecular Phylogenetics and Evolution 111: 110–131. 10.1016/j.ympev.2017.02.014 28263876

[B68] Mironov AN (1971) [Soft sea urchins of the family Echinothuriidae collected by the R/V “Vityaz” and the “Academician Kurchatov” in the Pacific and Indian Oceans.] Trudy Instituta Okeanologii Akademii Nauk SSSR 92: 317–325.

[B69] Mironov AN Minin KV Dilman AB (2015) Abyssal echinoid and asteroid fauna of the North Pacific. Deep-sea Research. Part II, Topical Studies in Oceanography 111: 357–375. 10.1016/j.dsr2.2014.08.006

[B70] Molodtsova TN Opresko DM (2017) Black corals (Anthozoa: Antipatharia) of the Clarion-Clipperton Fracture Zone. Marine Biodiversity 47(2): 349–365. 10.1007/s12526-017-0659-6

[B71] Mooi R Constable H Lockhart S Pearse J (2004) Echinothurioid phylogeny and the phylogenetic significance of *Kamptosoma* (Echinoidea: Echinodermata). Deep-sea Research. Part II, Topical Studies in Oceanography 51(14–16): 1903–1919. 10.1016/j.dsr2.2004.07.020

[B72] O’Hara TD Stöhr S Hugall AF Thuy B Martynov A (2018) Morphological diagnoses of higher taxa in Ophiuroidea (Echinodermata) in support of a new classification. European Journal of Taxonomy 2018(416): 1–35. 10.5852/ejt.2018.416

[B73] O’Loughlin PM Ahearn C (2005) A review of pygal-furrowed Synallactidae (Echinodermata: Holothuroidea), with new species from the Antarctic, Atlantic and Pacific oceans. Memoirs of the Museum of Victoria 62(2): 147–179. 10.24199/j.mmv.2005.62.5

[B74] O’Loughlin PM Paulay G Davey N Michonneau F (2011) The Antarctic region as a marine biodiversity hotspot for echinoderms: Diversity and diversification of sea cucumbers. Deep-sea Research. Part II, Topical Studies in Oceanography 58(1–2): 264–275. 10.1016/j.dsr2.2010.10.011

[B75] OBIS (2022) Ocean Biodiversity Information System. Intergovernmental Oceanographic Commission of UNESCO.

[B76] Pante E Watling L (2011) *Chrysogorgia* from the New England and Corner Seamounts: Atlantic–Pacific connections. Journal of the Marine Biological Association of the United Kingdom 92(5): 911–927. 10.1017/S0025315411001354

[B77] Pawson DL (1983) *Psychronaeteshanseni*, a new genus and species of elasipodan sea cucumber from the eastern central Pacific (Echinodermata: Holothurioidea). Proceedings of the Biological Society of Washington 96: 154–159.

[B78] Pawson DL Foell EJ (1986) *Peniagoneleander* new species, an abyssal benthopelagic sea cucumber (Echinodermata: Holothuroidea) from the eastern central Pacific Ocean. Bulletin of Marine Science 38: 293–299.

[B79] Poltarukha OP Mel’Nik VF (2012) New records of deep-sea barnacles (Cirripedia: Thoracica: Scalpelliformes) from the Clarion-Clipperton region, Pacific Ocean. Zootaxa 3297: 34–40. 10.11646/zootaxa.3297.1.2

[B80] Provoost P Bosch S (2020) robis: Ocean Biodiversity Information System (OBIS) Client. R package version 239.

[B81] Rex MA Etter RJ (2010) Deep-sea Biodiversity. Harvard University Press, Cambridge, MA.

[B82] Rodriguez E Barbeitos MS Brugler MR Crowley LM Grajales A Gusmao L Haussermann V Reft A Daly M (2014) Hidden among sea anemones: The first comprehensive phylogenetic reconstruction of the order Actiniaria (Cnidaria, Anthozoa, Hexacorallia) reveals a novel group of hexacorals. PLoS ONE 9(5): e96998. 10.1371/journal.pone.0096998 PMC4013120 24806477

[B83] Shalaeva K Boxshall G (2014) An illustrated catalogue of the scalpellid barnacles (Crustacea: Cirripedia: Scalpellidae) collected during the HMS “Challenger” expedition and deposited in the Natural History Museum, London. Zootaxa 3804(1): 1, 4–63. 10.11646/zootaxa.3804.1.1 24871151

[B84] Simon-Lledó E Bett BJ Huvenne VAI Koser K Schoening T Greinert J Jones DOB (2019a) Biological effects 26 years after simulated deep-sea mining. Scientific Reports 9(1): 1–13. 10.1038/s41598-019-44492-w 31142831 PMC6541615

[B85] Simon-Lledó E Bett BJ Huvenne VAI Schoening T Benoist NMA Jeffreys RM Durden JM Jones DOB (2019b) Megafaunal variation in the abyssal landscape of the Clarion Clipperton Zone. Progress in Oceanography 170: 119–133. 10.1016/j.pocean.2018.11.003 30662100 PMC6325340

[B86] Simon-Lledó E Bett BJ Huvenne VAI Schoening T Benoist NMA Jones DOB (2019c) Ecology of a polymetallic nodule occurrence gradient: Implications for deep-sea mining. Limnology and Oceanography 64(5): 1883–1894. 10.1002/lno.11157 31598009 PMC6774340

[B87] Simon-Lledó E Thompson S Yool A Flynn A Pomee C Parianos J Jones DOB (2019d) Preliminary Observations of the Abyssal Megafauna of Kiribati. Frontiers in Marine Science 605: e605. 10.3389/fmars.2019.00605

[B88] Simon-Lledó E Pomee C Ahokava A Drazen JC Leitner AB Flynn A Parianos J Jones DOB (2020) Multi-scale variations in invertebrate and fish megafauna in the mid-eastern Clarion Clipperton Zone. Progress in Oceanography 187: e102405. 10.1016/j.pocean.2020.102405

[B89] Sladen WP (1889) Report on the Asteroidea. Report on the scientific results of the voyage of H.M.S. Challenger during the years 1873–1876. Zoology (Jena, Germany) 30: 1–893.

[B90] Smith CR Clark MR Goetze E Glover AG Howell KL (2021) Editorial: Biodiversity, Connectivity and Ecosystem Function Across the Clarion-Clipperton Zone: A Regional Synthesis for an Area Targeted for Nodule Mining. Frontiers in Marine Science 8: e797516. 10.3389/fmars.2021.797516

[B91] Stamatakis A (2006) RAxML-VI-HPC: Maximum likelihood-based phylogenetic analyses with thousands of taxa and mixed models. Bioinformatics (Oxford, England) 22(21): 2688–2690. 10.1093/bioinformatics/btl446 16928733

[B92] Stöhr S O’Hara TD (2021) Deep-sea Ophiuroidea (Echinodermata) from the Danish Galathea II Expedition, 1950–52, with taxonomic revisions. Zootaxa 4963(3): 505–529. 10.11646/zootaxa.4963.3.6 33903543

[B93] Stoyanova V (2012) Megafaunal diversity associated with deep-sea nodule-bearing habitats in the eastern part of the Clarion-Clipperton Zone, NE Pacific. International Multidisciplinary Scientific GeoConference SGEM 1: 645–652. 10.5593/sgem2012/s03.v1032

[B94] Théel H (1879) Preliminary report on the Holothuroidae of the exploring voyage of the H.M.S. Challenger under professor Sir C. Wyville Thomson F.R.S., Part 1. Bihang Till K Svenska Vet Akad Handlingar 5: 1–20.

[B95] Théel H (1882) Report on the Holothuroidea dredged by H.M.S. ‘Challenger’ during the years 1873–76. Part i. Report on the scientific results of the voyage of H.M.S. Challenger during the years 1873–1876. Zoology (Jena, Germany) 4: 1–176.

[B96] Thomson CW Murray J (1885) Report on the Scientific Results of the Voyage of H.M.S. Challenger during the years 1873–1876. Zoology (Jena, Germany) 115(48): 30.

[B97] Tilot V (2006) Biodiversity and Distribution of the Megafauna. In: Commission IO (Ed.) Technical Series 69. Project Unesco COI/Min, Vlanderen.

[B98] Vanreusel A Hilario A Ribeiro PA Menot L Arbizu PM (2016) Threatened by mining, polymetallic nodules are required to preserve abyssal epifauna. Scientific Reports 6(1): 1–6. 10.1038/srep26808 27245847 PMC4887785

[B99] Washburn TW Jones DOB Wei C-L Smith CR (2021a) Environmental Heterogeneity Throughout the Clarion-Clipperton Zone and the Potential Representativity of the APEI Network. Frontiers in Marine Science 8: e319. 10.3389/fmars.2021.661685

[B100] Washburn TW Menot L Bonifácio P Pape E Błażewicz M Bribiesca-Contreras G Dahlgren TG Fukushima T Glover AG Ju SJ Kaiser S Yu OH Smith CR (2021b) Patterns of macrofaunal biodiversity across the Clarion-Clipperton Zone: An area targeted for deep-sea mining. Frontiers in Marine Science 8: e250. 10.3389/fmars.2021.626571

[B101] Wedding LM Friedlander AM Kittinger JN Watling L Gaines SD Bennett M Hardy SM Smith CR (2013) From principles to practice: A spatial approach to systematic conservation planning in the deep sea. Proceedings. Biological Sciences 280(1773): e20131684. 10.1098/rspb.2013.1684 PMC3826217 24197407

[B102] Wiklund H Taylor JD Dahlgren TG Todt C Ikebe C Rabone M Glover AG (2017) Abyssal fauna of the UK-1 polymetallic nodule exploration area, Clarion-Clipperton Zone, central Pacific Ocean: Mollusca. ZooKeys 707: 1–46. 10.3897/zookeys.707.13042 PMC5674146 29118626

[B103] Wilson GDF (2017) Macrofauna abundance, species diversity and turnover at three sites in the Clipperton-Clarion Fracture Zone. Marine Biodiversity 47(2): 323–347. 10.1007/s12526-016-0609-8

[B104] Xiao N Gong L Kou Q Li X (2019) *Psychropotesverrucicaudatus*, a new species of deep-sea holothurian (Echinodermata: Holothuroidea: Elasipodida: Psychropotidae) from a seamount in the South China Sea. Bulletin of Marine Science 95(3): 421–430. 10.5343/bms.2018.0041

[B105] Zevina GB Poltarukha OP (2014) Deep-sea fauna of European seas: An annotated species check-list of benthic invertebrates living deeper than 2000 m in the seas bordering Europe. Cirripedia. Invertebrate Zoology 11: 101–111. http://oceanrep.geomar.de/id/eprint/28024

[B106] Zhang R Wang C Zhou Y Zhang H (2019) Morphology and molecular phylogeny of two new species in genus *Freyastera* (Asteroidea: Brisingida: Freyellidae), with a revised key to close species and ecological remarks. Deep-sea Research. Part I, Oceanographic Research Papers 154: e103163. 10.1016/j.dsr.2019.103163

